# The impact of digital intelligence technologies on innovation performance: Evidence from specialized, refined, differential and innovative enterprises

**DOI:** 10.1371/journal.pone.0339567

**Published:** 2026-02-10

**Authors:** Tian Zhao, Fa Zhang, Guoliang Zhuo, Qingqing Zhang, Qingwen Yuan

**Affiliations:** 1 School of Economics and Management, Shandong University of Science and Technology, Qingdao, Shandong, China; 2 School of Economics, Jinan University, Guangzhou, Guangdong, China; 3 Department of Finance and Economics, Shandong University of Science and Technology, Jinan, Shandong, China; USTC: University of Science and Technology of China, CHINA

## Abstract

The integration of digital and intelligent technologies has created new opportunities for the innovative growth of Specialized, Refined, Differential, and Innovative (SRDI) enterprises in China. This study examines SRDI enterprises listed on the Shanghai/Shenzhen A-share from 2014 to 2022. A three-stage Data Envelopment Analysis, incorporating a knowledge breadth framework, is employed to evaluate their innovation performance. Furthermore, an empirical analysis is conducted to assess the influence of digital intelligence on innovation outcomes. The results indicate that: (1) Digital intelligence, measured by datafication, digitization, and the adoption of intelligent technologies, significantly improves the innovation performance of SRDI enterprises, with intelligent technologies exerting the greatest effect; (2) Digital intelligence effectively mitigates financing constraints, allowing firms to allocate more resources to innovation; and (3) Heterogeneity analysis shows that the positive impact of digital intelligence is more pronounced among non-state-owned enterprises, manufacturing firms, and those located in Eastern China. These findings provides valuable guidance for policymakers seeking to develop targeted and differentiated strategies to enhance the innovation capacity of SRDI enterprises. In particular, the results underscore the role of digital intelligence in easing financing constraints. By accounting for firm-level heterogeneity, this study offers a robust theoretical and empirical foundation for policy design and delivers actionable recommendations for enterprises aiming to optimize resource allocation and strengthen innovation capabilities through digital transformation.

## 1. Introduction

Over the past four decades, China’s economy has undergone profound transformation driven by market-oriented reforms and global integration. As the traditional resource-driven growth model has gradually given way to innovation-led development, innovation has become the primary driver of economic progress. In this shifting landscape, small and medium-sized enterprises (SMEs) have played a critical role in advancing technology, upgrading industries, and strengthening ecological resilience. Among them, SRDI enterprises have emerged as a leading force, with innovation positioned at the center of their developmental strategies. The SRDI concept was introduced by the Ministry of Industry and Information Technology(MIIT) in July 2011. SRDI enterprises are defined by four core attributes. Specialization refers to a focus on core business activities, enhancing capabilities in specialized production, service delivery, and supply chain integration. Refinement emphasizes precision in manufacturing, management, and operations to achieve superior quality and cost efficiency in niche markets. Differentiation leverages unique resources and regional culture, thereby fostering distinctive competitive advantages. Innovation drives technological progress, managerial improvements, and business model evolution, fostering sustainable growth and market competitiveness. Since 2019, the ministry has recognized six cohorts comprising more than 10,000 national-level SRDI “little giant” enterprises. Over the past decade, these enterprises have become a driving force in China’s economic development. They underpin China’s mid-to high-end manufacturing chain and constitute a key pillar for high-quality and innovation-driven development [1,2].

According to a report by the Ministry of Industry and Information Technology of China, SMEs account for over 90% of all enterprises in the country, contribute more than 70% of technological innovations, and generate over 60% of the GDP. Within this group, SRDI enterprises exhibit particularly strong market competitiveness and innovation capacity. They are a critical component of China’s manufacturing strategy, helping to address key technological bottlenecks. Despite their importance, SRDI enterprises face notable challenges, especially in innovation and financing. Limited access to credit is a major constraint, as financial institutions often view smaller firms as high-risk borrowers. This reluctance restricts the capital available for research and development (R&D), innovation activities, and business expansion. The rapid advancement of digital intelligence technologies has introduced new opportunities to address these constraints. Data, with its non-rivalrous nature and potential for unlimited growth, has become a pivotal driver of productivity. By enabling rapid and cost-efficient resource acquisition, digital intelligence can ease financing pressures and support sustained R&D investment, thereby strengthening innovation capacity. Focusing on SRDI enterprises is therefore critical, as they represent a highly innovative segment of SMEs and play a central role in overcoming core technological challenges. Examining how these enterprises adopt and benefit from digital intelligence provides valuable insight into the broader mechanisms that shape SME innovation. Understanding this relationship is essential for promoting the high-quality development of China’s SMEs in the context of ongoing technological and industrial transformation.

Existing research has largely focused on the relationship between digitalization on corporate innovation [3,4], while relatively few studies have examined how digital technologies empower enterprises and influence innovation performance. This study addresses two key gaps. First, it investigates how digital intelligence can alleviate financing constraints and thereby improve the quality of innovation in SRDI enterprises. Second, it explores which specific technologies and institutional contexts amplify this effect. To achieve these objectives, the analysis draws on data from listed SRDI companies between 2014 and 2022, applying a three-stage Data Envelopment Analysis (DEA) combined with a knowledge breadth approach to identify the mechanisms through which digital empowerment affects innovation performance. The findings aim to provide actionable insights for government agencies and financial institutions, supporting informed decision-making, promoting the healthy development of local digital technologies, and strengthening the innovation capacity of SRDI enterprises.

This paper examines the influence of digital intelligence technology composition on innovation performance within China’s SRDI enterprises. Three principal contributions are made. First, this study not only constructs an overall digital intelligence index but also disaggregates it into five distinct technological domains (cloud computing, artificial intelligence, blockchain, big data, and digital technology applications), thereby overcoming aggregation bias and revealing substantial heterogeneity in their effects on innovation performance. The results show that while all five domains improve innovation efficiency, only cloud computing, artificial intelligence, and digital technology applications significantly enhance innovation quality. Second, whereas existing studies have predominantly focused on manufacturing firms or ordinary SMEs [5,6], often treating digital technologies as a single aggregated indicator [7] or examining only one dimension of innovation performance (efficiency or quality) [8,9], this study specifically targets China’s SRDI enterprises—a group explicitly designated as national policy priorities for industrial upgrading and high-quality development. Using a near-complete sample of listed SRDI firms from 2014 to 2022, innovation performance is measured along both efficiency (using three-stage DEA) and quality (captured by patent knowledge breadth) dimensions, thereby rectifying the conventional overemphasis on innovation quantity. Third, the study establishes causal relationships through instrumental variables combined with a multi-period difference-in-differences design that exploits an exogenous policy shock. It further identifies financing constraints as a key mediating mechanism and reveals substantial heterogeneity, with positive effects concentrated in non-state-owned, manufacturing, and Eastern-region firms, whereas blockchain exerts suppressive effects in state-owned and non-manufacturing enterprises. These findings on the heterogeneous effects of the five digital intelligence technologies may also inform technology adoption decisions for SMEs in other emerging economies. Specifically, while all five technologies can enhance innovation efficiency and cloud computing, artificial intelligence, and digital applications are particularly effective for improving innovation quality among resource-constrained firms, blockchain may exert suppressive effects on innovation quality in certain contexts. This underscores that not all digital intelligence technologies are uniformly beneficial and highlights the need for cautious, context-specific adoption.

## 2. Literature review

### 2.1. Research on the empowerment of digital intelligence

A growing body of research has examined how digital intelligence technologies influence innovation performance. The term “digital intelligence” was first introduced in a 2015 report by Peking University’s Zhiben Foundation, where it was described as the organic integration of digital wisdom and wise digitization. In this context, empowerment refers to granting greater authority and autonomy to individuals or teams in the execution of tasks [10]. As an emerging strategic organizational form at the forefront of technological and industrial transformation, digital intelligence has become a key driver of high-quality development in Chinese enterprises [11]. The expansion of digital government, for example, has been shown to enhance the quality of China’s urban export products by reducing information costs, fostering urban innovation, and optimizing resource allocation [12]. Evidence from other emerging economies also suggests positive effects of digital technologies on SME innovation performance. For example, studies report that SMEs adopting cloud-based management systems and digital finance in India, family-owned firms implementing digital strategies in Brazil, and enterprises combining digitalization with knowledge management for green innovation in Vietnam tend to exhibit improved innovation performance [13–15]. Despite its increasing application, there is no consensus in the academic literature on a unified definition of digital empowerment. Drawing on previous studies [16,17], this research defines digital empowerment as the process by which organizations or individuals use digital intelligence technologies to generate, acquire, and apply knowledge as a strategic asset, thereby strengthening the capabilities of actors and enabling them to achieve defined objectives. For SRDI enterprises, digital empowerment is often realized in open innovation environments. In such settings, these enterprises deploy digital technologies to build and maintain resource databases, reduce operational and management costs, improve decision-making efficiency, and enhance innovation performance.

Building on the research of previous scholars [18,19], this study examines the concept of digital empowerment through three core dimensions: datafication, digitization, and intelligence. First, from the perspective of foundational resource theory, digital empowerment begins with resource datafication, whereby enterprises transform both internal and external resources into data that can serve as production factors. These resources are then arranged, integrated, and optimized through digital technologies and tools. In this framework, effective resource orchestration is essential for capturing digital opportunities. Insufficient utilization of available resources remains a major obstacle to digital transformation, particularly for SMEs [20]. For SRDI enterprises, redefining and reconfiguring foundational resources through digital technologies is a critical step toward establishing a competitive position in the emerging technological and economic paradigm.

Second, from the perspective of business processes, process digitization involves embedding digital technologies and intelligent hardware into traditional production workflows. This transformation reshapes manufacturing and processing models into fully digital systems. The resulting process optimization and automation lower costs, increase efficiency, and alter workforce composition by raising demand for skilled labor while reducing reliance on routine, low-skill tasks [21]. Such process-oriented digitization also improves organizational agility, enhancing the ability of enterprises, particularly SMEs, to respond effectively to crises [22].

Finally, from the perspective of organization, organizational intelligence centers on enhancing human capital through digital technologies. Enterprises strengthen their capacity to identify, transform, organize, and share knowledge, embedding these capabilities into reform- and innovation-driven strategies. This enables more informed decision-making, fosters continuous learning, and supports long-term competitiveness in dynamic market environments.

### 2.2. Research on the Specialized, Refined, Differential, and Innovative enterprises

The measurement of innovation performance in SRDI depends on both the choice indicators and the selection of measurement methods. In terms of indicators, most studies rely on patent-related metrics, including a number of patent applications or grants [23,24] and patent citation counts [25]. Patents are advantageous for evaluating innovation performance because they directly reflect the intensity of a firm’s innovative activity over a given period. Citation counts provide an additional measure by indicating the influence and quality of patents within specific technological fields. Patent data also possess qualities of internationalization and standardization, as application, examination, and granting processes are governed by universally applicable systems and regulations, enabling reliable cross-regional and cross-industry comparisons. In addition to patent-based measures, some scholars adopt product-oriented indicators such as total profit [26], return on total assets [27], or structured measurement scales [28]. Product-based measures reflect market acceptance of innovation outcomes and can incentivize firms to prioritize the development of commercially successful products, thereby fostering sustained innovation.

Methods for evaluating innovation efficiency include parametric, non-parametric, and qualitative comparative approaches. Parametric methods such as Stochastic Frontier Analysis (SFA) provide a strong economic foundation for explaining efficiency differences but require specific assumptions about the production function, which can reduce accuracy if these assumptions do not align with real-world conditions. Non-parametric methods such as DEA avoid such assumptions and apply linear programming to handle multiple inputs and outputs, making them well suited to complex systems. However, DEA may account for external environmental factors or random disturbances, potentially leading to discrepancies between measured and actual efficiency. The three-stage DEA method addresses these limitations by incorporating environmental variables and stochastic factors, resulting in more accurate efficiency assessments. Qualitative approaches, such as fuzzy-set qualitative comparative analysis (FsQCA), are also increasingly used to analyze complex issues from a dynamic perspective. Among these, SFA, DEA, and FsQCA have been widely applied in measuring innovation efficiency [29,30].

### 2.3. Research on the role of digital technologies in promoting enterprise innovation

With the rapid development of the digital economy and intelligent technologies, SRDI enterprises in China are increasingly integrating digital intelligence tools into all stages of the innovation process. The essence of digital empowerment lies in driving enterprise innovation through digital and intelligent technologies, encompassing technology research and development, market analysis, production optimization, and management upgrades. By leveraging advanced digital platforms and tools, enterprises can achieve comprehensive improvements, ranging from data-driven decision-making to intelligent manufacturing, personalized services, supply chain optimization, innovative business models, organizational restructuring, and enhanced security management. The adoption of digital intelligence technologies can also mitigate information asymmetries between enterprises and financial institutions, thereby improving credit allocation efficiency [31,32] and boosting innovation output [33]. In particular, big data analytics and artificial intelligence enable SRDI enterprises to more accurately identify market needs, strengthen technological research and development, and improve competitiveness. Existing literature has explored the role of digital intelligence in enterprise innovation primarily from the perspectives of digital platforms, digital transformation, and digital finance.

The emergence of digital platforms and knowledge-sharing systems is reshaping traditional models of technological innovation, shifting the process from isolated, independent nodes to interconnected networks. This networked approach exerts a decisive influence on both the scope and direction of technological advancement [34]. However, traditional industries may face a “digital divide” due to limitations in funding and skilled personnel, underscoring the need for effective digital governance to support enterprise innovation. Empirical evidence from Wang and He [35] and Guo et al. [36] shows the digital transformation significantly improves enterprise innovation performance. Research on digital finance further indicates that it can raise the innovation levels of SRDI enterprises by alleviating resource constraints [37]. Furthermore, studies on artificial intelligence demonstrate that AI technology contributes positively to the innovation efforts of SRDI enterprises [38].

Existing studies on digital empowerment and SRDI innovation exhibit three limitations:

(1) Quantity-over-quality bias: Current research places greater emphasis on the impact of digital technologies on innovation quantity (e.g., patent counts [23,24]), creating a “quantity-driven, quality-deficient” perception. This focus neglects the role of digital intelligence in improving innovation quality, which is particularly critical for SRDI enterprises operating within China’s policy-driven innovation ecosystem.
(2) Oversimplified mechanisms: While the technical upgrading effects of digitalization are well documented [29,30], limited attention has been given to how SRDI enterprises strategically integrate different forms of digital intelligence (e.g., AI combined with Internet of Things) to enhance innovation performance [3]. Underexplored heterogeneity: The varied impacts of specific digital intelligence technologies (e.g., machine learning versus blockchain) on SRDI innovation have not been systematically examined, especially in relation to differences in adoption levels.

This study addresses these gaps by assessing the quality of SRDI enterprises’ patents through knowledge breadth metrics and by analyzing how distinct digital intelligence pathways influence innovation outcomes.

## 3. Theoretical mechanism

Within the digital economy, the integration of datafication, digitization, and intelligent technologies into the innovation activities of SRDI enterprises is becoming increasingly prominent. This integration reshapes resource configurations, technological capabilities, and organizational structures, generating positive effects across multiple dimensions. These three drivers exhibit distinct structural characteristics yet interact synergistically, forming an organic system that collectively enhances the innovation performance of SRDI enterprises in China.

First, the digital intelligence economy drives resource innovation in SRDI enterprises in China through datafication. Data, as a core element of innovation, has become a major driving force behind resource innovation. According to theory of technological-economic paradigms [39], data resources are characterized by low costs, abundant supply, and broad applicability. From a raw materials perspective, data represents a new production factor that, unlike traditional energy sources, is both low-cost and renewable, thereby improving innovation performance and optimizing resource use. From the standpoint of infrastructure investment, digital infrastructures such as “middle platforms” enable enterprises to identify and restructure valuable external data into intelligent modules. These modules can improve employee innovation efficiency and increase the likelihood of breakthrough innovations through code rewriting or module recombination. By renting cloud-based “middle platform” services, enterprises can reduce fixed capital investment, accelerate capital turnover, and improve innovation performance. From a labor input perspective, key technical and interdisciplinary talent yield higher returns than low-skilled labor. Such personnel can operate complex equipment efficiently and, with the aid of digital intelligence technologies, redirect labor toward more creative tasks, accelerating production, decision-making, and innovation efficiency.

Second, the digital intelligence economy stimulates technological innovation in SRDI enterprises through digitization. To address the complexities of innovation in the digital era, these enterprises have shifted from closed to open and distributed innovation models. The integration of these models increases both the speed and quality of innovation and accelerates the commercialization of research outcomes. In terms of efficiency, Internet of Things and big data technologies facilitate the integration and efficient use of global innovation resources. By forming technology alliances, SRDI enterprises can share resources, leverage technological advantages, reduce costs and accelerate innovation. From a quality perspective, open and distributed innovation strengthens digital and intelligent capabilities, allowing firms to break away from traditional linear innovation models. The resulting open innovation environment heightens awareness among market participants of the value of open collaboration, further improving innovation quality. Regarding commercialization, open innovation broadens resource access, while distributed innovation fosters alliances that integrate enterprise resources and provide funding, technology, and labor, shortening the time from research and development to market entry and improving the conversion rate of technological achievements.

Third, the digital intelligence economy facilitates organizational upgrading in SRDI enterprises through intelligent technologies. In dynamic markets, maintaining competitiveness without being displaced by new entrants is a key challenge [40]. As these SMEs accumulate diverse resources, they may experience a “resource curse” due to complexity [41]. The “innovator’s dilemma” [42] highlights how companies can become resistant to disruptive innovations due to an overreliance on existing resources. As a driving force of China’s economic and social development, SRDI enterprises typically possess strong capabilities for disruptive innovation. However, once they attain market leadership, they face the threat of disruption from new technologies and business models. For such high-performing enterprises, disruptive innovation may not appear financially rational, and organizational inertia often steers them toward incremental improvements. The emergence of intelligent technologies, such as artificial intelligence and cloud computing, provides solutions for overcoming these barriers. These tools can improve organizational structures, enhance resource management efficiency [43,44], and help firms avoid the pitfalls of the “innovator’s dilemma.”

## 4. Method and data

### 4.1. Sample selection and data sources

To ensure robustness, the sample includes enterprises from all six batches of SRDI enterprises publicly announced by the Ministry of Industry and Information Technology of China. Data covering the period 2014–2022 were compiled from four primary sources:

(1) Financial Data: Obtained for Shanghai and Shenzhen A-share listed SRDI enterprises. The starting year of 2014 aligns with China’s 2013 policy defining SRDI criteria, ensuring full coverage of eligible firms across all six MIIT-recognized batches. Financial variables were drawn from CSMAR and the iFinD Financial Data Terminal; SRDI firm lists (batches 1–6) came from MIIT announcements. The dataset includes inputs, outputs, and environmental factors for the three stage DEA, plus R and D expenditure, operating revenue, ST/*ST status, and region, industry, and ownership classifications to control for heterogeneity. We reported the variable level data source mapping in S4 Appendix, and we reported the complete SRDI roster in S7 Appendix.
(2) Patent Records: Collected from the China National Intellectual Property Administration(CNIPA) (over 2 million entries), including patent type, authorization date, and International Patent Classification (IPC) codes. All data were retrieved from the CNIPA Patent Search and Analysis Database, and IPC fields follow CNIPA records in accordance with the World Intellectual Property Organization (WIPO) IPC scheme. We reported the detailed calculation procedures for the IPC-based knowledge breadth, with worked examples and Stata code, in S3 Appendix.
(3) Digital Intelligence Metrics: Extracted from annual reports using keyword frequency (e.g., AI, blockchain, cloud computing) to objectively map firms’ strategic adoption of digital technologies. We reported the construction workflow and diagnostics for the Digital Intelligence Index in S2 Appendix, and we reported the complete keyword dictionary in S1 Appendix.
(4) Industry Classifications: Based on the Global Industry Classification Standard (GICS) from the China’s National Bureau of Statistics. GICS was selected for its market-driven focus, aligning with SRDI growth objectives. We reported the full GICS taxonomy in this study in S6 Appendix.

Data Integration Process: A standardized protocol was applied: (1) matching enterprise names and stock codes across datasets; (2) excluding ST/*ST firms, enterprises in underdeveloped regions (e.g., Tibet), and those with critical missing data; and (3) applying linear interpolation to address remaining missing values. The final balanced panel dataset comprises 1,110 SRDI enterprises (across six policy batches) with 9,990 firm-year observations, providing temporal and contextual consistency for analyzing the impact of digital intelligence on innovation.

### 4.2. Model setting and variable definition

To examine the impact of digital intelligence on the innovation performance of SRDI enterprises, the following baseline regression models are specified:


$$\begin{array}{c} {IE}_{i,t}=\alpha_{\text{0}}+\alpha_{\text{1}}{Dig}_{i,t}+\alpha_{\text{1}}{Top\text{1}}_{i,t}+\alpha_{\text{2}}{Board}_{i,t}+\alpha_{\text{3}}{HHI}_{i,t}+\alpha_{\text{4}}{TAT}_{i,t}\\ +\;\alpha_{\text{5}}{Cycle}_{i,t}+\alpha_{\text{6}}{OCF}_{i,t}+\alpha_{\text{7}}{ROA}_{i,t}+\sum Industry+\sum Year+\varepsilon_{i,t} \end{array}$$ (1)

$$\begin{array}{c} {IG}_{i,t}=\beta_{\text{0}}+\beta_{\text{1}}{Dig}_{i,t}+\beta_{\text{2}}{Top\text{1}}_{i,t}+\beta_{\text{3}}{Board}_{i,t}+\beta_{\text{4}}{HHI}_{i,t}+\beta_{\text{5}}{TAT}_{i,t}\\ +\;\beta_{\text{6}}{Cycle}_{i,t}+\beta_{\text{7}}{OCF}_{i,t}+\beta_{\text{8}}{ROA}_{i,t}+\sum Industry+\sum Year+\epsilon_{i,t} \end{array}$$ (2)

In these models, IEi,t and IGi,t represent the dependent variables, representing innovation efficiency and innovation quality, respectively, where subscripts *i* and *t* refers to the firm and year, respectively. The key explanatory variable *Dig*_*i,t*_, is the level of digital intelligence adoption by the enterprise. This is quantified through the extent of application of various digital technologies, including cloud computing (Cloud), big data (Big Data), artificial intelligence (AI), blockchain (Block), and digital technology applications (DT). The control variables include: *Top1*_*i,t*_, *Board*_*i,t*_,*HHI*_*i,t*_, *TAT*_*i,t*_, *Cycle*_*i,t*_, *OCF*_*i,t*_ and *ROA*_*i,t*_. Both models incorporate industry fixed effects and year fixed effects to control for unobserved heterogeneity, and $\varepsilon_{i,t}$ and $\varphi_{i,t}$, represent the error terms.

To thoroughly investigate the intrinsic relationship between digital intelligence and the innovation performance of SRDI enterprises, and to verify both its direct impact on innovation performance and the mediating role of financing constraints, this study employs a two-step empirical approach [45,46]. The corresponding regression models are specified as follows:


$$\begin{array}{c} {\text{SA}}_{i,t}=\rho_{\text{0}}+{\rho_{\text{1}}Dig}_{i,t}+\rho_{\text{2}}{Top\text{1}}_{i,t}+\rho_{\text{3}}{Board}_{i,t}+\rho_{\text{4}}{HHI}_{i,t}+\rho_{\text{5}}{TAT}_{i,t}+\rho_{\text{6}}{Cycle}_{i,t}\\ +\;\rho_{\text{7}}{OCF}_{i,t}+\rho_{\text{8}}{ROA}_{i,t}+\sum Industry+\sum Year+\tau_{i,t} \end{array}$$ (3)

In this context, *SA*_*i,t*_ represents the mediating variable, measuring the degree of financing constraints faced by the enterprise. while $\tau_{i,t}$ denotes the model’s error term. All other variables are defined consistently with those in Equation (1). Equation (3) estimates the effect of digital intelligence on financing constraints, with the corresponding coefficient indicating the intensity of this influence.

On December 31, 2014, the National Development and Reform Commission of China issued the “Notice on the Implementation Plan for Cloud Computing Projects” (hereinafter referred to as the “Notice”). This policy provided a substantial external shock that serves as an exogenous event to address potential endogeneity concerns in this study. Leveraging this event, we construct a multi-period quasi-natural experiment. Specifically, a detailed manual screening process using a digital intelligence frequency database was employed to identify the timing of points the “Notice” policy’s impact to individual enterprises. The emergence of digital intelligence-related terminology in a firm’s annual reports is treated as the pivotal impact point for constructing the multi-period quasi-natural experiment. We subsequently develop models (4) and (5) to conduct a Difference-in-Differences analysis.


$$\begin{array}{c} {IE}_{it}=\varphi_{\text{0}}+\varphi_{\text{1}}{Dig\_Shock}_{it}+\varphi_{\text{2}}{Top\text{1}}_{i,t}+\varphi_{\text{3}}{Board}_{i,t}+\varphi_{\text{4}}{HHI}_{i,t}+\varphi_{\text{5}}{TAT}_{i,t}\\ +\;\varphi_{\text{6}}{Cycle}_{i,t}+\varphi_{\text{7}}{OCF}_{i,t}+\varphi_{\text{8}}{ROA}_{i,t}+\sum Industry+\sum Year+\omega_{i,t} \end{array}$$ (4)

$$\begin{array}{c} {IG}_{it}=\theta_{\text{0}}+\theta_{\text{1}}{Dig\_Shokc}_{it}+\theta_{\text{2}}{Top\text{1}}_{i,t}+\theta_{\text{3}}{Board}_{i,t}+\theta_{\text{4}}{HHI}_{i,t}+\theta_{\text{5}}{TAT}_{i,t}\\ +\;\theta_{\text{6}}{Cycle}_{i,t}+\theta_{\text{7}}{OCF}_{i,t}+\theta_{\text{8}}{ROA}_{i,t}+\sum Industry+\sum Year+\omega_{i,t} \end{array}$$ (5)

In models (4) and (5), the variable Dig_Shock serves as the explanatory variable, indicating the time point at which digital intelligence terminology appears in the annual reports of the sample enterprises. For example, if an SRDI enterprise first includes such terminology in its reports in 2015, Dig_Shock is assigned a value of 1 for all years following 2015 and 0 for the years prior. The definitions of the remaining dependent and control variables are consistent with those established in model (1). Both models also control for year and industry fixed effects to account for unobserved heterogeneity across time and sectors.

The dependent variable in this study is innovation performance. Following the approaches of Zhang and Zheng [47] and Cao et al. [48] on assessing patent quality in Chinese enterprises, innovation performance is measured along two dimensions: innovation efficiency (IE) and innovation quality (IQ). Innovation efficiency is evaluated using a three-stage DEA method, implemented in DEAP 2.1 (DEA stages) and FRONTIER 4.1 (stochastic-frontier adjustment), which examines the input-output dynamics of SRDI enterprises listed in China. We reported the detailed three stage computation steps in S5 Appendix. Innovation quality is calculated by examining the breadth of knowledge based on the IPC codes of patents authorized in the a given year at a macro level. To address potential endogeneity concerns, the analysis controls for industry-level knowledge breadth when estimating the patent knowledge breadth of individual firms. Due to the substantial variability of IPC codes for design patents, the knowledge breadth method is less effective for measuring their scope. Therefore, the study focuses exclusively on invention and utility model patents when constructing the innovation quality indicator. The computed innovation efficiency and innovation quality metrics are summarized in Table 1. From 2014 to 2022, both measures exhibit a consistent upward trend among SRDI enterprises.

**Table 1:** Evaluation results of innovation performance.

Year	Innovation Efficiency						Innovation Quality
	DEA			Three-Stage DEA			
	crste	vrste	scale	crste	vrste	scale	
2014	0.126	0.225	0.693	0.088	0.837	0.109	4.801
2015	0.121	0.179	0.695	0.031	0.833	0.04	5.707
2016	0.088	0.108	0.82	0.114	0.842	0.141	6.657
2017	0.114	0.211	0.579	0.118	0.845	0.144	6.793
2018	0.109	0.163	0.695	0.105	0.884	0.121	8.520
2019	0.116	0.186	0.661	0.126	0.904	0.143	9.101
2020	0.154	0.222	0.735	0.143	0.853	0.176	10.833
2021	0.146	0.214	0.72	0.131	0.788	0.176	12.869
2022	0.147	0.217	0.712	0.14	0.819	0.179	14.917
Mean	0.125	0.192	0.701	0.111	0.845	0.137	8.911

Note: crste, vrste, and scale represent comprehensive technical efficiency, pure technical efficiency, and scale efficiency, respectively

**Fig 1:**
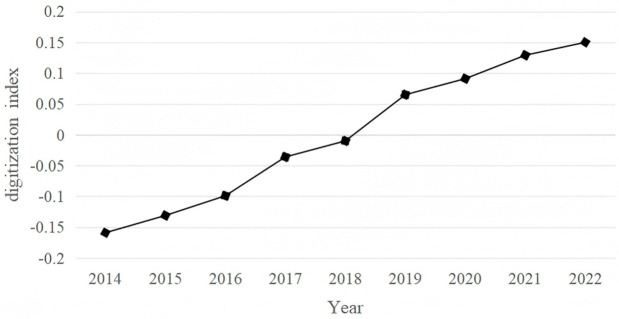
Average digitization index.

The independent variable in this study is the digitization index (*Dig*) for SRDI enterprises. Following the approach of Wang and Guo [49], who examined the relationship between digitization on the innovation quality of listed manufacturing firms in China, this study evaluates the drivers of innovation performance in SRDI enterprises across three dimensions: datafication, digitization, and intelligentization. The index is constructed using the frequency of terms related to cloud computing, big data, artificial intelligence, blockchain, and digital technology applications identified in annual reports. These frequencies are treated as discrete variables. To reduce dimensionality, principal component analysis (PCA) is applied, retaining components with eigenvalues ≥ 1 and a cumulative variance contribution rate of at least 85%. In the econometric model, the digitization index is log-transformed after adding 1, yielding lnDig, which represents the level of digitization. Additionally, the frequencies for each of the five dimensions are individually log-transformed to measure the respective levels of cloud computing, big data, artificial intelligence, blockchain, and digital technology applications. The computed digitization index reveals that the average digitization level of SRDI enterprises in China increased by approximately 200% from 2014 to 2022, with a consistent upward growth trend year-on-year (Fig 1).

The horizontal axis represents the year, while the vertical axis denotes the Digital Intelligence Index.

This study uses financing constraints as the mediating variable, based on the rationale that digital intelligence can help alleviate the “financing difficulty” faced by enterprises, thereby promoting innovation. Since financing constraints are not directly observable, prior research has typically relied on indirect single indicators such as debt ratio, dividend payout ratio, and asset size. Other studies have constructed composite measures, including the KZ index, WW index, and SA index. Given that the KZ and WW indices contain certain endogenous variables, this study adopts the SA index as the measure of financing constraints, as it offers stronger exogeneity. The SA index is calculated as follows:


$${SA}_{i,t}=-\text{0}.\text{737}*{Size}_{i,t}+\text{0}.\text{043}*{Size}_{i,t}^{\text{2}}-\text{0}.\text{04}*{Age}_{i,t}$$ (6)

In this context, *Size* refers to the natural logarithm of the firm’s total assets and is used to measure enterprise scale. Age denotes the operational age of the enterprise, measured in years since its establishment. Sources: the iFinD Financial Data Terminal; SA index is manually computed using Eq. (6).

Several control variables are included to account for firm-specific characteristics that may influence innovation performance. Ownership concentration (*Top*1), measured by the shareholding ratio of the largest shareholder, captures the impact of ownership structure. Board size (*Board*), represented by the number of sitting directors, reflects corporate governance quality. Market competition (*HHI*) is measured using the Herfindahl–Hirschman Index of the industry to control for competitive pressures. Asset turnover ratio (*ATO*), calculated as the ratio of net operating revenue to average total assets, indicates the efficiency of asset utilization. Operating cycle (*Cycle*) measures the time from acquiring inventory to selling products and recovering cash, serving as a proxy for operational efficiency. Operating cash flow (*OCF*), the net cash flow from operating activities, controls for the firm’s liquidity and financial health. Finally, return on assets (ROA), calculated as the ratio of earnings before interest and taxes to average total assets, accounts for overall profitability and financial efficiency. All control variables are obtained or calculated based on data from the CSMAR database and the iFinD Financial Data Terminal.

### 4.3. Methodology and analytical framework

The analytical process of this study involves three steps. First, the three-stage DEA model is employed to measure the innovation efficiency of China’s SRDI listed enterprises from both input and output perspectives. Second, the knowledge breadth of patents is assessed based on their IPC codes to capture innovation quality. Third, the Digital Intelligence Index is constructed using multi-scale PCA.

Accurately measuring the quality of innovation output is a well-recognized challenge in the literature. The knowledge breadth method proposed by Zhang and Zheng [47] offers a novel approach by positing that patents with broader knowledge coverage span more technological domains and rely on more complex underlying knowledge. This complexity increases the difficulty of replication or incremental improvement, thereby indicating higher patent quality.

The patent database of the China National Intellectual Property Administration offers IPC codes for enterprise patents, with each IPC code corresponding to specific technological fields. For invention patents and utility model patents, IPC codes generally follow the structure “Section–Class–Sub-class–Main Group–Sub-group”, with the eight main sections denoted by uppercase letters A through H. For example, in the IPC code *A01B1/00*, “A01B” identifies the class (hand tools used in agriculture or forestry), and “1/00” designates the specific group, with the main and subgroups separated by a “/”.

However, simply counting the number of IPC classification codes is insufficient for evaluating a firm’s innovation level and may produce misleading results. For example, one patent might have two IPC codes (*A01B01/00* and *A01B01/10*), both within the same main group (*A01B01*), whereas another patent might have *A01B01/00* and *A01B02/10*, covering two distinct main groups (*A01B01* and *A01B02*). While both patents have the same number of codes, the latter covers a broader technological scope, reflecting greater knowledge breadth and higher quality.

To quantify the knowledge breadth based on industry concentration, this study utilizes the differences in IPC classification codes at the main group level. The calculation method is as follows:


$${patent\_knowdge}_{nt,type}=\text{1}-\sum \alpha^{\text{2}}$$ (7)

Because technological and market conditions vary significantly across industries, we adopt the adjustment method of Cao et al. [48] to account for industry heterogeneity:


$$\text{IG}=\frac{\text{1}-\sum \alpha^{\text{2}}}{{\text{X}}_{\text{i}}}$$ (8)

Here, *X*_*i*_ represents the annual average knowledge breadth of the industry to which firm *i* belongs. Industry averages are calculated using two-digit GICS codes published by the National Bureau of Statistics. Given that median industry knowledge breadth often contains numerous zero values, we use the mean industry knowledge breadth as a proxy for industry-level innovation quality.

Because the IPC codes for design patents differ substantially in structure from those for invention and utility model patents, the knowledge breadth method is unsuitable for assessing design patents. Therefore, the analysis of innovation quality is restricted to invention and utility model patents.

For the construction of the Digital Intelligence Index, five discrete variables are extracted from annual reports, representing the frequency of keywords related to cloud computing, big data, blockchain, artificial intelligence, and digital technology applications. The following steps are performed before calculating the index:

1. Standardization–All indicators are standardized to eliminate the effects of differing measurement units.
2. Correlation Testing–The Kaiser–Meyer–Olkin (KMO) measure and Bartlett’s test of sphericity are applied to assess the suitability of the data for factor analysis. A KMO value below 0.6 indicates unsuitability. In this study, the KMO value is 0.721 with a significance level of 0.000, confirming the appropriateness of factor analysis.
3. Correlation Matrix Calculation–Following standardization, a multi-segment correlation coefficient matrix is calculated and analyzed using the maximum likelihood estimation method.
4. Eigenvalue and Eigenvector Calculation – Eigenvalues and unit eigenvectors are computed from the correlation matrix to identify the underlying structure.
5. Determination of Principal Components – Following Lin and Du [50], the number of principal components is determined by retaining those with eigenvalues ≥ 1 (approach one) and ensuring a cumulative variance contribution of at least 85% (approach two). The first approach is used to determine the number of components, while the second is applied in constructing the index.

The results show that the first four principal components account for 90% of the total variance, effectively representing the five original explanatory variables. The Digital Intelligence Index is then constructed based on the cumulative variance contributions of these components.

## 5. Results and discussion

### 5.1. Descriptive statistics

All empirical analyses were conducted in Stata 18. Table 2 provides the descriptive statistics of the variables employed in this study. The dependent variable innovation efficiency (IE) ranges from 0.001 to 1.000, with a mean of 0.111 and a standard deviation of 0.118, indicating substantial variation in innovation performance across the sample enterprises. Innovation quality (IG) ranges from –0.211 to 7.069, with a mean of 1.474 and a standard deviation of 1.268, suggesting notable differences in patent knowledge breadth among SRDI firms.

**Table 2:** Descriptive statistics of variables.

Variable name	Variable symbol	Observations	Mean	St. Dev	Min	Max
Innovation Efficiency	*IE*	9990	0.111	0.118	0.001	1.000
Innovation Quality	*IG*	9990	1.474	1.268	0.000	7.069
Digital Intelligence Index	*Dig*	9990	−0.079	0.314	−0.211	3.258
Cloud Computing Level	*Cloud*	9990	0.482	0.969	0.000	5.403
Artificial Intelligence Level	*AI*	9990	0.318	0.765	0.000	5.263
Big Data Level	*Big Date*	9990	0.417	0.874	0.000	5.545
Blockchain Level	*Block*	9990	0.057	0.296	0.000	4.673
Digital Technology Application Level	*DT*	9990	0.610	1.012	0.000	6.073
Proportion of Largest Shareholder	*Top1*	9990	0.347	0.153	0.008	0.849
Number of Board Members	*Board*	9990	6.988	2.561	0.000	13.000
Industry Competition Level	*HHI*	9990	0.121	0.135	0.023	1.000
Asset Turnover Ratio	*TAT*	9990	0.007	0.004	0.000	0.124
Operating Cycle	*Cycle*	9990	0.053	0.006	0.008	0.132
Operating Cash Flow	*OCF*	9990	0.202	0.011	0.135	0.262
Return on Assets	*ROA*	9990	0.092	0.100	−1.921	1.409

Regarding digital intelligence, the mean value of digital intelligence level (*Dig*) is −0.079, with a maximum of 3.258 and a minimum of −0.211. This distribution shows that only a small proportion of firms have high digital intelligence levels, while the standard deviation of 0.314 reflects considerable heterogeneity across enterprises.

For the five component technologies, cloud computing, artificial intelligence, big data, blockchain, and digital technology applications, the maximum values are 5.403, 5.263, 5.545, 4.673, and 6.073, respectively, while the corresponding mean values are 0.482, 0.318, 0.417, 0.057, and 0.610. These results indicate that many SRDI enterprises have yet to adopt these digital technologies, with blockchain showing the lowest penetration. Except for blockchain, the standard deviations for the other technologies exceed 0.5, highlighting substantial disparities in adoption levels across firms.

### 5.2. Baseline regression

A fixed effects model is used to estimate the baseline regressions, examining the effect of digital intelligence on both the innovation efficiency and innovation quality of SRDI enterprises. The results are reported in Table 3 with columns (1) to (6) presenting the estimated for innovation efficiency and columns (7) to (12) showing the results for innovation quality.

**Table 3:** Regression results of the influence of digital intelligence technology on innovation performance.

Variable	(1) *IE*	(2) *IE*	(3) *IE*	(4) *IE*	(5) *IE*	(6) *IE*	(7) *IG*	(8) *IG*	(9) *IG*	(10) *IG*	(11) *IG*	(12) *IG*
*Dig*	0.038^***^ (9.56)						0.125^***^ (2.83)					
*Cloud*		0.015^***^ (11.85)						0.074^***^ (5.12)				
*AI*			0.012^***^ (7.72)						0.059^***^ (3.29)			
*Big Date*				0.010^***^ (7.37)						0.020 (1.24)		
*Block*					0.012^***^ (3.34)						0.040 (0.97)	
*DT*						0.013^***^ (10.54)						0.048^***^ (3.54))
*Constant*	−0.673^***^ (−20.20)	−0.669^***^ (−20.22)	−0.701^***^ (−21.18)	−0.695^***^ (−20.93)	−0.730^***^ (−22.20)	−0.667^***^ (−20.01)	−4.867^***^ (−13.02)	−4.748^***^ (−12.78)	−4.903^***^ (−13.25)	−4.999^***^ (−13.44)	−5.055^***^ (−13.76)	−4.813^***^ (−12.87)
Controls	Yes	Yes	Yes	Yes	Yes	Yes	Yes	Yes	Yes	Yes	Yes	Yes
Industry	Yes	Yes	Yes	Yes	Yes	Yes	Yes	Yes	Yes	Yes	Yes	Yes
Year	Yes	Yes	Yes	Yes	Yes	Yes	Yes	Yes	Yes	Yes	Yes	Yes
Observations	9990	9990	9990	9990	9990	9990	9990	9990	9990	9990	9990	9990
R^2^	0.277	0.280	0.275	0.274	0.271	0.278	0.208	0.210	0.209	0.208	0.208	0.209

Note: t-statistics in parentheses.

* p < 0.1.

** p < 0.05.

*** p < 0.01.

When the dependent variable is innovation efficiency, the coefficient for the core explanatory variable—digital intelligence level—is 0.038, statistically significant at the 1% level. This indicates that a 1% increase in the digital intelligence level is associated with an approximate 0.038% improvement in innovation efficiency. Furthermore, all five dimensions, cloud computing, artificial intelligence, big data, blockchain, and digital technology applications, are positively correlated with innovation efficiency at the 1% significance level. These results underscore the importance of digitalization, data utilization, and intelligent technologies in enhancing the innovation efficiency of SRDI enterprises. When the dependent variable is innovation quality, the coefficient for the digital intelligence index is 0.125, also statistically significant at the 1% level, indicating a 1% increase in the digital intelligence index corresponds to an approximate 0.125% improvement in innovation quality. At the dimensional level, cloud computing, artificial intelligence, and digital technology applications exhibit positive and significant correlations with innovation quality at the 1% level, whereas big data and blockchain do not. This suggests that, at present, the main drivers of higher innovation quality among SRDI enterprises are the adoption of cloud computing, artificial intelligence, and digital technology applications.

These findings align with global trends in digital transformation, where the adoption of digital intelligence technologies is increasingly recognized as a critical driver of innovation. In advanced economies such as the United States, Germany, and Japan, SMEs have rapidly adopted integrated digital technologies to enhance innovation, supported by national initiatives like Germany’s *Industry 4.0* and Japan’s *Society 5.0.* In China, the uptake of artificial intelligence and other data-driven technologies has accelerated innovation in specialized enterprises. For example, recent advancements such as DeepSeek’s R1 model demonstrate how algorithmic optimization can reduce reliance on expensive high-performance chips, thereby lowering infrastructure barriers. Compared with datafication and digitization, intelligent technologies such as AI and machine learning offer distinctive innovation advantages by enabling predictive analytics, adaptive decision-making, and automated optimization, allowing firms to respond more dynamically to market changes.

In summary, the results highlight that in resource-constrained environments, SRDI enterprises should strategically prioritize the adoption of digital intelligence tools, especially cloud computing, artificial intelligence, and digital technology applications, as these exert the strongest positive effects on both innovation efficiency and innovation quality.

### 5.3. Mediating effect analysis

Corporate innovation is a long-term process characterized by uncertainty and a high risk of failure in R&D factors that often complicate access to financial market support. For SRDI enterprises, R&D activities are particularly complex and risky, spanning multiple disciplines and industries, and require substantial financial resources. According to the theory of information asymmetry, there exists a structural information gap between external investors and enterprises. To reduce adverse selection and moral hazard, investors often raise financing costs or impose credit rationing, extending funding only to select applicants. As a result, many firms face financing constraints that compel them to scale back innovation activities to reduce financial pressure. Such constraints can also encourage enterprises to prioritize conservative innovation projects with short payback periods, while neglecting high-potential projects that require longer investment horizons. This short-term orientation weakens long-term competitiveness. Investors generally prefer to support firms with robust, long-term strategic plans; however, financing constraints can undermine such strategies, reducing investor willingness and increasing the risk of funding disruptions, which may lead to inefficient innovation investments.

The literature consistently finds that financing constraints hinder corporate innovation performance [51,52]. For example, Zhou et al. [53] and Fang and Hu [54] empirically demonstrate that alleviating financing constraints significantly improves innovation outcomes. revealing that alleviating these constraints significantly improves innovation outcomes. In this study’s context, it is reasonable to hypothesize that financing constraints mediate the relationship between digital intelligence and the innovation performance of SRDI enterprises. This hypothesis is tested using a two-way fixed effects model controlling for both industry and year effects. The regression results, reported in Table 4, show that the coefficient of the financing constraint SA index with respect to digital intelligence is –0.042, statistically significant at the 1% level. This indicates that higher levels of digital intelligence reduce the financing constraints faced by SRDI enterprises, thereby enhancing innovation performance.

**Table 4:** Results of mediating effect testing.

Variable	(1) *IE*	(2) *IG*	(3) *SA*
*Dig*	0.030^***^ (7.76)	0.086^**^ (2.49)	−0.042^***^ (−4.98)
_Cons	−0.590^***^ (−20.12)	−4.396^***^ (−16.82)	−0.015 (−0.28)
Controls	Yes	Yes	Yes
Industry	Yes	Yes	Yes
Year	Yes	Yes	Yes
Observations	9990	9990	9990
R^2^	0.289	0.211	0.608

Note: t-statistics in parentheses.

* p < 0.1.

** p < 0.05.

*** p < 0.01.

Beyond the empirical results, practical developments further illustrate the role of digital intelligence in overcoming financing barriers. According to the 2023 report from the China Banking and Insurance Regulatory Commission (CBIRC), financing demand among Chinese SMEs exceeds 13 trillion RMB, with a substantial portion unmet by traditional banks. To address this gap, digital lending platforms such as Ant Group and JD Digits have developed innovative financing models. By leveraging big data and artificial intelligence, these platforms assess real-time business performance to offer faster, more flexible loans, bypassing traditional credit evaluations. This approach enables SMEs to secure capital more efficiently and invest in innovation, mitigating longstanding financing constraints. Such experiences suggest that the adoption of digital finance and analytics platforms could also help SMEs in other countries address similar funding barriers, thereby fostering sustainable innovation.

### 5.4. Robustness test

To assess the robustness of the baseline regression, core variables were redefined and re-estimated. Digital intelligence levels were measured using the cumulative frequency of relevant terms in annual reports. Innovation efficiency was redefined as the ratio of R&D expenditure to operating revenue, and innovation quality as the natural logarithm of one plus the number of authorized patents. The re-estimation results remain consistent with the baseline findings, reinforcing the validity of the study’s conclusions.

Given the lengthy cycle from patent development to authorization, it is likely that the impact of digital intelligence on innovation performance occur with a lag effect. To test for this, the dependent variables, innovation efficiency and innovation quality, were regressed with one- to four-period lags of digital intelligence. The results show that the lagged coefficients remain significant, indicating a sustained positive effect of digital intelligence on innovation performance over time.

To address potential bidirectional causality and omitted variable bias, we employ the instrumental variable method. The mean frequency of digital intelligence–related terminology in the annual reports of peer companies is used as the instrumental variable. A two-stage least squares (2SLS) estimation is applied to control for endogeneity.

Table 5 reports the second-stage regression results. The Kleibergen-Paap LM statistic is significant at the 1% level, rejecting the null hypothesis of insufficient identification. The Cragg-Donald Wald F statistic is 238.261, exceeding conventional thresholds and rejecting the weak instrument hypothesis. These diagnostics confirm that the chosen instrumental variable is both valid and reliable. As indicated in Table 5, the coefficient for the digital intelligence level (Dig) remains significantly positive, demonstrating that the relationship between digital intelligence and innovation performance persists even after addressing endogeneity concerns.

**Table 5:** Results of instrumental variable testing.

Variable	(1) Second-Stage *IE*	(2) Second-Stage *IG*
*Dig*	0.070^***^ (3.20)	2.291^***^ (8.38)
*Constant*	−0.619^***^ (−12.56)	−1.228^**^ (−2.00)
Kleibergen-Paap LM Statistic	324.014	324.014
Cragg-Donald Wald F Statistic	332.630 (16.38)	332.630 (16.38)
Controls	Yes	Yes
Industry	Yes	Yes
Year	Yes	Yes
Observations	9990	9990
R^2^	0.272	0.018

Note: t-statistics in parentheses.

* p < 0.1.

** p < 0.05.

*** p < 0.01.

The Cragg-Donald Wald F Statistic exceeds the Stock-Yogo critical value for the weak instrument test at the 10% significance level.

Table 6 reports the results of the difference-in-differences (DiD) analysis. The regression coefficient of the primary explanatory variable (Dig_Shock) is significantly positive at least at the 10% level, indicating a robust and positive effect. This result further supports the study’s findings, demonstrating that using the timing of digital intelligence terminology in annual reports as the policy impact point provides an effective strategy for addressing the causal identification challenges inherent in this research.

**Table 6:** Results of the difference-in-differences analysis.

Variable	(1) *IE*	(2) *IG*	(3) *IE*	(4) *IG*
*Dig_Shock*	0.045^***^ (18.38)	0.254^***^ (9.68)	0.023^***^ (8.64)	0.049^*^ (1.68)
*Constant*	0.018 (1.02)	−0.302 (−1.55)	−0.672^***^ (−20.02)	−4.935^***^ (−13.12)
Controls	No	No	Yes	Yes
Industry	Yes	Yes	Yes	Yes
Year	Yes	Yes	Yes	Yes
Observations	9990	9990	9990	9990
R^2^	0.192	0.176	0.276	0.208

Note: t-statistics in parentheses.

* p < 0.1.

** p < 0.05.

*** p < 0.01.

**Fig 2:**
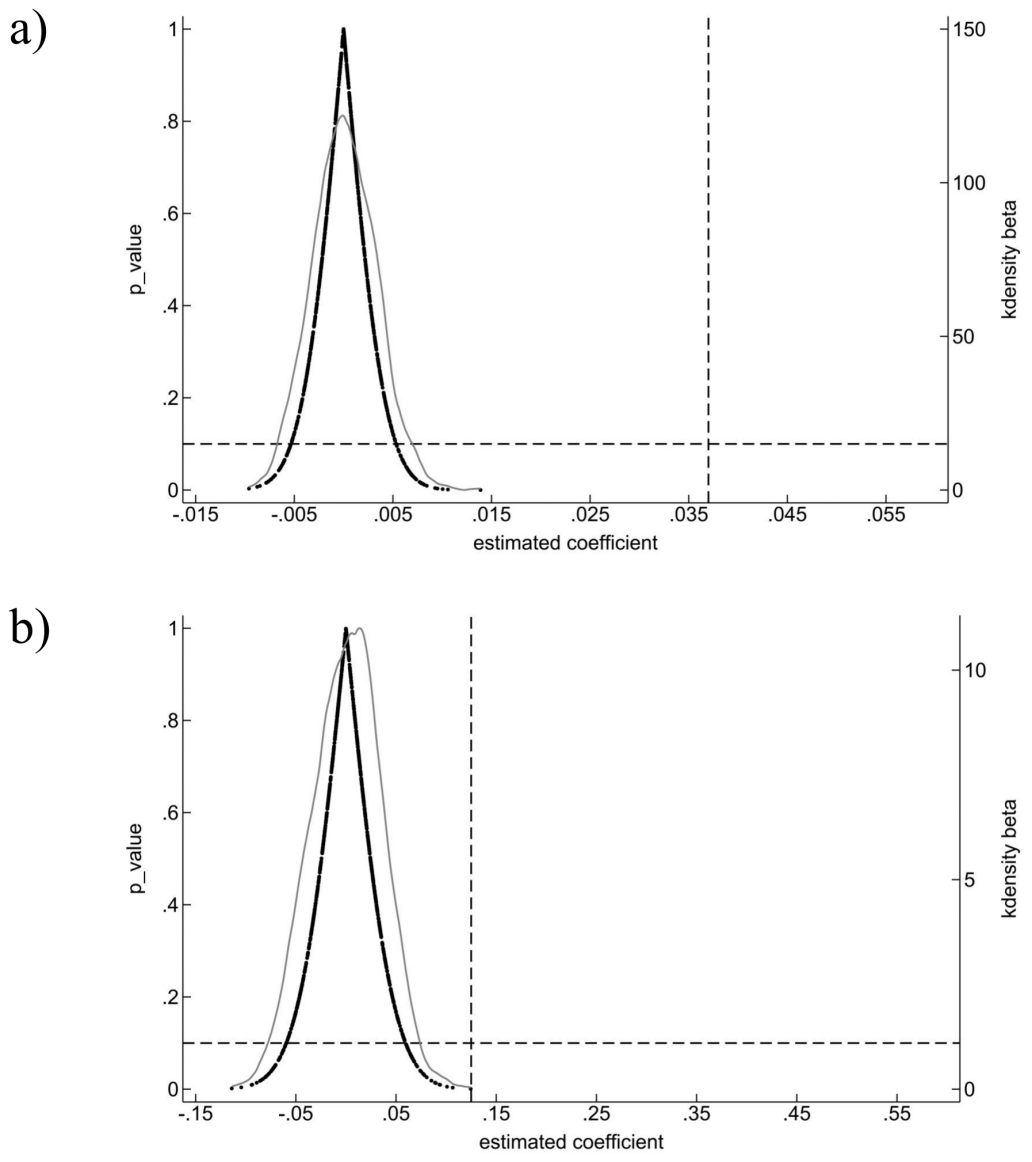
Placebo test. a) Placebo test for innovation efficiency b) Placebo test for innovation quality.

To examine whether the impact of digital intelligence levels on the innovation performance of SRDI enterprises could be driven by random factors, a placebo test is conducted. Following the approach of Liu [55], 1,000 are generated to construct a “pseudo-policy dummy variable” based on the distribution of the digital intelligence levels in the baseline regression. Regression estimates are then obtained using Models (3) and (4), with the results shown in Fig 2. The findings reveal that the mean regression coefficients for both innovation efficiency and innovation quality, when associated with the pseudo-policy dummy variable, are close to zero and smaller than those from the baseline regression. In addition, the distribution of the estimated coefficients approximates normality, with p-values exceeding 0.10. This indicates that, at the 10% significance level, the placebo results are statistically insignificant. These results suggest that the estimated effects of digital intelligence levels on innovation performance are not driven by random factors, thereby providing further support for the robustness of this study’s conclusions.

### 5.5. Heterogeneity analysis

To examine whether the impact of digital intelligence on the innovation performance of SRDI enterprises varies by ownership structure, a grouped regression analysis is conducted for state-owned and non-state-owned enterprises. The results show that digital intelligence has no significant effect on the overall innovation performance of state-owned enterprises in China, whereas it has a clear and positive effect on non-state-owned enterprises—particularly in enhancing innovation quality. Accordingly, Table 7 reports only the results for non-state-owned enterprises. For state-owned enterprises, no significant relationship is found between overall digital intelligence levels and innovation performance. However, the adoption of artificial intelligence and digital technology applications has a significant positive effect on innovation efficiency.

**Table 7:** Analysis of innovation performance in non-state-owned enterprises.

Variable	Innovation Efficiency						Innovation Quality					
	(1) *IE*	(2) *IE*	(3) *IE*	(4) *IE*	(5) *IE*	(6) *IE*	(7) *IG*	(8) *IG*	(9) *IG*	(10) *IG*	(11) *IG*	(12) *IG*
*Dig*	0.050^***^ (11.26)						0.245^***^ (4.95)					
*Cloud*		0.017^***^ (12.92)						0.077^***^ (5.02)				
*AI*			0.014^***^ (7.96)						0.089^***^ (4.53)			
*Big Date*				0.012^***^ (7.83)						0.036^**^ (2.08)		
*Block*					0.022^***^ (5.27)						0.185^***^ (3.90)	
*DT*						0.013^***^ (10.22)						0.054^***^ (3.73)
*Constant*	−0.594^***^ (−16.38)	−0.598^***^ (−16.64)	−0.638^***^ (−17.72)	−0.631^***^ (−17.46)	−0.671^***^ (−18.79)	−0.609^***^ (−16.83)	−4.925^***^ (−12.06)	−4.992^***^ (−12.31)	−5.074^***^ (−12.57)	−5.211^***^ (−12.84)	−5.260^***^ (−13.16)	−5.061^***^ (−12.43)
*Controls*	Yes	Yes	Yes	Yes	Yes	Yes	Yes	Yes	Yes	Yes	Yes	Yes
Industry	Yes	Yes	Yes	Yes	Yes	Yes	Yes	Yes	Yes	Yes	Yes	Yes
Year	Yes	Yes	Yes	Yes	Yes	Yes	Yes	Yes	Yes	Yes	Yes	Yes
Observations	8766	8766	8766	8766	8766	8766	8766	8766	8766	8766	8766	8766
R^2^	0.278	0.282	0.273	0.273	0.270	0.276	0.224	0.224	0.224	0.223	0.224	0.223

Note: t-statistics in parentheses.

* p < 0.1.

** p < 0.05.

*** p < 0.01.

In contrast, the results for non-state-owned enterprises indicate that cloud computing, artificial intelligence, big data, and blockchain all significantly improve innovation efficiency. Moreover, artificial intelligence, cloud computing, blockchain, and big data are positively associated with innovation quality at varying levels of statistical significance. These findings suggest that non-state-owned enterprises primarily enhance innovation quality through the adoption of digitalization and intelligence-related technologies. Further analysis indicates that non-state-owned enterprises benefit more from digital technologies due to their operation in highly competitive and dynamic market environments. Faced with continuous pressure to innovate and maintain competitiveness, these firms are more likely to adopt advanced technologies, such as artificial intelligence, big data, and blockchain, rapidly and extensively. By contrast, state-owned enterprises tend to derive fewer benefits from digital technologies. Their focus on economic stability and alignment with national policy, combined with institutional inertia and bureaucratic decision-making processes, slows the adoption of new technologies and reduces the capacity to leverage digitalization for innovation.

The empirical analysis also finds that digital intelligence can have a suppressive effect on the innovation quality of state-owned enterprises, due to blockchain technology. This study posits that the conservative organizational cultures and slower decision-making processes in state-owned enterprises, combined with the transparency and decentralization inherent in blockchain systems, can disrupt established management structures. These disruptions may increase internal friction and adaptation costs, thereby hindering innovation quality.

To assess whether the impact of digital intelligence levels on the innovation performance of SRDI enterprises differs across regions, the sample is divided into two groups, Eastern China and Central–Western China, based on economic development levels and geographical location. Separate regression analyses are conducted for each group.

Table 8 presents the results. For the Eastern sample, the regression coefficients of the digital intelligence index for innovation efficiency and innovation quality are 0.022 and 0.117, respectively, both significant at the 1% level. In the Central-Western sample, the coefficient for innovation efficiency is 0.016, significant at the 10% level, but the relationship with innovation quality is not statistically significant. These findings indicate that digital intelligence exerts a stronger positive influence on innovation performance in Eastern enterprises, with the effect primarily driven by improvements in innovation quality.

**Table 8:** Analysis of innovation efficiency in the eastern region.

Variable	Innovation Efficiency						Innovation Quality					
	(1) *IE*	(2) *IE*	(3) *IE*	(4) *IE*	(5) *IE*	(6) *IE*	(7) *IG*	(8) *IG*	(9) *IG*	(10) *IG*	(11) *IG*	(12) *IG*
*Dig*	0.038^***^ (8.56)						0.115^**^ (2.31)					
*Cloud*		0.017^***^ (11.41)						0.082^***^ (5.01)				
*AI*			0.010^***^ (5.89)						0.059^***^ (2.99)			
*Big Date*				0.011^***^ (6.72)						0.015 (0.82)		
*Block*					0.011^***^ (2.66)						0.004 (0.10)	
*DT*						0.013^***^ (10.13)						0.052^***^ (3.47)
*Constant*	−0.648^***^ (−13.93)	−0.639^***^ (−13.84)	−0.682^***^ (−14.73)	−0.672^***^ (−14.46)	−0.709^***^ (−15.36)	−0.631^***^ (−13.56)	−5.316^***^ (−10.18)	−5.144^***^ (−9.90)	−5.333^***^ (−10.29)	−5.459^***^ (−10.50)	−5.516^***^ (−10.70)	−5.195^***^ (−9.94)
Controls	Yes	Yes	Yes	Yes	Yes	Yes	Yes	Yes	Yes	Yes	Yes	Yes
Industry	Yes	Yes	Yes	Yes	Yes	Yes	Yes	Yes	Yes	Yes	Yes	Yes
Year	Yes	Yes	Yes	Yes	Yes	Yes	Yes	Yes	Yes	Yes	Yes	Yes
Observations	8109	8109	8109	8109	8109	8109	8109	8109	8109	8109	8109	8109
R^2^	0.294	0.299	0.291	0.292	0.288	0.297	0.205	0.207	0.205	0.204	0.204	0.205

Note: t-statistics in parentheses.

* p < 0.1.

** p < 0.05.

*** p < 0.01.

In particular, in the Eastern region, the adoption of digitalization, digitization, and intelligent technologies significantly enhances innovation efficiency, with cloud computing and artificial intelligence playing especially prominent roles in improving innovation quality. In the Central–Western region, digital intelligence also positively influences innovation efficiency, but with lower statistical significance than in the East. Although the overall effect on innovation quality in the Central–Western sample is insignificant, the regression coefficient for artificial intelligence related to innovation quality is 0.059, higher than that in the Eastern region. This suggests that, while regional disparities exist in the overall impact of digital intelligence, targeted investment in artificial intelligence technologies could yield substantial gains in innovation quality for SMEs in less developed regions.

Industries face distinct market challenges and opportunities, resulting in different motivations and pathways for leveraging digital intelligence technologies to enhance efficiency. As a pillar of the Chinese economy, the manufacturing sector plays a crucial role in promoting high-quality development and enhancing competitiveness. Accordingly, SRDI enterprises are concentrated in manufacturing.

To capture industry-specific effects, the sample is divided into manufacturing and non-manufacturing enterprises. Table 9 presents the results for the manufacturing sector. For manufacturing enterprises, the digital intelligence index is significantly and positively correlated with both innovation efficiency and innovation quality. By contrast, in non-manufacturing enterprises, the digital intelligence index is significantly associated with innovation efficiency but shows no significant relationship with innovation quality. These findings suggest that manufacturing enterprises’ innovation performance is driven by improvements in both efficiency and quality, whereas non-manufacturing enterprises primarily benefit through efficiency gains.

**Table 9:** Analysis of innovation performance in the manufacturing sector.

Variable	Innovation Efficiency						Innovation Quality					
	(1) *IE*	(2) *IE*	(3) *IE*	(4) *IE*	(5) *IE*	(6) *IE*	(7) *IG*	(8) *IG*	(9) *IG*	(10) *IG*	(11) *IG*	(12) *IG*
*Dig*	0.060^***^ (10.78)						0.319^***^ (5.28)					
*Cloud*		0.019^***^ (12.26)						0.110^***^ (6.63)				
*AI*			0.016^***^ (8.22)						0.081^***^ (3.81)			
*Big Date*				0.018^***^ (9.58)						0.082^***^ (4.02)		
*Block*					0.034^***^ (6.48)						0.168^**^ (2.91)	
*DT*						0.012^***^ (8.32)						0.065^***^ (4.24)
*Constant*	−0.580^***^ (−16.24)	−0.579^***^ (−16.29)	−0.608^***^ (−17.07)	−0.597^***^ (−16.77)	−0.630^***^ (−17.76)	−0.595^***^ (−16.63)	−4.569^***^ (−11.72)	−4.529^***^ (−11.66)	−4.726^***^ (−12.19)	−4.695^***^ (−12.09)	−4.840^***^ (−12.55)	−4.641^***^ (−11.90)
Controls	Yes	Yes	Yes	Yes	Yes	Yes	Yes	Yes	Yes	Yes	Yes	Yes
Industry	Yes	Yes	Yes	Yes	Yes	Yes	Yes	Yes	Yes	Yes	Yes	Yes
Year	Yes	Yes	Yes	Yes	Yes	Yes	Yes	Yes	Yes	Yes	Yes	Yes
Observations	8613	8613	8613	8613	8613	8613	8613	8613	8613	8613	8613	8613
R^2^	0.249	0.252	0.245	0.247	0.242	0.245	0.192	0.194	0.191	0.191	0.190	0.191

Note: t-statistics in parentheses.

* p < 0.1.

** p < 0.05.

*** p < 0.01.

Within manufacturing enterprises, cloud computing, artificial intelligence, and big data significantly enhance both innovation efficiency and innovation quality. The application of digital technologies exerts a pronounced positive impact on innovation quality, whereas the influence of blockchain on innovation quality remains comparatively less significant. In non-manufacturing enterprises, although the level of digital intelligence significantly promotes innovation efficiency, it simultaneously suppresses the development of innovation quality, with blockchain technology identified as the primary inhibiting factor.

The underlying reason may lie in blockchain’s reliance on robust the data-sharing and trust mechanisms among multiple stakeholders. In non-manufacturing sectors, where customer demands are diverse and services are highly personalized, such data-sharing is more complex, increasing implementation challenges and diminishing blockchain’s contribution to innovation quality. These results highlight the importance of adopting targeted digital strategies. For non-manufacturing SMEs in particular, aligning technological adoption with operational models is critical to maximizing innovation benefits while avoiding unintended negative effects.

## 6. Conclusion

This study investigates how digital intelligence technologies influence the innovation performance of SRDI enterprises in China. Using data from specialized and innovative listed companies in China from 2014 to 2022, it measures digital intelligence across three dimensions, datafication, digitalization, and intelligentization, and evaluates innovation performance through innovation efficiency and innovation quality. Together, these measures enable an assessment of the extent to which digital intelligence technologies shape innovation outcomes.

The findings yield several important conclusions: First, digital intelligence technologies significantly improve both innovation efficiency and innovation quality in China’s SRDI enterprises, with this positive effects persisting for up to four years. This impact is particularly evident in innovation efficiency, driven primarily by cloud computing and artificial intelligence. Second, digital intelligence plays a crucial role in alleviating the financing constraints, thereby reducing risks in the financing process, improving capital allocation, and strengthening firms’ innovation capacity and market competitiveness. Third, the positive influence of digital intelligence on innovation is most pronounced in non-state-owned enterprises, firms in the Eastern region, and the manufacturing sector. By contrast, the enhancement of innovation efficiency is relatively stronger in state-owned enterprises, firms in the Central-Western region, and non-manufacturing SRDI enterprises. Notably, blockchain technology exhibits a suppressive effect on innovation quality in state-owned enterprises and the non-manufacturing sector. Overall, SMEs can foster sustainable growth by prioritizing the adoption of readily deployable digital intelligence technologies, particularly cloud computing and artificial intelligence, while implementing big data and blockchain solutions selectively, based on specific developmental needs.

This study proposes the following recommendations: First, SMEs should accelerate the adoption of cloud computing and AI to advance digital transformation, improve decision-making, and foster innovation. Cloud platforms provide scalable data processing capabilities without the need for substantial infrastructure investments, making them especially valuable for SMEs in developing economies where capital resources are limited. Artificial intelligence tools, such as ChatGPT, can improve customer service, optimize internal operations, and reduce costs. In developing countries, these technologies can help SMEs adapt to rapidly changing market conditions, increase productivity, and strengthen competitiveness in both domestic and global markets. Second, SMEs should leverage blockchain and big data risk analytics to improve the financing conditions. These technologies optimize capital allocation, increase transparency, and reduce financing risks. Big data analytics helps SMEs present their financial conditions more clearly, improving transparency with financial institutions, while blockchain reduces information asymmetry and strengthens creditworthiness. In China, where SMEs often face restricted access to capital, these technologies offer practical tools for achieving long-term financial stability. Finally, SMEs should utilize digital technologies to support internationalization and strengthen global competitiveness. By enabling operations beyond geographical boundaries, particularly through cross-border e-commerce platforms, these technologies facilitate access to international customers. Chinese SMEs, in particular, should incorporate such tools into their global expansion strategies to enhance market entry and positioning in the international arena.

## 7. Limitations

The primary limitation of this study lies in its exclusive focus on SRDI enterprises in China, which may constrain the generalizability of the findings to other types of enterprises or different regional contexts. Future research could address this by extending the analysis to comparable enterprises in other countries, such as Germany’s “hidden champions” or niche firms in the United States and Japan. Such cross-country comparisons would enable a deeper understanding of how varying institutional frameworks, market structures, and economic environments shape the relationship between digital intelligence and innovation.

Additionally, this research is limited to the period from 2014 to 2022 due to the unavailability of prior data, which represents a significant limitation. During this period, SRDI enterprises faced multiple external challenges. China’s supply-side structural reforms increased raw material costs in sectors such as manufacturing, intensifying financial pressure on SMEs. The COVID-19 pandemic further disrupted supply chains, with semiconductor shortages delaying product development for technology-driven firms. Although the 2022 VAT rebate policy provided some financial relief, uneven implementation restricted access for certain non-state-owned enterprises. These factors may have limited the ability of SMEs to fully leverage digital intelligence for innovation. Future research could examine the long-term effects of digital intelligence technologies on innovation performance, including whether these impacts are sustainable, how digital adoption reshapes organizational dynamics, and what new challenges or opportunities emerge as enterprises deepen their digital transformation.

Another limitation of this study lies in its focus on a limited set of determinants of innovation performance, as it was not feasible to incorporate all potentially relevant factors into the model. Future research should expand the scope to include additional drivers, such as organizational culture, management practices, and external collaborations, to provide a more comprehensive understanding of the factors influencing innovation performance.

## Supporting information

S1 Appendix Keyword dictionary for the digital intelligence index. (PDF)

S2 Appendix Construction of the digital intelligence index from annual report keywords. (PDF)

S3 Appendix Construction of the “knowledge breadth” metric from IPC data. (PDF)

S4 Appendix Variable—Level data source summary. (PDF)

S5 Appendix Three-stage DEA computation steps. (PDF)

S6 Appendix GICS sector classification. (PDF)

S7 Appendix SRDI enterprises list. (XLSX)
