# Correction: The impact of digital intelligence technologies on innovation performance: Evidence from specialized, refined, differential and innovative enterprises

**DOI:** 10.1371/journal.pone.0344109

**Published:** 2026-03-02

**Authors:** Tian Zhao, Fa Zhang, Guoliang Zhuo, Qingqing Zhang, Qingwen Yuan

The email address for one of the corresponding authors is incorrect. Qingwen Yuan’s email address is: skd993630@sdust.edu.cn
