# Correction: The impact of digital intelligence technologies on innovation performance: Evidence from specialized, refined, differential and innovative enterprises

**DOI:** 10.1371/journal.pone.0345648

**Published:** 2026-03-23

**Authors:** Tian Zhao, Fa Zhang, Guoliang Zhuo, Qingqing Zhang, Qingwen Yuan

The Funding statement for this article is incorrect. The correct Funding statement is as follows:

1. National Social Science Fund of China (Grant No. 23BGL052) Authors: Tian Zhao Funder: National Social Science Fund of China Funder Website: http://www.nopss.gov.cn/
2. Natural Science Foundation of Shandong Province (Grant No. ZR2024MG016) Authors: Tian Zhao Funder: Natural Science Foundation of Shandong Province Funder Website: http://kjt.shandong.gov.cn/
3. Shandong Social Science Foundation General Project (Grant No. 22CJJJ32) Authors: Qingwen Yuan Funder: Shandong Social Science Foundation Funder Website: https://sdsk.sdxc.gov.cn/
4. Youth Key Project of Shandong Provincial Philosophy and Social Science Planning Fund (2025) Authors: Tian Zhao Funder: Shandong Provincial Philosophy and Social Science Planning Office Funder Website: https://www.sdskw.cn/

Role of the Funders: The sponsors/funders played an active role in the entire research process, including study design, data collection and analysis, decision to publish, and the preparation of the manuscript.
