# Supplementary material for: The impact of digital intelligence technologies on innovation performance: Evidence from specialized, refined, differential and innovative enterprises
Source: PLoS One. 2026 Feb 10;21(2):e0339567. doi: 10.1371/journal.pone.0339567 (PMC12890174; doi:10.1371/journal.pone.0339567)
Supplement: S1 Appendix — (PDF) [file pone.0339567.s001.pdf]

S1 Appendix. Keyword Dictionary for the Digital Intelligence Index

Construction of the digital-intelligence keyword dictionary. Building on five core foundational technology groups (cloud computing, big data, blockchain, artificial intelligence, and digital applications), we expanded and structured the dictionary by borrowing from authoritative policy documents and research reports. Specifically, we took as blueprints the Special Action Plan for Empowering SMEs via Digitalization, the Implementation Plan for “Cloud Adoption, Data Utilization, and Intelligence Empowerment”, the 2020 Digital Transformation Trends Report, and recent Government Work Reports. We then organized all entries into a two-layer taxonomy—Foundational Technology Use and Applied Practice/Implementation—and visualized the taxonomy as a feature map (Fig. 1). Group summaries with representative terms are provided in Table 1.

To ensure consistency and comparability, we applied the following inclusion/exclusion rules:

(1) Inclusion: expressions directly describing the firm’s digitalization/AI-enabled transformation (including bilingual synonyms and common abbreviations) that can be mapped to the two-layer taxonomy;

(2) Negation filter: occurrences preceded by explicit negation cues (e.g., “no/not/none/not yet” or their Chinese equivalents) within the same sentence/clause are excluded to avoid counting statements of absence;

(3) Entity scope: we restrict to statements about the focal firm, excluding mentions referring to shareholders, customers, suppliers/partners, or executive biographies;

The resulting dictionary underpins the extraction of digital-intelligence terms from annual reports and the construction of the keyword feature set (Table 1), which feeds into the PCA-based Digital Intelligence Index (S2 Appendix).

Table A1 Keyword Dictionary for the Digital Intelligence Index

**Big Data Technology**	**Cloud Computing Technology**	**Blockchain Technology**	**Digital Technology Application**
Big Data Technology	Cloud Computing Technology	Blockchain Technology	Digital Technology Application
Big Data	Cloud Computing	Blockchain	Mobile Internet
Data Mining	Stream Computing	Digital Currency	Industrial Internet
Text Mining	Graph Computing	Distributed Computing	Mobile Internet Connectivity
Data Visualization	In-Memory Computing	Differential Privacy Technology	Internet Healthcare
Heterogeneous Data	Multi-Party Secure Computation	Smart Financial Contracts	E-Commerce
Credit Investigation	Brain-like Computing		Mobile Payment
Augmented Reality	Green Computing		Third-Party Payment
Mixed Reality	Cognitive Computing		NFC Payment
Virtual Reality	Converged Architecture		Intelligent Energy
	Billion-level		B2B (Business-to-Business)

	Concurrency		
	EB-level Storage		B2C (Business-to-Consumer)
	Internet of Things (IoT)		C2B (Consumer-to-Business)
	Cyber-Physical Systems		C2C (Consumer-to-Consumer)
			O2O (Online-to-Offline)
			Networking
			Smart Wearables
			Smart Agriculture
			Intelligent Transportation
			Intelligent Healthcare
			Intelligent Customer Service
			Smart Home
			Intelligent Investment Advisory
			Intelligent Cultural Tourism
			Intelligent Environmental Protection
			Smart Grid
			Intelligent Marketing
			Digital Marketing
			Unmanned Retail
			Internet Finance
			Digital Finance
			Fintech
			Financial Technology
			Quantitative Finance
			Open Banking
