# Supplementary material for: The impact of digital intelligence technologies on innovation performance: Evidence from specialized, refined, differential and innovative enterprises
Source: PLoS One. 2026 Feb 10;21(2):e0339567. doi: 10.1371/journal.pone.0339567 (PMC12890174; doi:10.1371/journal.pone.0339567)
Supplement: S2 Appendix — (PDF) [file pone.0339567.s002.pdf]

S2 Appendix. Construction of the Digital Intelligence Index from Annual Report Keywords

Digital-intelligence keyword frequency.

Specifically, digital intelligence was measured by counting the occurrences of five core digital technology keywords (cloud computing, big data, blockchain, artificial intelligence, and digital technology applications) in the annual reports of all sampled firms. For example, if the report of Firm A contained “cloud computing” 10 times, “big data” 6 times, and “artificial intelligence” 4 times, these values were recorded as the raw digital intelligence scores for that firm, as shown in columns 3 to 7 of Table 1. The complete keyword dictionary is provided in **S1 Appendix**.

Composite index via PCA.

The construction of the digital intelligence index involved the following main steps:

(1)Standardization of the raw keyword frequencies;

(2)Correlation testing using the Kaiser-Meyer-Olkin (KMO) and Bartlett’s tests to confirm suitability for factor analysis (with a KMO value greater than 0.721);

(3)Factor analysis and calculation of eigenvalues and eigenvectors;

(4)Principal component selection based on eigenvalues and unit eigenvectors, as detailed in the main text;

(5) Construction of the composite index using the weighted principal components. The principal component loadings (contribution rates), denoted as c1, c2, c3, and c4, were then used to calculate the composite digital intelligence index as follows:

Ctotal =

（c1×variance contribution of PC1+c2×variance contribution of PC2+c3×variance contribution of PC3+c4×variance contribution of PC4）/cumulative variance contribution of PC4

For clarity, we have also included a summary workflow diagram as follows:

Annual Report Text → Keyword Extraction and Counting → Standardization → Factor Analysis → Index Construction.

Table 1 Digital Intelligence Index Data

code	year	Cloud	Big data	Blockchain	AI	digital technology	c1	c2	c3	c4	DiginteIndex	lnDiginteIndex
Firm A	2014	0	0	0	0	16	-0.07	-0.62	0.47	0.51	0.01	0.01
Firm A	2015	0	0	1	0	5	-0.29	-0.12	0.10	0.20	-0.11	-0.12
Firm A	2016	0	0	0	0	16	-0.07	-0.62	0.47	0.51	0.01	0.01
Firm A	2017	1	0	0	0	6	-0.27	-0.18	0.18	0.14	-0.11	-0.12
Firm A	2018	0	0	0	1	1	-0.27	0.40	0.27	0.17	0.02	0.02
Firm A	2019	0	0	0	0	2	-0.41	0.01	0.04	0.09	-0.17	-0.19
Firm A	2020	0	0	0	0	3	-0.39	-0.03	0.07	0.12	-0.16	-0.17
Firm A	2021	0	0	0	0	2	-0.41	0.01	0.04	0.09	-0.17	-0.19
Firm A	2022	0	1	0	0	2	-0.35	0.01	-0.03	0.11	-0.15	-0.17
Firm A	2023	0	2	0	0	2	-0.30	0.02	-0.09	0.12	-0.14	-0.15

Table 2   Results of the multi-grid principal component analysis

Principal Component	Initial Eigenvalue		
	Eigenvalue	Variance Contribution (%)	Cumulative Variance Contribution (%)
1	2.171	43.42	43.42
2	0.891	17.81	61.23
3	0.789	15.78	77.01
4	0.685	13.7	90.71
5	0.464	9.29	100

We hope this presentation enables readers to follow the full calculation process clearly, from text parsing and keyword counting through standardization and PCA based aggregation, and to reproduce the index independently.
