# Supplementary material for: The impact of digital intelligence technologies on innovation performance: Evidence from specialized, refined, differential and innovative enterprises
Source: PLoS One. 2026 Feb 10;21(2):e0339567. doi: 10.1371/journal.pone.0339567 (PMC12890174; doi:10.1371/journal.pone.0339567)
Supplement: S3 Appendix — (PDF) [file pone.0339567.s003.pdf]

S3 Appendix. Construction of the “Knowledge Breadth” Metric from IPC Data

Definition. Knowledge breadth captures the diversity of technological fields represented in a patent’s IPC classifications. Following Zhang & Zheng (full citation in the main text), we collapse IPC codes to the main-group level (i.e., the substring before “/”; e.g., H05B37/02 → H05B37). For a given patent i, let p_{ig} denote the share of its IPC codes that fall into main group g. We compute: KB_i = 1 − Σ_g (p_{ig})^2. This is the Gini–Simpson (1–HHI) diversity index. It equals 0 if all IPC codes lie in a single main group and increases as codes span more distinct groups more evenly. IPC fields are standard bibliographic items and can be retrieved from official portals—for example, China: the China National Intellectual Property Administration (CNIPA) patent search; United States: the United States Patent and Trademark Office (USPTO) Patent Public Search; global coverage: the European Patent Office (EPO) Espacenet and the World Intellectual Property Organization (WIPO) PATENTSCOPE; open aggregators such as The Lens or Google Patents also expose International Patent Classification fields. The patent data used in this study are sourced from the CNIPA patent database.

Worked examples (as in Table 1).

Patent 1 (Firm A): IPC codes H05B37/02, H05B37/02 → one main group (H05B37), proportions [1]. KB = 1 − 1^2 = 0.

Patent 2 (Firm A): G06F3/045, G06F3/044, G06F3/045 → one main group (G06F3), proportions [1]. KB = 1 − 1^2 = 0.

Patent 3 (Firm A): H02H7/26, H02H1/00, H02H7/26 → two main groups (H02H7, H02H1) with proportions [2/3, 1/3]. KB = 1 − [(2/3)^2 + (1/3)^2] = 0.4444.

Reproducible Stata implementation (patent-level KB)

Before running the code, researchers should first separate the long string of IPC classification numbers into individual IPC codes using Excel’s “Text to Columns” function, as illustrated in Columns 3–5 of Table 1.

To facilitate computation by interested researchers, we provide Stata code that can be used to calculate the metric directly; the code is given below:

```
* Import patent data
* Split IPC codes into separate variables
split ipc_codes, parse(" ") gen(ipc_code_)
* 1. Extract the main group part (before "/") for each IPC classification code
gen main_group_1 = substr(ipc_code_1, 1, strpos(ipc_code_1, "/") - 1)
...
gen main_group_n = substr(ipc_code_n, 1, strpos(ipc_code_n, "/") - 1)
* Generate HHI value for knowledge breadth
* Create a new variable `result` to store the calculation result for each row
gen result = 0
* Iterate over each row of data
forval row = 1/`=_N' {
    * Retrieve the quantity for the current row
```

```
        local quantity = quantity[`row']
        * Initialize result to 0
        local row_result = 0
        * Create a local macro to store unique values for the current row
        local unique_values ""
        * Iterate through each column (main_group_1 to main_group_n)
        foreach col of varlist main_group_1-main_group_n {
            * Retrieve the value in the current column
            local value = `col'[`row']
            * Determine whether the current value already exists in unique_values
            if "`value'" != "" & !strpos("`unique_values'", "`value'") {
                * If the value is not in unique_values, proceed with counting
                * Count the occurrences of the current value in this row
                local count = 0
                foreach col2 of varlist main_group_1-main_group_n {
                    if `col2'[`row'] == "`value'" {
                        local count = `count' + 1
                    }
                }
                * Calculate the square of the frequency of this value and add it to row_result
                local result_part = (`count' / `quantity')^2
                local row_result = `row_result' + `result_part'
                * Update unique_values to avoid double-counting
                local unique_values "`unique_values' `value'"
            }
        }
        * Store the calculation result for each row in the result variable
        replace result = `row_result' in `row'
    }
```

Notes：

IPC main group is operationalized as the substring before "/" (e.g., H05B37).

Duplicate IPC codes within a patent are allowed; counts determine p_{ig}.

Table 1 Example of Knowledge Breadth Calculation

code	IPC Code	IPC Code1	IPC Code2	IPC Code3	patent_knowledge
Firm A	H05B37/02<br>H05B37/02	H05B37/02	H05B37/02		0
Firm A	G06F3/045;G06F3/044<br>G06F3/045	G06F3/045	G06F3/044	G06F3/045	0
Firm A	H02H7/26;H02H1/00<br>H02H7/26	H02H7/26	H02H1/00	H02H7/26	0.4444
