# Supplementary material for: The impact of digital intelligence technologies on innovation performance: Evidence from specialized, refined, differential and innovative enterprises
Source: PLoS One. 2026 Feb 10;21(2):e0339567. doi: 10.1371/journal.pone.0339567 (PMC12890174; doi:10.1371/journal.pone.0339567)
Supplement: S4 Appendix — (PDF) [file pone.0339567.s004.pdf]

S4 Appendix. Variable–Level Data Source Summary

The variables below correspond to the four primary data sources described in Section 4 ("Method and Data") of the manuscript. This supplementary table is provided solely to enhance transparency; all sources were already specified in the main text. All underlying data are accessible: public sources are open, and licensed financial data (CSMAR, iFinD) are available via subscription.

Table 1 Variable Data Source Summary

Variable Name	Source Database / Category	Description	Coverage(Years)	Notes
Innovation Efficiency	(1) Financial Data – CSMAR; iFinD (inputs/outputs/env)	Innovation efficiency — Efficiency is evaluated using a three-stage data envelopment analysis. The outputs are patent counts and operating revenue; the inputs are R&D expenditure and R&D personnel; the environmental variables are firm age (years since founding), ownership type (state vs. non-state), total assets, and government subsidies.	2014–2022	Derived via a three-stage DEA/SFA framework, with inputs and outputs from CSMAR and iFinD; , and further details appear in Section 4.1, Sample Selection and Data Sources.
Innovation Quality	(2) Patent Records – CNIPA	IPC-based knowledge breadth computed from International Patent Classification main groups at the patent level and aggregated to firm-year.	2014–2022	CNIPA Patent Search and Analysis Database; fields: IPC codes, application and grant dates, assignee; construction and Stata code in Supplementary Methods (**S3 Appendix**).
Digital Intelligence Index	(3) Annual Reports (Text Mining)	Composite index of firm-level digitalization based on keyword frequencies and PCA aggregation.	2014–2022	Annual report texts from CNINFO / SSE / SZSE (mirrored via CSMAR/iFinD); complete keyword dictionary in **S1 Appendix**; KMO/Bartlett and PCA details in Supplementary Methods (**S2 Appendix**).
Cloud Computing Level	(3) Annual Reports (Text Mining)	Keyword frequency index for cloud computing terms.	2014–2022	Subcomponent of the Digital Intelligence Index; dictionary entries in **S1 Appendix**.
Artificial Intelligence Level	(3) Annual Reports (Text Mining)	Keyword frequency index for artificial intelligence and machine learning terms.	2014–2022	Subcomponent of the Digital Intelligence Index; dictionary entries in **S1 Appendix**.

Variable Name	Source Database / Category	Description	Coverage(Years)	Notes
Big Data Level	(3) Annual Reports (Text Mining)	Keyword frequency index for big data analytics terms.	2014–2022	Subcomponent of the Digital Intelligence Index; dictionary entries in **S1 Appendix**.
Blockchain Level	(3) Annual Reports (Text Mining)	Keyword frequency index for blockchain-related terms.	2014–2022	Subcomponent of the Digital Intelligence Index; dictionary entries in **S1 Appendix**.
Digital Technology Application Level	(3) Annual Reports (Text Mining)	Keyword frequency index for adoption of digital technologies in business applications.	2014–2022	Subcomponent of the Digital Intelligence Index; dictionary entries in **S1 Appendix**.
Proportion of Largest Shareholder(**Top1**)	(1) Financial Data – CSMAR; iFinD	Ownership concentration measured by the largest shareholder's shareholding ratio.	2014–2022	Financial control variable; captures the impact of ownership structure.
Number of Board Members(**Board**)	(1) Financial Data – CSMAR; iFinD	Total number of directors on the board.	2014–2022	Financial control variable; controls for governance effects.
Industry Competition Level(**HHI**)	(1) Financial Data – CSMAR; iFinD	Herfindahl–Hirschman Index (HHI) at the industry level computed from firm sales.	2014–2022	Financial control variable; HHI to capture competitive pressure.
Asset Turnover Ratio(**ATO**)	(1) Financial Data – CSMAR; iFinD	Operating revenue divided by total assets.	2014–2022	Financial control variable; indicates the efficiency of asset utilization
Operating Cycle(**Cycle**)	(1) Financial Data – CSMAR; iFinD	Average days from cash outflows to inflows .	2014–2022	Financial control variable; serving as a proxy for operational efficiency
Operating Cash Flow(**OCF**)	(1) Financial Data – CSMAR; iFinD	Net operating cash flow scaled by total assets.	2014–2022	Financial control variable; controls for the firm's liquidity and financial health.
Return on Assets (**ROA**)	(1) Financial Data – CSMAR; iFinD	Net income divided by total assets.	2014–2022	Financial control variable;accounts for overall profitability and financial efficiency.
Industry Classification	(1) Financial Data – iFinD	Industry code (GICS as reported).	2014–2022	Used to compute knowledge breadth adjusted by industry means; detailed classifications are provided in **S6 Appendix**.
Ownership Type (state vs. non-state)	(1) Financial Data – iFinD	Indicator of state ownership for heterogeneity analysis.	2014–2022	
Region Type (East vs. Central/West)	(1)Financial Data – iFinD	Indicator of regional group (East vs. Central/West) for heterogeneity analysis.	2014–2022	
Industry Type (Manufacturing vs. Non-manufacturing)	(1)Financial Data – iFinD	Indicator of industry group (Manufacturing vs. Non-manufacturing) for heterogeneity analysis.	2014–2022	

Note: (1) Financial Data obtained from CSMAR and iFinD; (2) Patent Records collected from CNIPA; (3) Text-based variables constructed from annual reports via keyword frequency analysis (dictionary in **S1 Appendix**).(4)Financial and patent data were obtained from authoritative and continuously maintained databases (CSMAR, iFinD, and CNIPA), which are widely used in empirical research on Chinese listed enterprises.
