# Supplementary material for: The impact of digital intelligence technologies on innovation performance: Evidence from specialized, refined, differential and innovative enterprises
Source: PLoS One. 2026 Feb 10;21(2):e0339567. doi: 10.1371/journal.pone.0339567 (PMC12890174; doi:10.1371/journal.pone.0339567)
Supplement: S5 Appendix — (PDF) [file pone.0339567.s005.pdf]

S5 Appendix . Three-Stage DEA Computation Steps

This appendix details the three-stage data envelopment analysis workflow used in the study. Software versions are DEAP 2.1 for DEA and FRONTIER 4.1 for the stochastic frontier adjustment. We adopt an input-oriented BCC specification with variable returns to scale.

Stage 1: Baseline DEA and slack retrieval.

Input and output data are imported into DEAP 2.1. We estimate input-oriented BCC efficiency scores for each firm-year and export the associated input slack variables.

Stage 2: Environmental adjustment via SFA.

Input slacks from Stage 1 are regressed on observed environmental variables in FRONT 4.1 using a stochastic frontier specification. The fitted components are used to adjust inputs for operating environment and statistical noise, yielding environment-purged inputs.

Stage 3: Final DEA on adjusted inputs.

Adjusted inputs and the original outputs are re-estimated in DEAP 2.1 under the same input-oriented BCC model to obtain efficiency scores net of environmental and random disturbances.
