# Supplementary material for: The impact of digital intelligence technologies on innovation performance: Evidence from specialized, refined, differential and innovative enterprises
Source: PLoS One. 2026 Feb 10;21(2):e0339567. doi: 10.1371/journal.pone.0339567 (PMC12890174; doi:10.1371/journal.pone.0339567)
Supplement: S6 Appendix — (PDF) [file pone.0339567.s006.pdf]

S6 Appendix. GICS Sector Classification

While the full GICS (Global Industry Classification Standard) system includes 11 sectors, this study focuses on the following 9 sectors as relevant to the sample of SRDI enterprises in China. The excluded sectors, Real Estate and Financials, were not included in the analysis due to the specific nature of the sample, which mainly consists of firms from industries directly related to technological innovation and industrial upgrading. Note that in firm-level efficiency and corporate-finance research, Real Estate and Financials are often analyzed separately or excluded due to their distinct regulatory frameworks, intermediation-driven balance sheets, and revenue recognition regimes, which render financial ratios not directly comparable to non-financial industries. The sectors considered in this study are as follows:

Table 1 GICS Sector Classification

Sector Name	Description
Consumer Discretionary	Non-essential goods and services that consumers purchase.
Industrials	Includes companies involved in manufacturing and infrastructure.
Utilities	Companies providing essential services such as water, electricity, and gas.
Energy	Includes companies involved in the production and distribution of energy.
Consumer Staples	Essential products that consumers purchase regularly.
Communication Services	Companies providing communication services.
Information Technology	Includes companies involved in software, hardware, and IT services.
Healthcare	Companies providing health services, medical devices, and pharmaceuticals.
Materials	Companies involved in the production and extraction of raw materials.
