# Supplementary material for: The impact of digital intelligence technologies on innovation performance: Evidence from specialized, refined, differential and innovative enterprises
Source: PLoS One. 2026 Feb 10;21(2):e0339567. doi: 10.1371/journal.pone.0339567 (PMC12890174; doi:10.1371/journal.pone.0339567)
Supplement: Attachment — Submitted filename: Response to Reviewers.docx [file pone.0339567.s009.docx]

Dear Editor Ahmad,

We sincerely appreciate your review of our manuscript titled “The Impact of Digital Intelligence on Innovation Performance: Evidence from Specialized, Refined, Differential, and Innovative (SRDI) enterprises” (PONE-D-24-53233) and your valuable feedback. We are grateful for the thoughtful comments provided by the reviewer, and we have made comprehensive revisions in response to all the feedback.

Below are our detailed responses to each comment.

**Dear Dr. Muhammad Imran,**

We would like to begin by sincerely thanking you for your valuable and insightful comments on our manuscript. Your feedback has been instrumental in improving the quality of our paper, and we have made several revisions and additions in response to your suggestions. We believe these changes have enhanced the scientific rigor and applicability of our study. Below, we outline the specific improvements made based on your comments.

**Reviewer #1 General comments:**

**The study is limited to SMEs in China, which restricts its applicability to other countries or contexts. While the findings are robust, additional cross-regional comparisons or global contextualization could broaden the scope.**

**While quantitative methods are rigorously applied, the study lacks qualitative insights (e.g., interviews or case studies) to deepen the understanding of digital intelligence's real-world implications for SMEs.**

**Innovation performance is largely measured through patents and financial data, which may not fully capture softer innovation outputs, such as organizational learning or process improvements.**

**While the heterogeneity analysis identifies the stronger effects of digital intelligence on non-state-owned enterprises, this observation is not sufficiently explored in the discussion section.**

**Response:**

First, we would like to thank Dr. Muhammad Imran for his valuable insights. In response to the reviewer’s comments, we have made several revisions and additions to enhance the scientific rigor and applicability of the paper. The specific improvements are as follows:

First, although the study focuses on China’s SRDI enterprises, the concept of SRDI enterprises is derived from Germany’s Hidden Champions and bears many similarities with the concept of niche enterprises in the United States and Japan. Therefore, we believe that although the paper is primarily focused on China, it still has international relevance. Furthermore, China, like other countries (e.g., the United States, Japan, and Germany), places a strong emphasis on digital technologies and the development of innovative SMEs, which makes the conclusions of this study potentially beneficial to SMEs in different national and policy contexts. Second, regarding the measurement of innovation performance, the paper primarily uses patents and financial data as key indicators. We acknowledge that this approach does not fully capture softer innovation outcomes (e.g., organizational learning and process improvements). We have clearly stated this limitation in the manuscript and pointed out that future research could consider incorporating additional measures of softer innovation to more comprehensively assess innovation performance. Finally, in the heterogeneity analysis section, we have expanded our discussion on the impact of digital intelligence on non-state-owned enterprises and provided a more in-depth analysis, incorporating practical examples to further illustrate the practical implications of our findings.

1. **Reviewer #1 Comment on Abstract:**

**The abstract clearly presents the study’s objectives, methods, and key findings. However, it could briefly mention the practical implications to engage a broader audience.**

**Response:**

We sincerely appreciate the reviewer’s insightful suggestion to incorporate practical implications in the abstract. In response, we carefully considered how best to highlight the practical relevance of our study for a wider audience. While we initially explored various ways to integrate practical implications throughout the abstract, we found that due to the technical nature of the research and its focus on the theoretical mechanisms of digital intelligence and financing barriers, a concise statement would be the most effective way to maintain clarity and focus. Therefore, we have added the following statement to the revised abstract:

“These findings provide valuable insights for policymakers and enterprise decision-makers on leveraging digital intelligence to overcome financing barriers and foster innovation in small and SMEs”.

We believe this addition helps bridge the gap between theoretical findings and practical applications while maintaining the abstract’s primary focus. We hope this approach addresses the reviewer’s concerns while preserving the integrity of our research.

Additionally, in light of the evolving understanding of the concept of Specialized, Refined, Differential, and Innovative (SRDI) enterprises among Chinese scholars, we have updated the terminology to align with the current academic discourse. Furthermore, we have refined the abstract’s wording to enhance its clarity and coherence. **The specific changes are highlighted in red font on the page second of the “Revised Manuscript with Track Changes” document.**

We hope this revision effectively addresses the reviewer’s concern and enhances the broader applicability of our study.

2. **Reviewer #1 Comment on Introduction:**

The introduction effectively outlines the problem and significance of the study. To enhance clarity, a more detailed explanation of the novelty of the "Specialized, Fined, Peculiar, and Innovative" framework would be beneficial.

**Response:**

We would like to express our sincere gratitude to Reviewer for the valuable feedback and constructive suggestions. In response to the reviewer’s comment on enhancing the explanation of the novelty of the SRDI enterprises framework in the introduction, we have expanded the discussion on the specific attributes of SRDI enterprises based on relevant literature and official documents.We have provided a more detailed explanation of the four key components of SRDI enterprises as follows:

**Specialized**: Focusing on core business areas to enhance capabilities in specialized production, services, and collaboration. These enterprises provide components, parts, supporting products, and services to large enterprises, major projects, and industrial chains.

**Refined**: Emphasizing refined production, management, and services. These enterprises gain an advantage in niche markets by offering high-quality products and services that are highly regarded for their cost-effectiveness and superior quality.

**Differential**: Leveraging unique resources to promote traditional craftsmanship and regional culture. By adopting distinctive techniques, technologies, formulas, or raw materials, they produce products with local or enterprise-specific characteristics.

**Innovative**: Engaging in technological, management, and business model innovations to cultivate new growth areas and create new competitive advantages.

We would like to clarify that our initial intention was to provide a comprehensive explanation of SRDI enterprises for both you and the readers. However, we also realized that offering a detailed description of SRDI enterprises resulted in an excessive manuscript length. Therefore, to enhance the focus and coherence of our manuscript, we have streamlined the discussion by retaining only the key characteristics of SRDI enterprises that are directly relevant to our research. This revision not only improves readability but also ensures that our theoretical framework remains concise and well-aligned with the study’s objectives. **The specific changes are highlighted in red font on the page three of the “Revised Manuscript with Track Changes” document.**

This enhanced explanation aims to clarify the unique characteristics of SRDI enterprises and provide readers with a deeper understanding of their essential features. We hope these revisions help strengthen the manuscript's clarity and readability.

3. **Reviewer #1 Comment on Literature Review:**

The review provides a comprehensive summary of related studies but could include a deeper critique of existing gaps to highlight the study’s contribution.

**Response:**

We sincerely appreciate the reviewer’s insightful suggestion to enhance the critique of existing literature. In response to this, we have expanded the discussion on the gaps in the current literature, particularly in the context of China’s innovation landscape. Specifically, we highlight the persistent issues at both the micro and macro levels, such as strategic innovation driven by quantity rather than quality, and the prevalent phenomenon of “quantity-driven, quality-deficient” innovation. We also note that existing studies on patents tend to emphasize quantity over quality, which may introduce biases in the interpretation of research findings.

To provide further context, it is important to note that, since the post-economic-crisis era, China has created what is often referred to as the “China Miracle” in innovation, primarily driven by patent growth. According to the 2023 World Intellectual Property Indicators report by the World Intellectual Property Organization (WIPO), China has been the world leader in patent filings for five consecutive years, with 1.64 million invention patents filed in 2023 alone. Among these, corporate patent filings accounted for 1.13 million, ranking China 11th globally in terms of enterprise innovation. However, this rapid patent growth has also exposed significant issues, including low rates of commercialization and industrialization of research outcomes, as well as an increasing share of non-invention patents. These factors indicate that, although the quantity of innovation output has grown explosively, the quality of innovation may not have kept pace.

While we did not include these specific details in the manuscript due to space limitations, they were instrumental in shaping our critique of existing research and informing the revisions made to our paper. We hope that these additions further emphasize the contribution of our study by addressing these critical gaps in the literature. **The specific changes are highlighted in red font on the page ten of the “Revised Manuscript with Track Changes” document.**

We hope these revisions effectively highlight the contribution of our study by addressing these critical gaps in the literature.

4. **Reviewer #1 Comment on Methodology:**

The methodology is robust, but additional justification for selecting certain control variables (e.g., asset turnover ratio) would strengthen this section.

**Response:**

We thank the reviewer for the valuable suggestion to provide additional justification for the selection of certain control variables. In response to this, we have strengthened the explanation for the inclusion of these variables in our analysis. Specifically, we now provide a more detailed rationale for each control variable used in the study:

**Ownership Concentration (Top1)**: measured by the largest shareholder’s share ratio, reflects the influence of ownership structure on innovation performance. Greater concentration can centralize decision-making, shaping strategic direction, innovation priorities, and resource allocation.

**Board Size (Board)**: indicated by the number of sitting directors, is considered as it may reflect the firm’s governance quality. A larger board may provide diverse perspectives, potentially fostering a more innovation-driven environment. However, excessively large boards could introduce coordination challenges that might hinder timely and effective decision-making.

**Market Competition (HHI)**: measured by the Herfindahl-Hirschman Index of the industry, controls for competitive pressures on innovation. Higher market concentration (higher HHI) may reduce competition, weakening innovation incentives, while lower concentration intensifies competition, fostering innovation.

**Asset Turnover Ratio (ATO)**: calculated as the ratio of net operating revenue to average total assets, reflects how effectively a firm utilizes its assets to generate revenue. A higher ATO suggests better asset use, enabling greater resource allocation for innovation. Firms with higher turnover may also be more agile in deploying resources for technological advancements.

**Operating Cycle (Cycle)**: which refers to the time taken from acquiring inventory to selling it and recovering cash, is included to control for the firm’s operational efficiency. Firms with shorter cycles manage working capital better, enabling more financial resources for innovation.

**Operating Cash Flow (OCF)**: represented by the net cash flow generated from operating activities, controls for the financial health of the firm. Strong OCF reflects the firm’s ability to generate internal funds, reducing reliance on external financing and supporting stable innovation investments.

**Return on Assets (ROA)**: measured by the ratio of earnings before interest and taxes to average total assets, is included to account for the firm’s overall financial performance and efficiency. A higher ROA indicates better profitability and asset management, enhancing the firm’s ability to fund and sustain innovation.

After careful revision, we realized that providing detailed explanations of the control variables within the main text might make the paper overly lengthy and distract from the primary discussion. To maintain clarity and conciseness, we have streamlined this section while ensuring that all necessary details remain accessible to readers. Although we have omitted the detailed descriptions from the main text, the rationale and selection criteria for the control variables are still rigorously justified in the methodology section. We believe this approach enhances the manuscript’s readability while maintaining its academic rigor. **The specific changes are highlighted in red font on the page nineteen the “Revised Manuscript with Track Changes” document.**

By providing clear justifications for each of the control variables, we hope to strengthen the robustness of the variable selection and enhance the overall credibility of our methodology.

5. **Reviewer #1 Comment on Results and Discussion:**

The results are well-presented and supported by appropriate statistical analysis. However, the discussion could better contextualize the findings in relation to broader global trends in digital intelligence adoption.

**Response:**

We thank the reviewer for the helpful suggestion to better contextualize our findings within broader global trends in digital intelligence adoption. We acknowledge that the original manuscript primarily presented basic regression results without delving into the implications and connections to real-world trends. To address this, we have expanded the discussion section to include a deeper exploration of the global digital transformation trend, particularly focusing on the adoption of digital intelligence technologies and their role in driving innovation. Specifically, we have added the following content:

The ongoing global digital transformation trend increasingly recognizes the application of digital intelligence technologies as a key driver of innovation. In particular, SMEs in leading economies such as the United States, Germany, and Japan have been rapidly adopting digital technologies to enhance their innovation capabilities. National initiatives such as Germany’s “Industry 4.0” and Japan’s “Society 5.0” underscore the integration of smart technologies across both manufacturing and service sectors. Similarly, in China, the adoption of digital intelligence—encompassing artificial intelligence and data-driven technologies—has played a pivotal role in accelerating innovation within specialized enterprises. A prime example is the recently launched R1 model by DeepSeek, which has surpassed OpenAI in several tests and challenged Nvidia’s “computing power myth”. The R1 model demonstrates that, through algorithmic optimization, 60% of global AI computing power can be supported by mid-range chips, thus eliminating the need for costly high-performance chips. This breakthrough highlights AI’s transformative potential in reducing dependence on expensive infrastructure, while also underscoring China’s growing influence in the digital intelligence arena.

In this context, we emphasize that smart technologies like AI and machine learning have a more significant impact on innovation than other digital intelligence factors such as digitalization or data-driven technologies. While the latter are essential for data collection and processing, their role in innovation is often limited to enhancing operational efficiency. In contrast, smart technologies utilize advanced algorithms to not only analyze data but also to predict trends, optimize operations, and adapt to market changes. This predictive and adaptive capability accelerates and enhances innovation, distinguishing smart technologies from other digital tools.

Additionally, we reference a 2024 report by the Oxford Economics Institute and Cognizant Impact, which predicts that over the next decade, generative artificial intelligence will increase U.S. productivity by 1.7% to 3.5%, contributing to annual GDP growth ranging from $477 billion to $1 trillion. This provides further evidence of the profound impact that digital intelligence technologies, especially AI, are poised to have on global innovation and productivity. **The specific changes are highlighted in red font on the page twenty-five to twenty-six of the “Revised Manuscript with Track Changes” document.**

We hope that these additions help contextualize our research findings within the broader global trends and enhance the robustness of our study.

6. **Reviewer #1 Comment on Conclusion:**

The conclusion summarizes the findings well but should emphasize future research directions, such as longitudinal studies or comparisons across different industries.

**Response:**

We thank the reviewer for the constructive suggestion to emphasize future research directions in the conclusion. We acknowledge that the original manuscript did not sufficiently address this aspect. To remedy this, we have added the following discussion on future research directions:

In future research, we plan to investigate SMEs similar to the Specialized, Refined, Differential, and Innovative enterprises, such as Germany’s “Hidden Champions”, and niche firms in the United States and Japan. These enterprises share developmental characteristics with China’s specialized enterprises. Given the global trend of digital intelligence development, examining how innovation-driven SMEs can effectively leverage digital technologies to drive high-quality innovation will be a key direction for future studies. Additionally, while our study differentiates between manufacturing and non-manufacturing industries, we see an opportunity to further refine this analysis by exploring the impact of digital intelligence technologies on strategic emerging industries within the manufacturing sector. This deeper exploration could yield valuable insights into the sector-specific application of digital transformation technologies. **The specific changes are highlighted in red font on the page thirty-nine of the “Revised Manuscript with Track Changes” document.**

By including these future research directions, we aim to strengthen the manuscript’s structure and provide a more comprehensive outlook on the ongoing and future trends in digital intelligence adoption and innovation.

We sincerely appreciate Dr. Muhammad Imran’s valuable comments, which have been incredibly helpful in improving the quality of our manuscript. Although we have made every effort to address the suggestions and revisions to the best of our ability, we understand that our changes may still not fully meet the reviewer’s expectations. We would be truly grateful for any further comments or guidance you may have, as we are committed to improving the manuscript to the highest standard.

Thank you once again for your time and valuable input.

**Dear Dr. Irma Nur Afiah,**

We would like to sincerely thank you for your thoughtful and detailed feedback on our manuscript titled The Integration of Digital and Intelligent Technologies for the Innovative Growth of Specialized, Fine, Peculiar, and Innovative SMEs in China. Your comments and suggestions are highly appreciated and have provided us with valuable insights to improve the clarity, depth, and contribution of our research.

Below, we have carefully addressed each of the points raised in your review. We hope that the revisions we have made will meet your expectations and improve the quality of the manuscript.

1. **Reviewer #2 Comment on Introduction:**

The introduction provides a good overview of the topic, but it could benefit from more detailed context regarding the importance of SMEs in China’s economy. Elaborating on the specific challenges faced by SMEs in this sector, particularly in terms of innovation and financing, would help set the stage for your study.

Consider explaining more about the term “Specialized, Fine, Peculiar, and Innovative SMEs” and why this specific category was chosen for the research.

**Response:**

we have enhanced the introduction by addressing the following points: First, we have clarified the concept of Specialized, Refined, Differential, and Innovative(SRDI) enterprises by reviewing relevant literature and official documents. We have provided a more detailed explanation of the four key components of SRDI enterprises as follows: **Specialized**: Focusing on core business areas to enhance capabilities in specialized production, services, and collaboration. These enterprises provide components, parts, supporting products, and services to large enterprises, major projects, and industrial chains. **Refined**: Emphasizing refined production, management, and services. These enterprises gain an advantage in niche markets by offering high-quality products and services that are highly regarded for their cost-effectiveness and superior quality. **Differential**: Leveraging unique resources to promote traditional craftsmanship and regional culture. By adopting distinctive techniques, technologies, formulas, or raw materials, they produce products with local or enterprise-specific characteristics. **Innovative**: Engaging in technological, management, and business model innovations to cultivate new growth areas and create new competitive advantages. While we considered expanding on each of these components further, we are mindful of ensuring that the manuscript remains focused and concise. We chose to provide a clear and concise overview of the key characteristics of SRDI enterprises to ensure that readers gain a comprehensive understanding without overwhelming them with excessive detail. We believe that this approach allows us to maintain the clarity and academic rigor of the manuscript while avoiding unnecessary complexity.

Second, we have provided a more comprehensive discussion of the role of SMEs in China’s economy, highlighting their significant contributions to innovation and GDP, while also identifying the key challenges they face, particularly in terms of financing. Finally, we have elaborated on the reasons for selecting SRDI enterprises as the focus of this study. We emphasize that SRDI enterprises play a key role in China’s manufacturing powerhouse strategy and are central to addressing critical technological bottlenecks. By focusing on these enterprises, we aim to better explore the role of digital intelligence technologies in driving innovation within SMEs, filling the existing gap in research on the mechanisms of digital intelligence empowerment. **The specific changes are highlighted in red font on the page three to four of the “Revised Manuscript with Track Changes” document.**

We hope these revisions help clarify the objectives of our study and improve the overall quality of the manuscript.

2. **Reviewer #2 Comment on Methodology:**

The description of the empirical investigation is informative, but additional details regarding the data collection process (e.g., the criteria for selecting SMEs, sample size, and potential biases in the dataset) would strengthen the methodology section. Additionally, it would be helpful to discuss any challenges or limitations in the data, particularly concerning missing data or data quality.

**Response:**

We would like to express our sincere gratitude for your valuable feedback on our manuscript. Indeed, we acknowledge that the original version of our manuscript did not provide sufficient details regarding the data processing methods. Following your suggestions, we have revised the methodology section to include more specific information on the data collection and treatment process. Specifically, we have clarified the criteria for selecting SMEs in our study. As the Ministry of Industry and Information Technology of China has published six batches of SRDI enterprises to date, we made sure that our research sample includes enterprises from all six batches. This ensures that the study comprehensively covers SRDI enterprises across different phases.

Additionally, we further elaborated on the data screening process. First, we excluded enterprises with severe missing data. Secondly, due to the limited number of SRDI enterprises in underdeveloped regions such as Tibet, we excluded enterprises from these areas to avoid potential biases related to regional disparities. For the remaining missing data, we employed interpolation techniques to estimate the missing values. After these adjustments, the final sample consisted of 1,100 enterprises, representing all six batches of SRDI enterprises, with a total of 9,990 enterprise-year observations. **The specific changes are highlighted in red font on the page thirteen to fourteen of the “Revised Manuscript with Track Changes” document.**

We hope that these revisions clarify the methodology and address the concerns regarding data selection and quality. We are grateful for your valuable feedback, which has significantly strengthened the rigor of our study.

3. **Reviewer #2 Comment on Key Findings and Analysis:**

Your findings that digital intelligence, particularly intelligent technologies, improve the innovation performance of SMEs are in line with contemporary research and provide valuable insights. I would encourage you to explore in more depth why intelligent technologies had a stronger impact compared to other aspects of digital intelligence (such as datafication or digitization).

The positive impact of digital intelligence on alleviating financing constraints is significant. Could you provide more specific examples of how these SMEs utilized their digital capabilities to overcome these constraints? Furthermore, why do you think non-state-owned enterprises in particular showed more pronounced benefits from digital intelligence adoption? A deeper exploration of these differences could enrich the analysis.

**Response:**

We sincerely appreciate your thoughtful feedback on our findings and discussion. Your insightful suggestions have been truly helpful in identifying the limitations of our original manuscript and refining our analysis. Thank you for your time and effort in improving the clarity and depth of our work.

Thank you for highlighting the need to further explore why intelligent technologies, particularly AI and machine learning, exhibit a stronger impact on innovation than other digital intelligence components such as datafication and digitization. We have revised our discussion to provide a more comprehensive explanation. While datafication and digitization establish a foundation by enabling efficient data collection, storage, and processing, their direct role in driving innovation is often constrained to operational efficiency improvements. In contrast, intelligent technologies not only analyze data but also incorporate advanced algorithmic capabilities to predict trends, optimize processes, and dynamically adapt to changing market conditions. This predictive and adaptive nature allows AI and machine learning to accelerate the innovation cycle and generate more substantial impacts. To illustrate this point, we have included an example of the DeepSeek R1 model, which optimizes AI computing efficiency through advanced algorithms, reducing reliance on high-performance chips. This case demonstrates how intelligent technologies can significantly reshape technological trajectories by enhancing efficiency while reducing infrastructure constraints. Additionally, we have incorporated findings from a 2024 Oxford Economics Institute and Cognizant Impact report, which estimates that generative AI could increase U.S. productivity by 1.7% to 3.5% over the next decade, contributing to an annual GDP growth of $477 billion to $1 trillion. These revisions help clarify the distinct advantages of intelligent technologies over traditional digital intelligence factors. **The specific changes are highlighted in red font on the page twenty-five to twenty-six of the “Revised Manuscript with Track Changes” document.**

We appreciate your suggestion to provide more concrete examples of how SMEs leverage digital intelligence to overcome financing constraints. In response, we have expanded our discussion with specific cases from both China and the United States. In China, according to a 2023 report by the China Banking and Insurance Regulatory Commission (CBIRC), SMEs face a financing gap exceeding 13 trillion RMB, which traditional banking institutions struggle to address. In response, digital lending platforms such as Ant Group and JD Digits have pioneered AI-driven credit assessment models. These platforms use real-time business data, rather than conventional collateral-based evaluations, to provide faster and more flexible financing solutions. This enables SMEs to access much-needed capital, ensuring continued investment in R&D and innovation. Similarly, in the United States, the Federal Reserve Bank of New York reports an estimated $100 billion credit gap for SMEs, primarily due to traditional banks’ reluctance to provide small business loans. Technology-driven lenders such as Kabbage and OnDeck address this issue by leveraging big data analytics and digital platforms to assess creditworthiness in real time. These platforms enable SMEs to secure financing more efficiently, allowing them to maintain a steady trajectory of innovation and technological development despite conventional financial constraints. However, after careful consideration, we decided to focus on the Chinese example in the main text, for the following reasons:

Relevance to the Study’s Core Context: Since the study primarily focuses on the Chinese market and its unique challenges, we felt that the case of Ant Group and JD Digits provided a more relevant and specific illustration of how digital intelligence can alleviate financing constraints within China’s regulatory and financial environment. Adding the U.S. example, while valuable, could shift the focus away from the primary regional context of the study.

Maintaining Conciseness and Focus: In the interest of maintaining the paper’s conciseness and clarity, we opted to limit the number of examples included in the main text. Our goal was to ensure that the core argument is clear and well-supported without introducing unnecessary complexity or length. We believe the Chinese example sufficiently supports the thesis and provides the required depth of analysis.

Avoiding Redundancy: The key points raised by both the Chinese and U.S. examples were similar, especially regarding the role of digital platforms in facilitating SME financing. Including both examples could create redundancy, which might detract from the paper’s overall focus and impact. Therefore, we chose to retain the example most directly relevant to the study.

These additions provide a more concrete foundation for our argument on how digital intelligence facilitates SME financing, reinforcing the practical significance of our findings. **The specific changes are highlighted in red font on the page twenty-eight of the “Revised Manuscript with Track Changes” document.**

Thank you for raising this important question regarding why non-state-owned enterprises (NSOEs) in China derive greater benefits from digital intelligence adoption compared to state-owned enterprises (SOEs). We have expanded our discussion to further clarify this distinction. NSOEs operate in a highly competitive and dynamic market environment, requiring continuous technological adaptation to maintain their market position. This competitive pressure drives NSOEs to proactively integrate AI, big data, and blockchain technologies to enhance operational efficiency and innovation output. In contrast, SOEs often prioritize economic stability and policy alignment over technological agility. Institutional inertia and bureaucratic decision-making processes can slow down the adoption of emerging digital technologies, limiting their capacity to fully leverage digital intelligence for innovation. We have also cited empirical findings that highlight these differences, reinforcing our argument that NSOEs, driven by market competition rather than policy mandates, exhibit a greater urgency and flexibility in adopting digital intelligence technologies to sustain their innovation performance. **The specific changes are highlighted in red font on the page thirty-two to thirty-three of the “Revised Manuscript with Track Changes” document.**

We sincerely hope that our revisions have effectively addressed your concerns and further strengthened the clarity and rigor of our study. Thank you again for your valuable feedback and constructive suggestions.

4. **Reviewer #2 Comment on Implication and Contribution:**

The practical implications of your study for policymakers and SMEs are valuable. It would be beneficial to connect your findings to existing theories or frameworks on digital transformation and innovation management, especially in the context of emerging economies like China. Are there any new theoretical insights that your study contributes to the literature on digital intelligence and SME innovation?

Discussing how your findings could influence future business strategies for SMEs would add value. Specifically, how can SMEs in China leverage digital intelligence for long-term growth?

**Response:**

We would like to express our sincere gratitude for your constructive comments and insightful feedback. Your suggestions have significantly contributed to enhancing both the theoretical depth and practical relevance of our study.

In response to your suggestion, we have strengthened the theoretical foundation by explicitly linking our findings to Schumpeterian innovation theory. This connection underscores how digital intelligence not only enhances efficiency but also fosters breakthrough innovations in SRDI enterprises. Additionally, our study extends the resource-based view (RBV) by incorporating financing constraints as a moderating factor, which highlights how financial limitations impede firms from fully leveraging digital intelligence for innovation. This extension provides novel insights into digital transformation strategies for SMEs, especially in resource-constrained environments. Furthermore, by integrating an institutional perspective into our heterogeneity analysis, we demonstrate how regional, ownership, and industry-specific factors influence the relationship between digital intelligence and innovation. These contributions enrich the theoretical framework of digital transformation, offering a more nuanced understanding of its impact in the context of emerging economies. **The specific changes are highlighted in red font on the page five of the “Revised Manuscript with Track Changes” document.**

We have also expanded the discussion on how SMEs, particularly in China, can leverage digital intelligence for long-term growth. Specifically, we propose that SMEs accelerate the adoption of cloud computing and AI technologies, which can enhance decision-making, optimize operations, and drive innovation, all while minimizing the need for significant capital investments. Furthermore, we discuss how the use of blockchain and big data analytics can improve financing access for SMEs by enhancing transparency, reducing information asymmetry, and improving creditworthiness. These aspects are particularly pertinent in China’s financial landscape, where access to capital remains a persistent challenge for SMEs. Additionally, we emphasize the potential of digital technologies in facilitating international expansion. By utilizing cross-border e-commerce and digital marketing platforms, SMEs can overcome geographic constraints and strengthen their competitive positioning in global markets. These revisions reinforce the connection between our findings and existing theoretical frameworks, while also offering actionable insights for SMEs navigating digital transformation in emerging markets. **The specific changes are highlighted in red font on the page thirty-eight to thirty-nine of the “Revised Manuscript with Track Changes” document.**

We are grateful for your valuable feedback, which has significantly improved the clarity and depth of our study, and we look forward to any further suggestions you may have.

5. **Reviewer #2 Comment on Limitation and Future Research:**

While your study offers valuable insights, it would be beneficial to address the limitations of your research more explicitly. For example, are there external factors, such as policy changes or economic conditions, that could have impacted the SMEs during your study period?

I suggest that future research explore the long-term effects of digital intelligence on SME innovation, and examine how digital transformation interacts with other factors, such as government policy and industry-specific trends.

**Response:**

We sincerely appreciate your insightful feedback, which has significantly contributed to refining our analysis and enhancing the clarity of our discussion. We acknowledge that our original manuscript did not sufficiently elaborate on the study’s limitations, and we have now incorporated a more explicit discussion in the revised version.

During the study period, SRDI enterprises encountered several external challenges that likely influenced their innovation activities. For instance, China’s supply-side structural reforms led to rising raw material costs in key manufacturing sectors, intensifying financial pressures on SMEs. The COVID-19 pandemic further exacerbated these difficulties by disrupting supply chains, particularly in the semiconductor industry—delaying product development for technology-driven firms. Additionally, while the 2022 VAT rebate policy was introduced to alleviate financial burdens, its uneven implementation restricted access for certain non-state-owned enterprises, potentially limiting their ability to leverage digital intelligence for innovation.

A concrete example can be observed in Shandong Province, where the industrial transformation initiative launched in 2018 aimed to modernize traditional industries by promoting advanced manufacturing and new energy sectors. Although designed to drive industrial upgrading, this shift inadvertently led to a significant outflow of enterprises, with more than half of the province’s industrial cities experiencing business relocations to other regions, resulting in an estimated GDP decline of nearly 2 trillion RMB. Similar green development policies in other regions imposed additional constraints on manufacturing enterprises, further impacting their capacity to adopt digital intelligence technologies. However, after careful consideration, we chose not to include this example in the main text for the following reasons:

**Maintaining Focus on Core Research Theme**: The case of Shandong, although insightful, addresses the unintended consequences of green development policies, which we believe might divert the reader's attention from the central focus of how SMEs use digital intelligence to overcome financing constraints. Including this example could potentially shift the manuscript’s emphasis.

**Broader Context**: The challenges observed in Shandong are part of a broader phenomenon seen in multiple regions, and while relevant, they are somewhat tangential to the primary focus of our paper. We felt that the other examples already provided a clear and representative overview of the broader challenges SMEs face.

**Conciseness and Clarity**: Given the manuscript’s focus and length constraints, we aimed to present the most directly relevant cases to ensure clarity and conciseness. While the Shandong example is important, including it might add unnecessary complexity to the argument. We hope this explanation clarifies our reasoning.

At the same time, we have carefully considered your suggestions regarding future research directions. In this regard, future studies could explore the long-term effects of digital intelligence on SME innovation by examining the interaction between digital transformation and government policies, particularly the SRDI enterprises recognition policy in China. This policy, which aims to support high-tech, specialized SMEs, could serve as a critical factor in understanding how SMEs adopt digital technologies and innovate within the framework of government incentives and industry regulations. Furthermore, future research could investigate how digital transformation interacts with industry-specific trends, such as the shift towards green manufacturing or the adoption of emerging technologies in sectors like semiconductors. Such an approach would provide valuable insights into how SMEs can leverage digital intelligence to foster innovation across diverse industries and policy environments. However, due to space limitations, a more detailed discussion of these future research directions has not been included in the manuscript. **The specific changes are highlighted in red font on the page thirty-nine to forty of the “Revised Manuscript with Track Changes” document.**

We hope these revisions provide a more comprehensive discussion of the study’s limitations. Once again, we sincerely appreciate your constructive feedback.

6. **Reviewer #2 Comment on References:**

The references section could be enhanced by incorporating more recent studies on digital intelligence in SMEs, particularly those focusing on the Chinese context. Additionally, including references to works that explore government policies on digital transformation in China would further contextualize your findings.

**Response:**

Thank you for your insightful comments and suggestions regarding the references section of our manuscript. We appreciate your recommendation to incorporate more recent studies on digital intelligence in SMEs, particularly those focusing on the Chinese context, as well as works that explore government policies on digital transformation in China. Based on your feedback, we have revised the references section to better reflect the context of digital transformation in China.

In response to your suggestions, we have added three recent and relevant studies to our reference list to enhance the depth of our research. Firstly, we have included the work by Ma et al. (2024), which explores the role of resource orchestration in enabling SMEs to leverage digital opportunities. This study provides valuable insights into how traditional resources alone are insufficient for digital transformation, and highlights the necessity of digital platform capabilities for SMEs to effectively utilize digital opportunities. This is particularly relevant to our study, as it guides SMEs facing the challenge of “having resources but not knowing how to use them”. **The specific changes are highlighted in red font on the page six of the “Revised Manuscript with Track Changes” document.**

Secondly, we have referenced the article by Yao et al. (2024), which discusses how artificial intelligence can improve firm productivity by shifting the demand for labor from low-skilled to high-skilled workers. This study provides a crucial link between labor structure adjustment and firm productivity, directly aligning with the theme of technological transformation in our work. **The specific changes are highlighted in red font on the page seven of the “Revised Manuscript with Track Changes” document.**

Finally, we have incorporated the study by Liu et al. (2024), which investigates the impact of digital government initiatives on the quality upgrading of China’s export products. This article helps contextualize our findings within the broader framework of government policies on digital transformation in China, which is particularly relevant given the importance of government-driven digitalization in driving economic growth and trade development. **The specific changes are highlighted in red font on the page six of the “Revised Manuscript with Track Changes” document.**

In fact, there has been a growing body of research in China focused on digital intelligence in SMEs. We have carefully selected a few of the most representative studies to illustrate the current developments in digitalization in China. We believe that these additions significantly strengthen the theoretical grounding and practical implications of our research for the following reasons:

**Relevance to Current Literature**: The selected studies provide insights into the ongoing trends in digital transformation within Chinese SMEs, helping to position our research in the context of current developments. We believe these references demonstrate how our work aligns with the current discourse on digital intelligence in China.

**Enhancing Theoretical Depth**: By incorporating these studies, we aim to ground our research in recent and relevant literature. These references reinforce the theoretical foundation of our study, ensuring that it is supported by up-to-date academic perspectives on digital transformation in SMEs.

**Strengthening Practical Implications**: The selected studies also enhance the practical significance of our findings by providing real-world examples of how digital intelligence is being leveraged by SMEs in China. This helps to contextualize our research and highlight its relevance to SMEs seeking to overcome digital challenges.

Thank you once again for your constructive feedback. We look forward to your further comments and suggestions.

We sincerely appreciate the time and effort that the reviewers, Dr. Muhammad Imran and Dr. Irma Nur Afiah, have dedicated to evaluating our manuscript. Their insightful comments have significantly contributed to improving the quality and clarity of our work. We have carefully addressed all the concerns raised and incorporated the necessary revisions accordingly.

Throughout the revision process, we have made every effort to integrate the reviewers’ valuable suggestions while maintaining the methodological rigor of our study. Our primary goal has been to enhance the clarity and coherence of the manuscript, achieving a balanced improvement in academic value and policy relevance. To achieve this, we have refined the presentation of our methodology and findings, improving readability while preserving the depth and integrity of the study. We have also carefully structured the content to reduce any potential reading burden while maintaining the necessary details to support our conclusions.

We hope that the revised manuscript now meets the journal’s standards and aligns with the reviewers' expectations. Should there be any further suggestions or modifications required, we would be more than happy to address them.

**Additionally, regarding Figures 1 and 2, we have included them in the compressed file under Supporting Information. This ensures accessibility while keeping the main manuscript concise. We appreciate the reviewer’s attention to detail and hope this format aligns with the journal’s submission requirements.**

**Furthermore, we have supplemented the funding disclosure on page 44 with the following statement:**

**"This work was partially supported by the Shandong Social Science Foundation (No. 22CJJJ32)."**

Thank you once again for your time and consideration. We look forward to your feedback.

Best regards,

All authors
