# Supplementary material for: The impact of digital intelligence technologies on innovation performance: Evidence from specialized, refined, differential and innovative enterprises
Source: PLoS One. 2026 Feb 10;21(2):e0339567. doi: 10.1371/journal.pone.0339567 (PMC12890174; doi:10.1371/journal.pone.0339567)
Supplement: Attachment — Submitted filename: Response_to_Reviewers_auresp_2.docx [file pone.0339567.s010.docx]

Dear Editor Ahmad,

We sincerely appreciate your review of our manuscript titled “The Impact of Digital Intelligence on Innovation Performance: Evidence from Specialized, Refined, Differential, and Innovative (SRDI) enterprises” (PONE-D-24-53233R1) and your valuable feedback. We are grateful for the thoughtful comments provided by the reviewer, and we have made comprehensive revisions in response to all the feedback.

Below are our detailed responses to each comment.

**Dear Reviewer #2 and Reviewer #3**

We would like to begin by sincerely thanking you for your valuable and insightful comments on our manuscript. Your feedback has been instrumental in improving the quality of our paper, and we have made several revisions and additions in response to your suggestions. We believe these changes have enhanced the scientific rigor and applicability of our study. Below, we outline the specific improvements made based on your comments.

1. **Reviewer #3**

The manuscript is not written in standard English. The language is not clear and intelligible.

**Response:**

We sincerely thank the reviewer for this valuable comment. In order to address the language concern, we have engaged a professional English-language editing service. The entire manuscript has been carefully polished to ensure that the writing is clear, correct, and unambiguous. We have also uploaded the official editing certificate as a supplementary file (Certificate), which confirms that the manuscript has undergone professional language editing. We hope this resolves the reviewer’s concern.

2. **Reviewer #3 Comment on Abstract:**

Fix minor typos (e.g., duplicate phrase: “intelligent technologies intelligent technology”). Add one line on practical implications to connect with broader readers.

**Response:**

We sincerely appreciate the reviewer’s careful reading of our manuscript and the constructive feedback provided. Thank you for pointing out the minor typographical errors in the Abstract and for suggesting that we add a line on the practical implications of our research to make our findings more accessible to a broader readership. We value these suggestions and have addressed them as follows:

We have carefully reviewed and corrected any typographical errors in the Abstract, including removing duplicate phrases. In addition, we have added a sentence summarizing the practical implications of our findings to broaden the relevance and impact of our research.

The revised Abstract now reads as follows:

“These findings provides valuable guidance for policymakers seeking to develop targeted and differentiated strategies to enhance the innovation capacity of SRDI enterprises. In particular, the results underscore the role of digital intelligence in easing financing constraints. By accounting for firm-level heterogeneity, this study offers a robust theoretical and empirical foundation for policy design and delivers actionable recommendations for enterprises aiming to optimize resource allocation and strengthen innovation capabilities through digital transformation.” **The specific changes are highlighted in red font on the page 2 of the “Revised Manuscript with Track Changes” document.**

We sincerely appreciate the reviewer’s careful and constructive feedback. We hope that the changes we have made are satisfactory. If there are any further questions or suggestions, we would be grateful to receive them and will revise the manuscript accordingly.

3. **Reviewer #3 Comment on Introduction:**

Briefly mention how your findings might be useful for SMEs outside China, especially in other emerging economies.

**Response:**

Thank you for this valuable suggestion. In the revised manuscript, we clarify that our findings are particularly relevant for SMEs in other emerging economies that share similar institutional environments, financial constraints, and industrial structures with China. Our results demonstrate that digital intelligence significantly enhances innovation performance and alleviates financing constraints for SMEs—a mechanism likely to be applicable in countries such as India, Vietnam, Indonesia, and Brazil, where digital transformation in the SME sector is well underway.

Furthermore, our study provides actionable guidance for policymakers and business leaders in economies where the adoption of digital intelligence technologies is still at an early or exploratory stage. For example, in Egypt and Turkey, many SMEs remain cautious about investing in cloud-based services due to concerns over cybersecurity, cost, and a lack of specialized IT staff. In the Philippines and South Africa, small businesses often operate in fragmented markets with limited access to formal finance, making them hesitant to transition from manual to digital operations. Nevertheless, pilot projects—such as Egyptian SMEs collaborating with fintech startups to implement mobile-based accounting systems and Turkish business associations rolling out cloud adoption programs—demonstrate that the practical benefits of digital intelligence are already emerging.

Importantly, the widespread availability of cloud-based platforms enables SMEs in emerging markets to access advanced technological tools without significant up-front investment or specialized technical teams. These solutions lower the barriers to digital adoption and shorten the learning curve, making digital transformation possible even for firms with limited resources.

Although SMEs across countries face different institutional and regulatory environments, the underlying logic of digital intelligence empowerment is consistent: by reducing the costs and complexity of technology acquisition, digital platforms unlock innovation potential and promote sustainable growth in diverse contexts.

Due to space limitations, this discussion has been summarized in a concise statement in the revised Introduction. The revised Abstract now reads as follows:

“Comparable trends are evident in countries like India, Brazil, and Vietnam, where cloud-based platforms and digital financial tools are increasingly used to support SME innovation. In contrast, countries such as Egypt and Turkey have adopted a more cautious approach, with many firms still assessing the opportunities and risks of digital transformation. Although institutional and regulatory conditions vary across countries, the fundamental mechanism by which digital intelligence empowers firms remains consistent. By reducing the costs and barriers associated with technology adoption, digital intelligence enables enterprises to unlock their innovation potential across a range of economic and institutional settings.” **The specific changes are highlighted in red font on page 5 to 6 of the “Revised Manuscript with Track Changes” document.**

We would be happy to provide additional examples or further details if required. Once again, we appreciate the reviewer’s constructive feedback and remain open to further suggestions.

4. **Reviewer #3 Comment on Methodology:**

Include a simple explanation or visual example of how you measured digital intelligence using keyword frequency in reports.

Clarify how the “knowledge breadth” metric was constructed from patent data for readers unfamiliar with IPC codes.

**Response:**

Thank you for your valuable comments. We have addressed each suggestion separately as follows:

Measurement of digital intelligence using keyword frequency:

In response to the reviewer’s suggestion, we have supplemented the Methods section with a concise explanation and a visual example of the keyword frequency method. Specifically, digital intelligence was measured by counting the occurrences of five core digital technology keywords—cloud computing, big data, blockchain, artificial intelligence, and digital technology applications—in the annual reports of all sampled firms. For example, if the report of Firm A contained “cloud computing” 10 times, “big data” 6 times, and “artificial intelligence” 4 times, these values were recorded as the raw digital intelligence scores for that firm, as shown in columns 3 to 7 of Table 1.

The construction of the digital intelligence index involved the following main steps:

(1) standardization of the raw keyword frequencies; (2) correlation testing using the Kaiser-Meyer-Olkin (KMO) and Bartlett’s tests to confirm suitability for factor analysis (with a KMO value greater than 0.723); (3) factor analysis and calculation of eigenvalues and eigenvectors; (4) principal component selection based on eigenvalues and unit eigenvectors, as detailed in the main text; (5) construction of the composite index using the weighted principal components. The principal component loadings (contribution rates), denoted as c1, c2, c3, and c4, were then used to calculate the composite digital intelligence index as follows:

C_total_=（c1×variance contribution of PC1+c2×variance contribution of PC2+c3×variance contribution of PC3+c4×variance contribution of PC4）/cumulative variance contribution of PC4

For clarity, we have also included a summary workflow diagram as follows:

Annual Report Text → Keyword Extraction and Counting → Standardization → Factor Analysis → Index Construction.

We hope that this explanation provides a clear understanding of our procedures. Should you have any further questions or require additional clarification, please let us know. We would be very happy to provide further details.

Table 1 Digital Intelligence Index Data

| code | year | Cloud | Big data | Blockchain | AI | digital technology | c1 | c2 | c3 | c4 | DiginteIndex | lnDiginteIndex |
| --- | --- | --- | --- | --- | --- | --- | --- | --- | --- | --- | --- | --- |
| Firm A | 2014 | 0 | 0 | 0 | 0 | 16 | -0.07 | -0.62 | 0.47 | 0.51 | 0.01 | 0.01 |
| Firm A | 2015 | 0 | 0 | 1 | 0 | 5 | -0.29 | -0.12 | 0.10 | 0.20 | -0.11 | -0.12 |
| Firm A | 2016 | 0 | 0 | 0 | 0 | 16 | -0.07 | -0.62 | 0.47 | 0.51 | 0.01 | 0.01 |
| Firm A | 2017 | 1 | 0 | 0 | 0 | 6 | -0.27 | -0.18 | 0.18 | 0.14 | -0.11 | -0.12 |
| Firm A | 2018 | 0 | 0 | 0 | 1 | 1 | -0.27 | 0.40 | 0.27 | 0.17 | 0.02 | 0.02 |
| Firm A | 2019 | 0 | 0 | 0 | 0 | 2 | -0.41 | 0.01 | 0.04 | 0.09 | -0.17 | -0.19 |
| Firm A | 2020 | 0 | 0 | 0 | 0 | 3 | -0.39 | -0.03 | 0.07 | 0.12 | -0.16 | -0.17 |
| Firm A | 2021 | 0 | 0 | 0 | 0 | 2 | -0.41 | 0.01 | 0.04 | 0.09 | -0.17 | -0.19 |
| Firm A | 2022 | 0 | 1 | 0 | 0 | 2 | -0.35 | 0.01 | -0.03 | 0.11 | -0.15 | -0.17 |
| Firm A | 2023 | 0 | 2 | 0 | 0 | 2 | -0.30 | 0.02 | -0.09 | 0.12 | -0.14 | -0.15 |

Table 2 Results of the multi-grid principal component analysis

| Principal Component | Initial Eigenvalue | | |
| --- | --- | --- | --- |
|  | Eigenvalue | Variance Contribution (%) | Cumulative Variance Contribution (%) |
| 1 | 2.171 | 43.42 | 43.42 |
| 2 | 0.891 | 17.81 | 61.23 |
| 3 | 0.789 | 15.78 | 77.01 |
| 4 | 0.685 | 13.7 | 90.71 |
| 5 | 0.464 | 9.29 | 100 |

Construction of the “knowledge breadth” metric from patent data:

Thank you for your helpful suggestion. In the revised Methods section, we have provided a clear and detailed explanation of how the “knowledge breadth” metric was constructed from patent data, especially for readers unfamiliar with IPC codes.

Specifically, knowledge breadth reflects the diversity of technological fields represented in a firm’s patents. Each patent can be assigned one or more International Patent Classification (IPC) codes, which can be grouped into broader “main groups.” To calculate the knowledge breadth for each patent, we first determined the main groups covered by its IPC codes and the proportion of codes in each main group.

For example, as shown in Table 3, Patent 1 of Firm A has two IPC classification codes: H05B37/02 and H05B37/02. Both belong to the same main group “B37,” so the proportion is 2/2 = 1. According to the formula by Zhang and Zheng, the knowledge breadth for this patent is calculated as:

patent_knowledge = 1 – (2/2)² = 0.

Similarly, Patent 2 of Firm A has three IPC codes—G06F3/045, G06F3/044, and G06F3/045—all belonging to the main group “F3,” giving a proportion of 3/3 = 1 and a knowledge breadth of:

patent_knowledge = 1 – (3/3)² = 0.

Patent 3 of Firm A includes three IPC codes: H02H7/26, H02H1/00, and H02H7/26, covering two main groups (“H7” and “H1”) with proportions of 2/3 and 1/3, respectively. The knowledge breadth is calculated as:

patent_knowledge = 1 – [(2/3)² + (1/3)²] = 0.4444.

This approach follows the method of Zhang and Zheng (full citation in the main text) and can also be calculated directly in Stata using the code provided below:

* Import patent data

* Split IPC codes into separate variables

split ipc_codes, parse(" ") gen(ipc_code_)

* 1. Extract the main group part (before "/") for each IPC classification code

gen main_group_1 = substr(ipc_code_1, 1, strpos(ipc_code_1, "/") - 1)

...

gen main_group_n = substr(ipc_code_n, 1, strpos(ipc_code_n, "/") - 1)

* Generate HHI value for knowledge breadth

* Create a new variable `result` to store the calculation result for each row

gen result = 0

* Iterate over each row of data

forval row = 1/`=_N' {

* Retrieve the quantity for the current row

local quantity = quantity[`row']

* Initialize result to 0

local row_result = 0

* Create a local macro to store unique values for the current row

local unique_values ""

* Iterate through each column (main_group_1 to main_group_n)

foreach col of varlist main_group_1-main_group_n {

* Retrieve the value in the current column

local value = `col'[`row']

* Determine whether the current value already exists in unique_values

if "`value'" != "" & !strpos("`unique_values'", "`value'") {

* If the value is not in unique_values, proceed with counting

* Count the occurrences of the current value in this row

local count = 0

foreach col2 of varlist main_group_1-main_group_n {

if `col2'[`row'] == "`value'" {

local count = `count' + 1

}

}

* Calculate the square of the frequency of this value and add it to row_result

local result_part = (`count' / `quantity')^2

local row_result = `row_result' + `result_part'

* Update unique_values to avoid double-counting

local unique_values "`unique_values' `value'"

}

}

* Store the calculation result for each row in the result variable

replace result = `row_result' in `row'

}

We hope that this clarification improves the accessibility and transparency of our methodology. If you have any further questions or require additional information, please let us know. We would be pleased to provide further details.

Table 3 Example of Knowledge Breadth Calculation

| code | IPC Code | IPC Code1 | IPC Code2 | IPC Code3 | patent_knowledge |
| --- | --- | --- | --- | --- | --- |
| Firm A | H05B37/02 H05B37/02 | H05B37/02 | H05B37/02 |  | 0 |
| Firm A | G06F3/045;G06F3/044 G06F3/045 | G06F3/045 | G06F3/044 | G06F3/045 | 0 |
| Firm A | H02H7/26;H02H1/00 H02H7/26 | H02H7/26 | H02H1/00 | H02H7/26 | 0.4444 |

5. **Reviewer #3 Comment on Results & Analysis:**

Add a few practical examples or short takeaways that SMEs could apply based on your findings.

When describing effects (e.g., digital intelligence improving innovation), consider explaining how big the effect is (if possible).

**Response:**

Add a few practical examples or short takeaways that SMEs could apply based on your findings.

We thank the reviewer sincerely for this valuable suggestion. We agree that providing practical examples and actionable takeaways would greatly enhance the practical relevance of our study. In response, we have added specific summary statements after each main empirical finding in the revised manuscript to guide SMEs on how to apply our research in practice. The main additions are as follows:

Baseline Regression results:

“In summary, the results highlight that in resource-constrained environments, SRDI enterprises should strategically prioritize the adoption of digital intelligence tools, especially cloud computing, artificial intelligence, and digital technology applications, as these exert the strongest positive effects on both innovation efficiency and innovation quality.” **The specific changes are highlighted in red font on page 25 of the “Revised Manuscript with Track Changes” document.**

Mediating Effect Analysis results:

“Such experiences suggest that the adoption of digital finance and analytics platforms could also help SMEs in other countries address similar funding barriers, thereby fostering sustainable innovation.” **The specific changes are highlighted in red font on page 28 of the “Revised Manuscript with Track Changes” document.**

Regional heterogeneity (East-West) results:

“This suggests that, while regional disparities exist in the overall impact of digital intelligence, targeted investment in artificial intelligence technologies could yield substantial gains in innovation quality for SMEs in less developed regions.” **The specific changes are highlighted in red font on page 34 of the “Revised Manuscript with Track Changes” document.**

Sectoral heterogeneity (manufacturing vs. non-manufacturing) results:

“These results highlight the importance of adopting targeted digital strategies. For non-manufacturing SMEs in particular, aligning technological adoption with operational models is critical to maximizing innovation benefits while avoiding unintended negative effects.” **The specific changes are highlighted in red font on page 36 of the “Revised Manuscript with Track Changes” document.**

When describing effects (e.g., digital intelligence improving innovation), consider explaining how big the effect is (if possible).

We thank the reviewer for this helpful suggestion. We agree that specifying the magnitude of the effects would enhance the clarity and informativeness of our findings. In response, we have revised the relevant sections of the manuscript to include quantitative descriptions of the effect sizes. The specific changes are highlighted in red font in the “Revised Manuscript with Track Changes” document. The main additions are as follows:

“When the dependent variable is innovation efficiency, the coefficient for the core explanatory variable—digital intelligence level—is 0.038, statistically significant at the 1% level. This indicates that a 1% increase in the digital intelligence level is associated with an approximate 0.038% improvement in innovation efficiency.” **The specific changes are highlighted in red font on page 24 of the “Revised Manuscript with Track Changes” document.**

“When the dependent variable is innovation quality, the coefficient for the digital intelligence index is 0.125, also statistically significant at the 1% level, indicating a 1% increase in the digital intelligence index corresponds to an approximate 0.125% improvement in innovation quality. At the dimensional level, cloud computing, artificial intelligence, and digital technology applications exhibit positive and significant correlations with innovation quality at the 1% level, whereas big data and blockchain do not. This suggests that, at present, the main drivers of higher innovation quality among SRDI enterprises are the adoption of cloud computing, artificial intelligence, and digital technology applications.” **The specific changes are highlighted in red font on page 24 to 25 of the “Revised Manuscript with Track Changes” document.**

We hope these revisions address the reviewer’s concern and improve the clarity and practical significance of our results. Thank you again for your valuable feedback.

6. **Reviewer #3 Comment on Discussion:**

Try to reduce repetition in sections discussing China’s innovation ecosystem and tech trends. Streamline for clarity.

Emphasize the unique value of intelligent technologies compared to datafication and digitization more concisely.

**Response:**

Try to reduce repetition in sections discussing China’s innovation ecosystem and tech trends. Streamline for clarity：

We thank the reviewer for the helpful suggestion to better contextualize our findings within broader global trends in digital intelligence adoption. We acknowledge that the original manuscript primarily presented basic regression results without delving into the implications and connections to real-world trends. To address this, we have expanded the discussion section to include a deeper exploration of the global digital transformation trend, particularly focusing on the adoption of digital intelligence technologies and their role in driving innovation. Specifically, we have consolidated the discussion as follows:

“In China, the uptake of artificial intelligence and other data-driven technologies has accelerated innovation in specialized enterprises. For example, recent advancements such as DeepSeek’s R1 model demonstrate how algorithmic optimization can reduce reliance on expensive high-performance chips, thereby lowering infrastructure barriers.” **The specific changes are highlighted in red font on the page 25 of the “Revised Manuscript with Track Changes” document.**

Emphasize the unique value of intelligent technologies compared to datafication and digitization more concisely:

We sincerely appreciate the reviewer’s insightful suggestion to more clearly and concisely highlight the unique value of intelligent technologies relative to datafication and digitization. In response, we have carefully revised the relevant section in the manuscript to better articulate this distinction. The revised text now reads:

“Compared with datafication and digitization, intelligent technologies such as AI and machine learning offer distinctive innovation advantages by enabling predictive analytics, adaptive decision-making, and automated optimization, allowing firms to respond more dynamically to market changes.” **The specific changes are highlighted in red font on the page 25 of the “Revised Manuscript with Track Changes” document.**

We hope that these additions help contextualize our research findings within the broader global trends and enhance the robustness of our study.

7. **Reviewer #3 Comment on Conclusion:**

Briefly outline future research directions (e.g., international comparisons, long-term digital effects).
 Add one sentence showing how SMEs can use digital tools for long-term growth, based on your findings.

**Response:**

Briefly outline future research directions (e.g., international comparisons, long-term digital effects).

We sincerely appreciate the reviewer’s insightful suggestion regarding the articulation of future research directions. In response to your comment, we have carefully revised the Conclusion section to more explicitly and thoroughly outline areas for future investigation.

First, we recognize that our study’s focus on SRDI enterprises in China may limit the broader applicability of our findings. To address this limitation, the revised manuscript now emphasizes the value of conducting international comparative studies in future research. We specifically suggest that future studies could analyze similar types of enterprises in different national contexts—such as Germany’s “hidden champions” or niche firms in the United States and Japan—in order to better understand how varying institutional and economic environments influence the relationship between digital intelligence adoption and innovation outcomes. We believe that this direction will greatly enhance the generalizability and policy relevance of subsequent research in this area. The revised future research now reads as follows:

“Future research could.address this by extending the analysis to comparable enterprises in other countries, such as Germany’s “hidden champions” or niche firms in the United States and Japan. Such cross-country comparisons would enable a deeper understanding of how varying institutional frameworks, market structures, and economic environments shape the relationship between digital intelligence and innovation.” **The specific changes are highlighted in red font on the page 39 of the “Revised Manuscript with Track Changes” document.**

Second, we have incorporated a more detailed discussion on the importance of examining the long-term effects of digital intelligence technologies. Given that our analysis is restricted to the period from 2014 to 2022, we acknowledge the need for future research to assess whether the observed positive effects of digital intelligence on innovation performance are sustainable over time. We further suggest that future studies investigate how the adoption of digital technologies shapes organizational dynamics and adaptation processes in the long run, as well as the potential new challenges and opportunities that may emerge as firms deepen their digital transformation. The revised future research now reads as follows:

“Future research could examine the long-term effects of digital intelligence technologies on innovation performance, including whether these impacts are sustainable, how digital adoption reshapes organizational dynamics, and what new challenges or opportunities emerge as enterprises deepen their digital transformation.” **The specific changes are highlighted in red font on the page 39 of the “Revised Manuscript with Track Changes” document.**

Add one sentence showing how SMEs can use digital tools for long-term growth, based on your findings.

Thank you very much for this valuable suggestion. We acknowledge that, in the original manuscript, our discussion of how SMEs could leverage digital intelligence technologies for long-term growth was relatively brief and lacked specific operational guidance. In response to your comment, we have carefully revised the Conclusion section to include a more explicit and actionable statement reflecting our empirical findings.The specific addition is as follows:

“Overall, SMEs can foster sustainable growth by prioritizing the adoption of readily deployable digital intelligence technologies, particularly cloud computing and artificial intelligence, while implementing big data and blockchain solutions selectively, based on specific developmental needs.” **The specific changes are highlighted in red font on the page 37 of the “Revised Manuscript with Track Changes” document.**

We hope this modification addresses the reviewer’s concern and enhances the clarity of our manuscript. Thank you again for your valuable feedback.

We have made these revisions to the best of our ability in accordance with your valuable suggestions. However, we recognize that our modifications may still fall short of your expectations. If you have any further comments or additional suggestions for improvement, please do not hesitate to let us know. We would be very grateful for your continued guidance and are more than willing to make any further revisions necessary to strengthen the manuscript.

Thank you once again for your time and consideration. We look forward to your feedback.

Best regards,

All authors
