# Supplementary material for: The impact of digital intelligence technologies on innovation performance: Evidence from specialized, refined, differential and innovative enterprises
Source: PLoS One. 2026 Feb 10;21(2):e0339567. doi: 10.1371/journal.pone.0339567 (PMC12890174; doi:10.1371/journal.pone.0339567)
Supplement: Attachment — Submitted filename: Point-by-Point Response to Previous Concerns.docx [file pone.0339567.s011.docx]

**Compiled Point-by-Point Response to Reviewers and Editor**

Manuscript ID: PONE-D-24-53233R2

Dear Academic Editor,

As requested, below is a compiled point-by-point response to all concerns raised throughout the submission history, including the three previous revision rounds and the appeal process. Responses are presented in reverse chronological order (most recent first) for easy reference. All changes in the current manuscript are highlighted in the tracked-changes version.

**Response submitted with Revision(Manuscript ID: PONE-D-24-53233R2)**

**Dear Dr. Annesha Sil and Reviewer #3,**

We sincerely appreciate your time and effort in handling our manuscript entitled “The Impact of Digital Intelligence Technologies on Innovation Performance: Evidence from Specialized, Refined, Differential, and Innovative Enterprises” (Manuscript ID: **PONE-D-24-53233R2**). We are grateful for your thoughtful evaluation and for the detailed feedback provided in your letter. We have carefully reviewed each point raised and have revised the manuscript accordingly to ensure full methodological transparency and to clarify the study’s theoretical contribution.

Below, we provide detailed responses to every comment and explain the corresponding revisions made in the revised version and supplementary materials.

1. **Dr. Annesha Sil Comment on data sources:**

Despite two rounds of revision, the exact sources of data for each variable have not been clarified.

The keywords used for the construction of the digitization index have not been provided.

**Response:**

We sincerely appreciate this valuable comment. In the original manuscript, the data sources for all variables were presented in Section 4 (Method and Data, pp. 14–15). However, we recognize that our explanation may not have been sufficiently detailed for readers without a background in economics or management science. To improve clarity and ensure full transparency for an interdisciplinary readership, we have now expanded and refined our descriptions of each data source and variable mapping in both the main text and supplementary materials. Specifically:

(1) Financial variables are now explicitly stated as being obtained from CSMAR and the iFinD Financial Data Terminal, while the SRDI firm lists (batches 1–6) are collected from official MIIT announcements. Patent data used to construct innovation-quality indicators are sourced from the CNIPA Patent Search and Analysis Database, with IPC information consistent with the WIPO IPC scheme. The three-stage DEA model uses input, output and environmental variables derived from these sources. To ensure full transparency, we have added a complete variable–source mapping table in Appendix A4 in S1 Appendix.
(2) The complete list of SRDI enterprises used in this study is now provided in S2 Appendix, corresponding to MIIT batches 1–6.
(3) The computation steps for IPC-based knowledge breadth—including formulas, worked examples, and Stata code—are reported in Appendix A3 in S1 Appendix.
(4) We have added the complete construction workflow and diagnostic procedures for the Digital Intelligence Index in Appendix A2 in S1 Appendix.
(5) the complete keyword dictionary used for identifying cloud computing, big data, artificial intelligence, blockchain, and digital applications is now included in Appendix A1 in S1 Appendix.
(6) The full three-stage DEA computation—implemented using DEAP 2.1 (DEA stages) and FRONTIER 4.1 (stochastic-frontier adjustment)—is documented in Appendix A5 in S1 Appendix.

All of the above data descriptions and supporting materials are fully documented in S1 Appendix. These additions substantially improve the transparency, reproducibility, and completeness of the manuscript. We are grateful for the reviewer’s suggestion, which has helped strengthen the clarity and methodological rigor of the study.

**Table 1. Keyword Dictionary**

| **Dictionary of Digital Intelligence Keywords** | | | | |
| --- | --- | --- | --- | --- |
| **Artificial Intelligence Technology** | **Big Data Technology** | **Cloud Computing Technology** | **Blockchain Technology** | **Digital Technology Application** |
| Artificial Intelligence Technology | Big Data Technology | Cloud Computing Technology | Blockchain Technology | Digital Technology Application |
| Artificial Intelligence | Big Data | Cloud Computing | Blockchain | Mobile Internet |
| Business Intelligence | Data Mining | Stream Computing | Digital Currency | Industrial Internet |
| Image Understanding | Text Mining | Graph Computing | Distributed Computing | Mobile Internet Connectivity |
| Investment Decision Support System | Data Visualization | In-Memory Computing | Differential Privacy Technology | Internet Healthcare |
| Intelligent Data Analysis | Heterogeneous Data | Multi-Party Secure Computation | Smart Financial Contracts | E-Commerce |
| Intelligent Robots | Credit Investigation | Brain-like Computing |  | Mobile Payment |
| Machine Learning | Augmented Reality | Green Computing |  | Third-Party Payment |
| Deep Learning | Mixed Reality | Cognitive Computing |  | NFC Payment |
| Semantic Search | Virtual Reality | Converged Architecture |  | Intelligent Energy |
| Biometric Identification Technology |  | Billion-level Concurrency |  | B2B (Business-to-Business) |
| Facial Recognition |  | EB-level Storage |  | B2C (Business-to-Consumer) |
| Voice Recognition |  | Internet of Things (IoT) |  | C2B (Consumer-to-Business) |
| Identity Verification |  | Cyber-Physical Systems |  | C2C (Consumer-to-Consumer) |
| Autonomous Driving |  |  |  | O2O (Online-to-Offline) |
| Natural Language Processing |  |  |  | Networking |
|  |  |  |  | Smart Wearables |
|  |  |  |  | Smart Agriculture |
|  |  |  |  | Intelligent Transportation |
|  |  |  |  | Intelligent Healthcare |
|  |  |  |  | Intelligent Customer Service |
|  |  |  |  | Smart Home |
|  |  |  |  | Intelligent Investment Advisory |
|  |  |  |  | Intelligent Cultural Tourism |
|  |  |  |  | Intelligent Environmental Protection |
|  |  |  |  | Smart Grid |
|  |  |  |  | Intelligent Marketing |
|  |  |  |  | Digital Marketing |
|  |  |  |  | Unmanned Retail |
|  |  |  |  | Internet Finance |
|  |  |  |  | Digital Finance |
|  |  |  |  | Fintech |
|  |  |  |  | Financial Technology |
|  |  |  |  | Quantitative Finance |
|  |  |  |  | Open Banking |

2. **Dr. Annesha Sil Comment on contribution:**

Major concerns remain about the contribution of this study, as there appear to be several other published works investigating very similar research questions using similar methodologies that have not been adequately cited or discussed.

**Response:**

We sincerely thank the editor for this valuable and constructive comment. We fully understand the importance of situating our contribution within the broader research context. In the earlier version, while we had cited general studies on digitalization and innovation, we acknowledge that we did not explicitly contrast our framework with the most recent and thematically similar empirical works. This omission may have made our theoretical and methodological contribution appear less distinctive than it actually is.

To address this issue comprehensively, we have revised the “Main Contributions” section of the manuscript to clarify how this study extends existing research:

(1)We explicitly highlight that we disaggregate digital intelligence into five distinct technologies (cloud computing, artificial intelligence, blockchain, big data, and digital applications), whereas prior studies commonly rely on a single aggregated digitalisation measure. More importantly, this disaggregation reveals substantial heterogeneity in their effects: while all five technologies enhance innovation efficiency, only cloud computing, artificial intelligence, and digital applications significantly improve innovation quality, and blockchain can even exert a suppressive effect on innovation quality in certain contexts (e.g., state-owned and non-manufacturing firms). This finding underscores that not all digital intelligence technologies are uniformly beneficial, providing a more nuanced understanding than aggregate-level analyses. These results may also offer useful insights for resource-constrained SMEs in other emerging economies, suggesting that firms should adopt digital intelligence technologies selectively according to their own developmental stage and goals. For instance, all five technologies can support innovation efficiency, but prioritizing cloud computing, artificial intelligence, and digital applications appears particularly effective for enhancing innovation quality, whereas blockchain requires cautious application given its limited or context-dependent benefits.

(2) We emphasise our focus on SRDI enterprises, a policy-designated group that has rarely been examined in the digital-transformation literature despite their central role in China’s innovation strategy.

(3) We incorporate financing constraints and institutional characteristics into the analysis, thereby providing new evidence on the mechanisms linking digital intelligence to innovation performance.

(4) We have added targeted discussions and citations to published studies that use comparable empirical approaches or analyse digitalisation and innovation in emerging economies, including research on cloud-based management systems and digital finance in India, digital strategies in Brazilian family-owned firms, and the interaction between digitalisation and knowledge management in Vietnam. These additions clarify how our work builds upon, yet remains distinct from, existing contributions.

These revisions strengthen the clarity, positioning and novelty of the study, and we sincerely appreciate the reviewer’s guidance in improving this aspect. **The specific changes are highlighted in red on pages 5–6 of the “Revised Manuscript with Tracked Changes” document.**

3. **Dr. Reviewer #3Comment :**

I would like to commend the authors for their careful and thorough revisions. The methodology is now clearly explained with illustrative examples, the inclusion of effect sizes strengthens the results, and the discussion has been streamlined to highlight the distinct value of intelligent technologies. The professional language editing has greatly improved readability, **though a few sentences in the introduction and discussion could still be shortened for clarity**. Overall, the paper is now clear, rigorous, and well-prepared for publication.

**Response:**

We sincerely thank Reviewer #3 for the positive and encouraging evaluation. We greatly appreciate your recognition of the improvements made to the manuscript and your helpful suggestion to further enhance clarity.

In response, we have shortened and refined several long sentences in the Introduction section (pp. 2–3) to improve readability and conciseness while maintaining the intended meaning. Specifically, we revised the following sentences:

Original:

“Over the past four decades, China’s economy has experienced rapid transformation, driven largely by reform and opening-up policies that introduced market-oriented mechanisms and expanded integration with the global economy.”

Revised:

“Over the past four decades, China’s economy has transformed through market reforms and global integration.”

Original:

“Differentiation leverages unique resources, traditional craftsmanship, and regional cultural elements to create distinctive products with competitive advantages.”

Revised:

“Differentiation builds on unique resources and regional culture to foster competitive advantages.”

Original:

“They form the backbone of the country’s mid-to high-end manufacturing supply chain, play a central role in high-quality development, and serve as an essential pillar in the new development paradigm, as well as a significant contributor to building an innovative nation.”

Revised:

“They underpin China’s advanced manufacturing and support innovation-driven growth.”

These modifications reduce redundancy and improve fluency while preserving the theoretical accuracy and contextual meaning. We believe this targeted refinement further enhances the clarity, conciseness, and professional readability of the manuscript.**The specific changes are highlighted in red font on the page 2 to 3 of the “Revised Manuscript with Tracked Changes” document.**

**4.Reviewer #3 ((PONE-D-24-53233R1): Comment on Introduction:**

Briefly mention how your findings might be useful for SMEs outside China, especially in other emerging economies.

**Response:**

Thank you for this helpful suggestion. Although we had previously added a brief discussion in response to this comment, the contribution section has been substantially rewritten in the current revision to make the positioning and novelty of the study clearer. Accordingly, we have updated the relevant text to the following:

“These findings on the heterogeneous effects of the five digital intelligence technologies may also inform technology adoption decisions for SMEs in other emerging economies. Specifically, while all five technologies can enhance innovation efficiency and cloud computing, artificial intelligence, and digital applications are particularly effective for improving innovation quality among resource-constrained firms, blockchain may exert suppressive effects on innovation quality in certain contexts. This underscores that not all digital intelligence technologies are uniformly beneficial and highlights the need for cautious, context-specific adoption.”

We hope that this revised formulation more concisely and accurately responds to Reviewer #3’s original comment while fully satisfying *PLOS ONE*’s publishing criteria. The changes are highlighted in the tracked-changes version of the manuscript.**The specific changes are highlighted in red font on the page 6 of the “Revised Manuscript with Tracked Changes” document.**

We sincerely thank the Academic Editor, the Staff Editor, and the reviewers for their time, effort, and constructive feedback throughout the review process. The comments have been invaluable in helping us improve the clarity, methodological transparency, and theoretical contribution of this work.

In the revised version, we have comprehensively addressed all the concerns by clarifying variable-level data sources(S1 Appendix), providing a full keyword dictionary for the digital intelligence index (Fig. 1), refining the introduction and discussion for conciseness, and expanding the contribution section with recent literature to strengthen theoretical grounding.

We greatly value PLOS ONE’s mission to promote cross-disciplinary dialogue and open scientific exchange, and we sincerely hope that this clarification will allow a fair reassessment of the manuscript within its disciplinary context.

Thank you once again for reconsidering our work. We look forward to your decision.

Sincerely,

The Authors

**Response submitted with Revision(Manuscript ID: PONE-D-24-53233R1)**

Dear Editor Ahmad,

We sincerely appreciate your review of our manuscript titled “The Impact of Digital Intelligence on Innovation Performance: Evidence from Specialized, Refined, Differential, and Innovative (SRDI) enterprises” **(PONE-D-24-53233R1**) and your valuable feedback. We are grateful for the thoughtful comments provided by the reviewer, and we have made comprehensive revisions in response to all the feedback.

Below are our detailed responses to each comment.

**Dear Reviewer #2 and Reviewer #3**

We would like to begin by sincerely thanking you for your valuable and insightful comments on our manuscript. Your feedback has been instrumental in improving the quality of our paper, and we have made several revisions and additions in response to your suggestions. We believe these changes have enhanced the scientific rigor and applicability of our study. Below, we outline the specific improvements made based on your comments.

4. **Reviewer #3 Comment on Abstract:**

**Fix minor typos (e.g., duplicate phrase: “intelligent technologies intelligent technology”). Add one line on practical implications to connect with broader readers.**

**Response:**

We sincerely appreciate the reviewer’s careful reading of our manuscript and the constructive feedback provided. Thank you for pointing out the minor typographical errors in the Abstract and for suggesting that we add a line on the practical implications of our research to make our findings more accessible to a broader readership. We value these suggestions and have addressed them as follows:

We have carefully reviewed and corrected any typographical errors in the Abstract, including removing duplicate phrases. In addition, we have added a sentence summarizing the practical implications of our findings to broaden the relevance and impact of our research.

The revised Abstract now reads as follows:

“This study provides important insights for policymakers designing targeted and differentiated policies to improve the innovation performance of SRDI enterprises in China. In particular, the results highlight the role of digital intelligence technologies in alleviating financing constraints. By considering firm heterogeneity, this research offers a solid theoretical and empirical foundation for policy development. The findings also offer practical recommendations for enterprises seeking to use digital intelligence to address resource allocation constraints and strengthen innovation capacity.” **The specific changes are highlighted in red font on the page 2 of the “Revised Manuscript with Track Changes” document.**

We sincerely appreciate the reviewer’s careful and constructive feedback. We hope that the changes we have made are satisfactory. If there are any further questions or suggestions, we would be grateful to receive them and will revise the manuscript accordingly.

5. **Reviewer #3 Comment on Introduction:**

Briefly mention how your findings might be useful for SMEs outside China, especially in other emerging economies.

**Response:**

Thank you for this valuable suggestion. In the revised manuscript, we clarify that our findings are particularly relevant for SMEs in other emerging economies that share similar institutional environments, financial constraints, and industrial structures with China. Our results demonstrate that digital intelligence significantly enhances innovation performance and alleviates financing constraints for SMEs—a mechanism likely to be applicable in countries such as India, Vietnam, Indonesia, and Brazil, where digital transformation in the SME sector is well underway.

Furthermore, our study provides actionable guidance for policymakers and business leaders in economies where the adoption of digital intelligence technologies is still at an early or exploratory stage. For example, in Egypt and Turkey, many SMEs remain cautious about investing in cloud-based services due to concerns over cybersecurity, cost, and a lack of specialized IT staff. In the Philippines and South Africa, small businesses often operate in fragmented markets with limited access to formal finance, making them hesitant to transition from manual to digital operations. Nevertheless, pilot projects—such as Egyptian SMEs collaborating with fintech startups to implement mobile-based accounting systems and Turkish business associations rolling out cloud adoption programs—demonstrate that the practical benefits of digital intelligence are already emerging.

Importantly, the widespread availability of cloud-based platforms enables SMEs in emerging markets to access advanced technological tools without significant up-front investment or specialized technical teams. These solutions lower the barriers to digital adoption and shorten the learning curve, making digital transformation possible even for firms with limited resources.

Although SMEs across countries face different institutional and regulatory environments, the underlying logic of digital intelligence empowerment is consistent: by reducing the costs and complexity of technology acquisition, digital platforms unlock innovation potential and promote sustainable growth in diverse contexts.

Due to space limitations, this discussion has been summarized in a concise statement in the revised Introduction. The revised Abstract now reads as follows:

“While countries like India, Brazil, and Vietnam have already begun to adopt cloud-based platforms and digital financial tools to drive SME innovation, others such as Egypt and Turkey remain more cautious, with many firms still evaluating the benefits and challenges of digital transformation. In summary, although SMEs across countries face different institutional and regulatory environments, the fundamental mechanism of digital intelligence empowerment remains consistent. By lowering the costs and barriers of technology adoption, digital intelligence enables firms to unlock their innovation potential across diverse settings.” **The specific changes are highlighted in red font on page 5 to 6 of the “Revised Manuscript with Track Changes” document.**

We would be happy to provide additional examples or further details if required. Once again, we appreciate the reviewer’s constructive feedback and remain open to further suggestions.

6. **Reviewer #3 Comment on Methodology:**

Include a simple explanation or visual example of how you measured digital intelligence using keyword frequency in reports.

Clarify how the “knowledge breadth” metric was constructed from patent data for readers unfamiliar with IPC codes.

**Response:**

Thank you for your valuable comments. We have addressed each suggestion separately as follows:

Measurement of digital intelligence using keyword frequency:

In response to the reviewer’s suggestion, we have supplemented the Methods section with a concise explanation and a visual example of the keyword frequency method. Specifically, digital intelligence was measured by counting the occurrences of five core digital technology keywords—cloud computing, big data, blockchain, artificial intelligence, and digital technology applications—in the annual reports of all sampled firms. For example, if the report of Firm A contained “cloud computing” 10 times, “big data” 6 times, and “artificial intelligence” 4 times, these values were recorded as the raw digital intelligence scores for that firm, as shown in columns 3 to 7 of Table 1.

The construction of the digital intelligence index involved the following main steps:

(1) standardization of the raw keyword frequencies; (2) correlation testing using the Kaiser-Meyer-Olkin (KMO) and Bartlett’s tests to confirm suitability for factor analysis (with a KMO value greater than 0.723); (3) factor analysis and calculation of eigenvalues and eigenvectors; (4) principal component selection based on eigenvalues and unit eigenvectors, as detailed in the main text; (5) construction of the composite index using the weighted principal components. The principal component loadings (contribution rates), denoted as c1, c2, c3, and c4, were then used to calculate the composite digital intelligence index as follows:

C_total_=（c1×variance contribution of PC1+c2×variance contribution of PC2+c3×variance contribution of PC3+c4×variance contribution of PC4）/cumulative variance contribution of PC4

For clarity, we have also included a summary workflow diagram as follows:

Annual Report Text → Keyword Extraction and Counting → Standardization → Factor Analysis → Index Construction.

We hope that this explanation provides a clear understanding of our procedures. Should you have any further questions or require additional clarification, please let us know. We would be very happy to provide further details.

Table 1 Digital Intelligence Index Data

| code | year | Cloud | Big data | Blockchain | AI | digital technology | c1 | c2 | c3 | c4 | DiginteIndex | lnDiginteIndex |
| --- | --- | --- | --- | --- | --- | --- | --- | --- | --- | --- | --- | --- |
| Firm A | 2014 | 0 | 0 | 0 | 0 | 16 | -0.07 | -0.62 | 0.47 | 0.51 | 0.01 | 0.01 |
| Firm A | 2015 | 0 | 0 | 1 | 0 | 5 | -0.29 | -0.12 | 0.10 | 0.20 | -0.11 | -0.12 |
| Firm A | 2016 | 0 | 0 | 0 | 0 | 16 | -0.07 | -0.62 | 0.47 | 0.51 | 0.01 | 0.01 |
| Firm A | 2017 | 1 | 0 | 0 | 0 | 6 | -0.27 | -0.18 | 0.18 | 0.14 | -0.11 | -0.12 |
| Firm A | 2018 | 0 | 0 | 0 | 1 | 1 | -0.27 | 0.40 | 0.27 | 0.17 | 0.02 | 0.02 |
| Firm A | 2019 | 0 | 0 | 0 | 0 | 2 | -0.41 | 0.01 | 0.04 | 0.09 | -0.17 | -0.19 |
| Firm A | 2020 | 0 | 0 | 0 | 0 | 3 | -0.39 | -0.03 | 0.07 | 0.12 | -0.16 | -0.17 |
| Firm A | 2021 | 0 | 0 | 0 | 0 | 2 | -0.41 | 0.01 | 0.04 | 0.09 | -0.17 | -0.19 |
| Firm A | 2022 | 0 | 1 | 0 | 0 | 2 | -0.35 | 0.01 | -0.03 | 0.11 | -0.15 | -0.17 |
| Firm A | 2023 | 0 | 2 | 0 | 0 | 2 | -0.30 | 0.02 | -0.09 | 0.12 | -0.14 | -0.15 |

Table 2 Results of the multi-grid principal component analysis

| Principal Component | Initial Eigenvalue | | |
| --- | --- | --- | --- |
|  | Eigenvalue | Variance Contribution (%) | Cumulative Variance Contribution (%) |
| 1 | 2.171 | 43.42 | 43.42 |
| 2 | 0.891 | 17.81 | 61.23 |
| 3 | 0.789 | 15.78 | 77.01 |
| 4 | 0.685 | 13.7 | 90.71 |
| 5 | 0.464 | 9.29 | 100 |

Construction of the “knowledge breadth” metric from patent data:

Thank you for your helpful suggestion. In the revised Methods section, we have provided a clear and detailed explanation of how the “knowledge breadth” metric was constructed from patent data, especially for readers unfamiliar with IPC codes.

Specifically, knowledge breadth reflects the diversity of technological fields represented in a firm’s patents. Each patent can be assigned one or more International Patent Classification (IPC) codes, which can be grouped into broader “main groups.” To calculate the knowledge breadth for each patent, we first determined the main groups covered by its IPC codes and the proportion of codes in each main group.

For example, as shown in Table 3, Patent 1 of Firm A has two IPC classification codes: H05B37/02 and H05B37/02. Both belong to the same main group “B37,” so the proportion is 2/2 = 1. According to the formula by Zhang and Zheng, the knowledge breadth for this patent is calculated as:

patent_knowledge = 1 – (2/2)² = 0.

Similarly, Patent 2 of Firm A has three IPC codes—G06F3/045, G06F3/044, and G06F3/045—all belonging to the main group “F3,” giving a proportion of 3/3 = 1 and a knowledge breadth of:

patent_knowledge = 1 – (3/3)² = 0.

Patent 3 of Firm A includes three IPC codes: H02H7/26, H02H1/00, and H02H7/26, covering two main groups (“H7” and “H1”) with proportions of 2/3 and 1/3, respectively. The knowledge breadth is calculated as:

patent_knowledge = 1 – [(2/3)² + (1/3)²] = 0.4444.

This approach follows the method of Zhang and Zheng (full citation in the main text) and can also be calculated directly in Stata using the code provided below:

* Import patent data

* Split IPC codes into separate variables

split ipc_codes, parse(" ") gen(ipc_code_)

* 1. Extract the main group part (before "/") for each IPC classification code

gen main_group_1 = substr(ipc_code_1, 1, strpos(ipc_code_1, "/") - 1)

...

gen main_group_n = substr(ipc_code_n, 1, strpos(ipc_code_n, "/") - 1)

* Generate HHI value for knowledge breadth

* Create a new variable `result` to store the calculation result for each row

gen result = 0

* Iterate over each row of data

forval row = 1/`=_N' {

* Retrieve the quantity for the current row

local quantity = quantity[`row']

* Initialize result to 0

local row_result = 0

* Create a local macro to store unique values for the current row

local unique_values ""

* Iterate through each column (main_group_1 to main_group_n)

foreach col of varlist main_group_1-main_group_n {

* Retrieve the value in the current column

local value = `col'[`row']

* Determine whether the current value already exists in unique_values

if "`value'" != "" & !strpos("`unique_values'", "`value'") {

* If the value is not in unique_values, proceed with counting

* Count the occurrences of the current value in this row

local count = 0

foreach col2 of varlist main_group_1-main_group_n {

if `col2'[`row'] == "`value'" {

local count = `count' + 1

}

}

* Calculate the square of the frequency of this value and add it to row_result

local result_part = (`count' / `quantity')^2

local row_result = `row_result' + `result_part'

* Update unique_values to avoid double-counting

local unique_values "`unique_values' `value'"

}

}

* Store the calculation result for each row in the result variable

replace result = `row_result' in `row'

}

We hope that this clarification improves the accessibility and transparency of our methodology. If you have any further questions or require additional information, please let us know. We would be pleased to provide further details.

Table 3 Example of Knowledge Breadth Calculation

| code | IPC Code | IPC Code1 | IPC Code2 | IPC Code3 | patent_knowledge |
| --- | --- | --- | --- | --- | --- |
| Firm A | H05B37/02 H05B37/02 | H05B37/02 | H05B37/02 |  | 0 |
| Firm A | G06F3/045;G06F3/044 G06F3/045 | G06F3/045 | G06F3/044 | G06F3/045 | 0 |
| Firm A | H02H7/26;H02H1/00 H02H7/26 | H02H7/26 | H02H1/00 | H02H7/26 | 0.4444 |

7. **Reviewer #3 Comment on Results & Analysis:**

Add a few practical examples or short takeaways that SMEs could apply based on your findings.

When describing effects (e.g., digital intelligence improving innovation), consider explaining how big the effect is (if possible).

**Response:**

Add a few practical examples or short takeaways that SMEs could apply based on your findings.

We thank the reviewer sincerely for this valuable suggestion. We agree that providing practical examples and actionable takeaways would greatly enhance the practical relevance of our study. In response, we have added specific summary statements after each main empirical finding in the revised manuscript to guide SMEs on how to apply our research in practice. The main additions are as follows:

Baseline Regression results:

“In summary, these findings suggest that, particularly in resource-constrained environments, it is essential for SRDI enterprises to strategically select and prioritize digital intelligence tools, such as cloud computing, artificial intelligence, and digital technology applications, as these have the greatest impact on both innovation efficiency and quality.” **The specific changes are highlighted in red font on page 26 of the “Revised Manuscript with Track Changes” document.**

Mediating Effect Analysis results:

“This experience suggests that the adoption of digital finance and analytics platforms may help SMEs in other countries address similar financing barriers and potentially foster sustainable innovation.” **The specific changes are highlighted in red font on page 29 of the “Revised Manuscript with Track Changes” document.**

Regional heterogeneity (East-West) results:

“These results suggest that, although the impact of digital intelligence on innovation outcomes varies by region, further investment in artificial intelligence technologies could offer significant potential for improving innovation quality among SMEs in less developed regions.” **The specific changes are highlighted in red font on page 35 of the “Revised Manuscript with Track Changes” document.**

Sectoral heterogeneity (manufacturing vs. non-manufacturing) results:

“These results indicate that adopting a targeted digital strategy is essential for non-manufacturing SMEs that seek to maximize innovation benefits and minimize potential negative impacts. Careful assessment of technology alignment with business operations will be crucial in this process.” **The specific changes are highlighted in red font on page 37 of the “Revised Manuscript with Track Changes” document.**

When describing effects (e.g., digital intelligence improving innovation), consider explaining how big the effect is (if possible).

We thank the reviewer for this helpful suggestion. We agree that specifying the magnitude of the effects would enhance the clarity and informativeness of our findings. In response, we have revised the relevant sections of the manuscript to include quantitative descriptions of the effect sizes. The specific changes are highlighted in red font in the “Revised Manuscript with Track Changes” document. The main additions are as follows:

“The findings indicate that when the dependent variable is innovation efficiency, the coefficient for the core explanatory variable—digital intelligence level—equals 0.038, demonstrating a statistically significant positive correlation at the 1% level. This means that a 1% increase in the digital intelligence level is associated with an approximate 0.038% increase in innovation efficiency.” **The specific changes are highlighted in red font on page 25 of the “Revised Manuscript with Track Changes” document.**

“When the dependent variable is innovation quality, the coefficient for the core explanatory variable—the digital intelligence index—is 0.125 and statistically significant at the 1% level. This result indicates that a 1% increase in the digital intelligence index is associated with an approximate 0.125% increase in innovation quality, underscoring the significant positive impact of digital intelligence on firms’ innovation quality. More specifically, cloud computing, artificial intelligence, and digital technology application levels are all positively correlated with the innovation quality of SRDI enterprises at the 1% significance level. In contrast, the levels of big data and blockchain technology are not significantly associated with innovation quality, suggesting that, at this stage, the main drivers for improving innovation quality among SRDI enterprises are the adoption of cloud computing, artificial intelligence, and digital technology applications.” **The specific changes are highlighted in red font on page 25 to 26 of the “Revised Manuscript with Track Changes” document.**

We hope these revisions address the reviewer’s concern and improve the clarity and practical significance of our results. Thank you again for your valuable feedback.

8. **Reviewer #3 Comment on Discussion:**

Try to reduce repetition in sections discussing China’s innovation ecosystem and tech trends. Streamline for clarity.

Emphasize the unique value of intelligent technologies compared to datafication and digitization more concisely.

**Response:**

Try to reduce repetition in sections discussing China’s innovation ecosystem and tech trends. Streamline for clarity：

We thank the reviewer for the helpful suggestion to better contextualize our findings within broader global trends in digital intelligence adoption. We acknowledge that the original manuscript primarily presented basic regression results without delving into the implications and connections to real-world trends. To address this, we have expanded the discussion section to include a deeper exploration of the global digital transformation trend, particularly focusing on the adoption of digital intelligence technologies and their role in driving innovation. Specifically, we have consolidated the discussion as follows:

“Similarly, in China, the adoption of digital intelligence—including artificial intelligence and data-driven technologies—has accelerated innovation within specialized enterprises. For example, recent advancements such as DeepSeek’s R1 model demonstrate that algorithmic optimization can reduce reliance on costly high-performance chips, highlighting the potential of AI to lower infrastructure barriers.” **The specific changes are highlighted in red font on the page 26 of the “Revised Manuscript with Track Changes” document.**

Emphasize the unique value of intelligent technologies compared to datafication and digitization more concisely:

We sincerely appreciate the reviewer’s insightful suggestion to more clearly and concisely highlight the unique value of intelligent technologies relative to datafication and digitization. In response, we have carefully revised the relevant section in the manuscript to better articulate this distinction. The revised text now reads:

“Compared to datafication and digitization, intelligent technologies such as AI and machine learning uniquely drive innovation. They enable predictive analytics, adaptive decision-making, and automated optimization, which go beyond improving efficiency and empower firms to respond dynamically to market changes.” **The specific changes are highlighted in red font on the page 26 of the “Revised Manuscript with Track Changes” document.**

We hope that these additions help contextualize our research findings within the broader global trends and enhance the robustness of our study.

9. **Reviewer #3 Comment on Conclusion:**

Briefly outline future research directions (e.g., international comparisons, long-term digital effects).
 Add one sentence showing how SMEs can use digital tools for long-term growth, based on your findings.

**Response:**

Briefly outline future research directions (e.g., international comparisons, long-term digital effects).

We sincerely appreciate the reviewer’s insightful suggestion regarding the articulation of future research directions. In response to your comment, we have carefully revised the Conclusion section to more explicitly and thoroughly outline areas for future investigation.

First, we recognize that our study’s focus on SRDI enterprises in China may limit the broader applicability of our findings. To address this limitation, the revised manuscript now emphasizes the value of conducting international comparative studies in future research. We specifically suggest that future studies could analyze similar types of enterprises in different national contexts—such as Germany’s “hidden champions” or niche firms in the United States and Japan—in order to better understand how varying institutional and economic environments influence the relationship between digital intelligence adoption and innovation outcomes. We believe that this direction will greatly enhance the generalizability and policy relevance of subsequent research in this area. The revised future research now reads as follows:

“Future research could address this by expanding the analysis to similar enterprises in other countries, such as Germany’s “hidden champions” or niche firms in the United States and Japan. This would allow for an examination of how different institutional and economic contexts influence the relationship between digital intelligence and innovation.” **The specific changes are highlighted in red font on the page 40 of the “Revised Manuscript with Track Changes” document.**

Second, we have incorporated a more detailed discussion on the importance of examining the long-term effects of digital intelligence technologies. Given that our analysis is restricted to the period from 2014 to 2022, we acknowledge the need for future research to assess whether the observed positive effects of digital intelligence on innovation performance are sustainable over time. We further suggest that future studies investigate how the adoption of digital technologies shapes organizational dynamics and adaptation processes in the long run, as well as the potential new challenges and opportunities that may emerge as firms deepen their digital transformation. The revised future research now reads as follows:

“Therefore, future research could further explore the long-term effects of digital intelligence technologies on innovation performance, such as whether their impact is sustainable, how digital adoption shapes organizational dynamics over time, and what new challenges or opportunities may arise as enterprises deepen their digital transformation.” **The specific changes are highlighted in red font on the page 41 of the “Revised Manuscript with Track Changes” document.**

Add one sentence showing how SMEs can use digital tools for long-term growth, based on your findings.

Thank you very much for this valuable suggestion. We acknowledge that, in the original manuscript, our discussion of how SMEs could leverage digital intelligence technologies for long-term growth was relatively brief and lacked specific operational guidance. In response to your comment, we have carefully revised the Conclusion section to include a more explicit and actionable statement reflecting our empirical findings.The specific addition is as follows:

“In summary, SMEs can achieve long-term growth by prioritizing the adoption of easily deployable digital intelligence technologies, such as cloud computing and artificial intelligence, while adopting big data and blockchain solutions according to their specific development needs.” **The specific changes are highlighted in red font on the page 39 of the “Revised Manuscript with Track Changes” document.**

We hope this modification addresses the reviewer’s concern and enhances the clarity of our manuscript. Thank you again for your valuable feedback.

We have made these revisions to the best of our ability in accordance with your valuable suggestions. However, we recognize that our modifications may still fall short of your expectations. If you have any further comments or additional suggestions for improvement, please do not hesitate to let us know. We would be very grateful for your continued guidance and are more than willing to make any further revisions necessary to strengthen the manuscript.

Thank you once again for your time and consideration. We look forward to your feedback.

Best regards,

All authors

**Response submitted with Revision(Manuscript ID: PONE-D-24-53233)**

Dear Editor Ahmad,

We sincerely appreciate your review of our manuscript titled “The Impact of Digital Intelligence on Innovation Performance: Evidence from Specialized, Refined, Differential, and Innovative (SRDI) enterprises” (**PONE-D-24-53233**) and your valuable feedback. We are grateful for the thoughtful comments provided by the reviewer, and we have made comprehensive revisions in response to all the feedback.

Below are our detailed responses to each comment.

**Dear Dr. Muhammad Imran,**

We would like to begin by sincerely thanking you for your valuable and insightful comments on our manuscript. Your feedback has been instrumental in improving the quality of our paper, and we have made several revisions and additions in response to your suggestions. We believe these changes have enhanced the scientific rigor and applicability of our study. Below, we outline the specific improvements made based on your comments.

**Reviewer #1 General comments:**

**The study is limited to SMEs in China, which restricts its applicability to other countries or contexts. While the findings are robust, additional cross-regional comparisons or global contextualization could broaden the scope.**

**While quantitative methods are rigorously applied, the study lacks qualitative insights (e.g., interviews or case studies) to deepen the understanding of digital intelligence's real-world implications for SMEs.**

**Innovation performance is largely measured through patents and financial data, which may not fully capture softer innovation outputs, such as organizational learning or process improvements.**

**While the heterogeneity analysis identifies the stronger effects of digital intelligence on non-state-owned enterprises, this observation is not sufficiently explored in the discussion section.**

**Response:**

First, we would like to thank Dr. Muhammad Imran for his valuable insights. In response to the reviewer’s comments, we have made several revisions and additions to enhance the scientific rigor and applicability of the paper. The specific improvements are as follows:

First, although the study focuses on China’s SRDI enterprises, the concept of SRDI enterprises is derived from Germany’s Hidden Champions and bears many similarities with the concept of niche enterprises in the United States and Japan. Therefore, we believe that although the paper is primarily focused on China, it still has international relevance. Furthermore, China, like other countries (e.g., the United States, Japan, and Germany), places a strong emphasis on digital technologies and the development of innovative SMEs, which makes the conclusions of this study potentially beneficial to SMEs in different national and policy contexts. Second, regarding the measurement of innovation performance, the paper primarily uses patents and financial data as key indicators. We acknowledge that this approach does not fully capture softer innovation outcomes (e.g., organizational learning and process improvements). We have clearly stated this limitation in the manuscript and pointed out that future research could consider incorporating additional measures of softer innovation to more comprehensively assess innovation performance. Finally, in the heterogeneity analysis section, we have expanded our discussion on the impact of digital intelligence on non-state-owned enterprises and provided a more in-depth analysis, incorporating practical examples to further illustrate the practical implications of our findings.

10. **Reviewer #1 Comment on Abstract:**

**The abstract clearly presents the study’s objectives, methods, and key findings. However, it could briefly mention the practical implications to engage a broader audience.**

**Response:**

We sincerely appreciate the reviewer’s insightful suggestion to incorporate practical implications in the abstract. In response, we carefully considered how best to highlight the practical relevance of our study for a wider audience. While we initially explored various ways to integrate practical implications throughout the abstract, we found that due to the technical nature of the research and its focus on the theoretical mechanisms of digital intelligence and financing barriers, a concise statement would be the most effective way to maintain clarity and focus. Therefore, we have added the following statement to the revised abstract:

“These findings provide valuable insights for policymakers and enterprise decision-makers on leveraging digital intelligence to overcome financing barriers and foster innovation in small and SMEs”.

We believe this addition helps bridge the gap between theoretical findings and practical applications while maintaining the abstract’s primary focus. We hope this approach addresses the reviewer’s concerns while preserving the integrity of our research.

Additionally, in light of the evolving understanding of the concept of Specialized, Refined, Differential, and Innovative (SRDI) enterprises among Chinese scholars, we have updated the terminology to align with the current academic discourse. Furthermore, we have refined the abstract’s wording to enhance its clarity and coherence. **The specific changes are highlighted in red font on the page second of the “Revised Manuscript with Track Changes” document.**

We hope this revision effectively addresses the reviewer’s concern and enhances the broader applicability of our study.

11. **Reviewer #1 Comment on Introduction:**

The introduction effectively outlines the problem and significance of the study. To enhance clarity, a more detailed explanation of the novelty of the "Specialized, Fined, Peculiar, and Innovative" framework would be beneficial.

**Response:**

We would like to express our sincere gratitude to Reviewer for the valuable feedback and constructive suggestions. In response to the reviewer’s comment on enhancing the explanation of the novelty of the SRDI enterprises framework in the introduction, we have expanded the discussion on the specific attributes of SRDI enterprises based on relevant literature and official documents.We have provided a more detailed explanation of the four key components of SRDI enterprises as follows:

**Specialized**: Focusing on core business areas to enhance capabilities in specialized production, services, and collaboration. These enterprises provide components, parts, supporting products, and services to large enterprises, major projects, and industrial chains.

**Refined**: Emphasizing refined production, management, and services. These enterprises gain an advantage in niche markets by offering high-quality products and services that are highly regarded for their cost-effectiveness and superior quality.

**Differential**: Leveraging unique resources to promote traditional craftsmanship and regional culture. By adopting distinctive techniques, technologies, formulas, or raw materials, they produce products with local or enterprise-specific characteristics.

**Innovative**: Engaging in technological, management, and business model innovations to cultivate new growth areas and create new competitive advantages.

We would like to clarify that our initial intention was to provide a comprehensive explanation of SRDI enterprises for both you and the readers. However, we also realized that offering a detailed description of SRDI enterprises resulted in an excessive manuscript length. Therefore, to enhance the focus and coherence of our manuscript, we have streamlined the discussion by retaining only the key characteristics of SRDI enterprises that are directly relevant to our research. This revision not only improves readability but also ensures that our theoretical framework remains concise and well-aligned with the study’s objectives. **The specific changes are highlighted in red font on the page three of the “Revised Manuscript with Track Changes” document.**

This enhanced explanation aims to clarify the unique characteristics of SRDI enterprises and provide readers with a deeper understanding of their essential features. We hope these revisions help strengthen the manuscript's clarity and readability.

12. **Reviewer #1 Comment on Literature Review:**

The review provides a comprehensive summary of related studies but could include a deeper critique of existing gaps to highlight the study’s contribution.

**Response:**

We sincerely appreciate the reviewer’s insightful suggestion to enhance the critique of existing literature. In response to this, we have expanded the discussion on the gaps in the current literature, particularly in the context of China’s innovation landscape. Specifically, we highlight the persistent issues at both the micro and macro levels, such as strategic innovation driven by quantity rather than quality, and the prevalent phenomenon of “quantity-driven, quality-deficient” innovation. We also note that existing studies on patents tend to emphasize quantity over quality, which may introduce biases in the interpretation of research findings.

To provide further context, it is important to note that, since the post-economic-crisis era, China has created what is often referred to as the “China Miracle” in innovation, primarily driven by patent growth. According to the 2023 World Intellectual Property Indicators report by the World Intellectual Property Organization (WIPO), China has been the world leader in patent filings for five consecutive years, with 1.64 million invention patents filed in 2023 alone. Among these, corporate patent filings accounted for 1.13 million, ranking China 11th globally in terms of enterprise innovation. However, this rapid patent growth has also exposed significant issues, including low rates of commercialization and industrialization of research outcomes, as well as an increasing share of non-invention patents. These factors indicate that, although the quantity of innovation output has grown explosively, the quality of innovation may not have kept pace.

While we did not include these specific details in the manuscript due to space limitations, they were instrumental in shaping our critique of existing research and informing the revisions made to our paper. We hope that these additions further emphasize the contribution of our study by addressing these critical gaps in the literature. **The specific changes are highlighted in red font on the page ten of the “Revised Manuscript with Track Changes” document.**

We hope these revisions effectively highlight the contribution of our study by addressing these critical gaps in the literature.

13. **Reviewer #1 Comment on Methodology:**

The methodology is robust, but additional justification for selecting certain control variables (e.g., asset turnover ratio) would strengthen this section.

**Response:**

We thank the reviewer for the valuable suggestion to provide additional justification for the selection of certain control variables. In response to this, we have strengthened the explanation for the inclusion of these variables in our analysis. Specifically, we now provide a more detailed rationale for each control variable used in the study:

**Ownership Concentration (Top1)**: measured by the largest shareholder’s share ratio, reflects the influence of ownership structure on innovation performance. Greater concentration can centralize decision-making, shaping strategic direction, innovation priorities, and resource allocation.

**Board Size (Board)**: indicated by the number of sitting directors, is considered as it may reflect the firm’s governance quality. A larger board may provide diverse perspectives, potentially fostering a more innovation-driven environment. However, excessively large boards could introduce coordination challenges that might hinder timely and effective decision-making.

**Market Competition (HHI)**: measured by the Herfindahl-Hirschman Index of the industry, controls for competitive pressures on innovation. Higher market concentration (higher HHI) may reduce competition, weakening innovation incentives, while lower concentration intensifies competition, fostering innovation.

**Asset Turnover Ratio (ATO)**: calculated as the ratio of net operating revenue to average total assets, reflects how effectively a firm utilizes its assets to generate revenue. A higher ATO suggests better asset use, enabling greater resource allocation for innovation. Firms with higher turnover may also be more agile in deploying resources for technological advancements.

**Operating Cycle (Cycle)**: which refers to the time taken from acquiring inventory to selling it and recovering cash, is included to control for the firm’s operational efficiency. Firms with shorter cycles manage working capital better, enabling more financial resources for innovation.

**Operating Cash Flow (OCF)**: represented by the net cash flow generated from operating activities, controls for the financial health of the firm. Strong OCF reflects the firm’s ability to generate internal funds, reducing reliance on external financing and supporting stable innovation investments.

**Return on Assets (ROA)**: measured by the ratio of earnings before interest and taxes to average total assets, is included to account for the firm’s overall financial performance and efficiency. A higher ROA indicates better profitability and asset management, enhancing the firm’s ability to fund and sustain innovation.

After careful revision, we realized that providing detailed explanations of the control variables within the main text might make the paper overly lengthy and distract from the primary discussion. To maintain clarity and conciseness, we have streamlined this section while ensuring that all necessary details remain accessible to readers. Although we have omitted the detailed descriptions from the main text, the rationale and selection criteria for the control variables are still rigorously justified in the methodology section. We believe this approach enhances the manuscript’s readability while maintaining its academic rigor. **The specific changes are highlighted in red font on the page nineteen the “Revised Manuscript with Track Changes” document.**

By providing clear justifications for each of the control variables, we hope to strengthen the robustness of the variable selection and enhance the overall credibility of our methodology.

14. **Reviewer #1 Comment on Results and Discussion:**

The results are well-presented and supported by appropriate statistical analysis. However, the discussion could better contextualize the findings in relation to broader global trends in digital intelligence adoption.

**Response:**

We thank the reviewer for the helpful suggestion to better contextualize our findings within broader global trends in digital intelligence adoption. We acknowledge that the original manuscript primarily presented basic regression results without delving into the implications and connections to real-world trends. To address this, we have expanded the discussion section to include a deeper exploration of the global digital transformation trend, particularly focusing on the adoption of digital intelligence technologies and their role in driving innovation. Specifically, we have added the following content:

The ongoing global digital transformation trend increasingly recognizes the application of digital intelligence technologies as a key driver of innovation. In particular, SMEs in leading economies such as the United States, Germany, and Japan have been rapidly adopting digital technologies to enhance their innovation capabilities. National initiatives such as Germany’s “Industry 4.0” and Japan’s “Society 5.0” underscore the integration of smart technologies across both manufacturing and service sectors. Similarly, in China, the adoption of digital intelligence—encompassing artificial intelligence and data-driven technologies—has played a pivotal role in accelerating innovation within specialized enterprises. A prime example is the recently launched R1 model by DeepSeek, which has surpassed OpenAI in several tests and challenged Nvidia’s “computing power myth”. The R1 model demonstrates that, through algorithmic optimization, 60% of global AI computing power can be supported by mid-range chips, thus eliminating the need for costly high-performance chips. This breakthrough highlights AI’s transformative potential in reducing dependence on expensive infrastructure, while also underscoring China’s growing influence in the digital intelligence arena.

In this context, we emphasize that smart technologies like AI and machine learning have a more significant impact on innovation than other digital intelligence factors such as digitalization or data-driven technologies. While the latter are essential for data collection and processing, their role in innovation is often limited to enhancing operational efficiency. In contrast, smart technologies utilize advanced algorithms to not only analyze data but also to predict trends, optimize operations, and adapt to market changes. This predictive and adaptive capability accelerates and enhances innovation, distinguishing smart technologies from other digital tools.

Additionally, we reference a 2024 report by the Oxford Economics Institute and Cognizant Impact, which predicts that over the next decade, generative artificial intelligence will increase U.S. productivity by 1.7% to 3.5%, contributing to annual GDP growth ranging from $477 billion to $1 trillion. This provides further evidence of the profound impact that digital intelligence technologies, especially AI, are poised to have on global innovation and productivity. **The specific changes are highlighted in red font on the page twenty-five to twenty-six of the “Revised Manuscript with Track Changes” document.**

We hope that these additions help contextualize our research findings within the broader global trends and enhance the robustness of our study.

15. **Reviewer #1 Comment on Conclusion:**

The conclusion summarizes the findings well but should emphasize future research directions, such as longitudinal studies or comparisons across different industries.

**Response:**

We thank the reviewer for the constructive suggestion to emphasize future research directions in the conclusion. We acknowledge that the original manuscript did not sufficiently address this aspect. To remedy this, we have added the following discussion on future research directions:

In future research, we plan to investigate SMEs similar to the Specialized, Refined, Differential, and Innovative enterprises, such as Germany’s “Hidden Champions”, and niche firms in the United States and Japan. These enterprises share developmental characteristics with China’s specialized enterprises. Given the global trend of digital intelligence development, examining how innovation-driven SMEs can effectively leverage digital technologies to drive high-quality innovation will be a key direction for future studies. Additionally, while our study differentiates between manufacturing and non-manufacturing industries, we see an opportunity to further refine this analysis by exploring the impact of digital intelligence technologies on strategic emerging industries within the manufacturing sector. This deeper exploration could yield valuable insights into the sector-specific application of digital transformation technologies. **The specific changes are highlighted in red font on the page thirty-nine of the “Revised Manuscript with Track Changes” document.**

By including these future research directions, we aim to strengthen the manuscript’s structure and provide a more comprehensive outlook on the ongoing and future trends in digital intelligence adoption and innovation.

We sincerely appreciate Dr. Muhammad Imran’s valuable comments, which have been incredibly helpful in improving the quality of our manuscript. Although we have made every effort to address the suggestions and revisions to the best of our ability, we understand that our changes may still not fully meet the reviewer’s expectations. We would be truly grateful for any further comments or guidance you may have, as we are committed to improving the manuscript to the highest standard.

Thank you once again for your time and valuable input.

**Dear Dr. Irma Nur Afiah,**

We would like to sincerely thank you for your thoughtful and detailed feedback on our manuscript titled The Integration of Digital and Intelligent Technologies for the Innovative Growth of Specialized, Fine, Peculiar, and Innovative SMEs in China. Your comments and suggestions are highly appreciated and have provided us with valuable insights to improve the clarity, depth, and contribution of our research.

Below, we have carefully addressed each of the points raised in your review. We hope that the revisions we have made will meet your expectations and improve the quality of the manuscript.

1. **Reviewer #2 Comment on Introduction:**

The introduction provides a good overview of the topic, but it could benefit from more detailed context regarding the importance of SMEs in China’s economy. Elaborating on the specific challenges faced by SMEs in this sector, particularly in terms of innovation and financing, would help set the stage for your study.

Consider explaining more about the term “Specialized, Fine, Peculiar, and Innovative SMEs” and why this specific category was chosen for the research.

**Response:**

we have enhanced the introduction by addressing the following points: First, we have clarified the concept of Specialized, Refined, Differential, and Innovative(SRDI) enterprises by reviewing relevant literature and official documents. We have provided a more detailed explanation of the four key components of SRDI enterprises as follows: **Specialized**: Focusing on core business areas to enhance capabilities in specialized production, services, and collaboration. These enterprises provide components, parts, supporting products, and services to large enterprises, major projects, and industrial chains. **Refined**: Emphasizing refined production, management, and services. These enterprises gain an advantage in niche markets by offering high-quality products and services that are highly regarded for their cost-effectiveness and superior quality. **Differential**: Leveraging unique resources to promote traditional craftsmanship and regional culture. By adopting distinctive techniques, technologies, formulas, or raw materials, they produce products with local or enterprise-specific characteristics. **Innovative**: Engaging in technological, management, and business model innovations to cultivate new growth areas and create new competitive advantages. While we considered expanding on each of these components further, we are mindful of ensuring that the manuscript remains focused and concise. We chose to provide a clear and concise overview of the key characteristics of SRDI enterprises to ensure that readers gain a comprehensive understanding without overwhelming them with excessive detail. We believe that this approach allows us to maintain the clarity and academic rigor of the manuscript while avoiding unnecessary complexity.

Second, we have provided a more comprehensive discussion of the role of SMEs in China’s economy, highlighting their significant contributions to innovation and GDP, while also identifying the key challenges they face, particularly in terms of financing. Finally, we have elaborated on the reasons for selecting SRDI enterprises as the focus of this study. We emphasize that SRDI enterprises play a key role in China’s manufacturing powerhouse strategy and are central to addressing critical technological bottlenecks. By focusing on these enterprises, we aim to better explore the role of digital intelligence technologies in driving innovation within SMEs, filling the existing gap in research on the mechanisms of digital intelligence empowerment. **The specific changes are highlighted in red font on the page three to four of the “Revised Manuscript with Track Changes” document.**

We hope these revisions help clarify the objectives of our study and improve the overall quality of the manuscript.

2. **Reviewer #2 Comment on Methodology:**

The description of the empirical investigation is informative, but additional details regarding the data collection process (e.g., the criteria for selecting SMEs, sample size, and potential biases in the dataset) would strengthen the methodology section. Additionally, it would be helpful to discuss any challenges or limitations in the data, particularly concerning missing data or data quality.

**Response:**

We would like to express our sincere gratitude for your valuable feedback on our manuscript. Indeed, we acknowledge that the original version of our manuscript did not provide sufficient details regarding the data processing methods. Following your suggestions, we have revised the methodology section to include more specific information on the data collection and treatment process. Specifically, we have clarified the criteria for selecting SMEs in our study. As the Ministry of Industry and Information Technology of China has published six batches of SRDI enterprises to date, we made sure that our research sample includes enterprises from all six batches. This ensures that the study comprehensively covers SRDI enterprises across different phases.

Additionally, we further elaborated on the data screening process. First, we excluded enterprises with severe missing data. Secondly, due to the limited number of SRDI enterprises in underdeveloped regions such as Tibet, we excluded enterprises from these areas to avoid potential biases related to regional disparities. For the remaining missing data, we employed interpolation techniques to estimate the missing values. After these adjustments, the final sample consisted of 1,100 enterprises, representing all six batches of SRDI enterprises, with a total of 9,990 enterprise-year observations. **The specific changes are highlighted in red font on the page thirteen to fourteen of the “Revised Manuscript with Track Changes” document.**

We hope that these revisions clarify the methodology and address the concerns regarding data selection and quality. We are grateful for your valuable feedback, which has significantly strengthened the rigor of our study.

3. **Reviewer #2 Comment on Key Findings and Analysis:**

Your findings that digital intelligence, particularly intelligent technologies, improve the innovation performance of SMEs are in line with contemporary research and provide valuable insights. I would encourage you to explore in more depth why intelligent technologies had a stronger impact compared to other aspects of digital intelligence (such as datafication or digitization).

The positive impact of digital intelligence on alleviating financing constraints is significant. Could you provide more specific examples of how these SMEs utilized their digital capabilities to overcome these constraints? Furthermore, why do you think non-state-owned enterprises in particular showed more pronounced benefits from digital intelligence adoption? A deeper exploration of these differences could enrich the analysis.

**Response:**

We sincerely appreciate your thoughtful feedback on our findings and discussion. Your insightful suggestions have been truly helpful in identifying the limitations of our original manuscript and refining our analysis. Thank you for your time and effort in improving the clarity and depth of our work.

Thank you for highlighting the need to further explore why intelligent technologies, particularly AI and machine learning, exhibit a stronger impact on innovation than other digital intelligence components such as datafication and digitization. We have revised our discussion to provide a more comprehensive explanation. While datafication and digitization establish a foundation by enabling efficient data collection, storage, and processing, their direct role in driving innovation is often constrained to operational efficiency improvements. In contrast, intelligent technologies not only analyze data but also incorporate advanced algorithmic capabilities to predict trends, optimize processes, and dynamically adapt to changing market conditions. This predictive and adaptive nature allows AI and machine learning to accelerate the innovation cycle and generate more substantial impacts. To illustrate this point, we have included an example of the DeepSeek R1 model, which optimizes AI computing efficiency through advanced algorithms, reducing reliance on high-performance chips. This case demonstrates how intelligent technologies can significantly reshape technological trajectories by enhancing efficiency while reducing infrastructure constraints. Additionally, we have incorporated findings from a 2024 Oxford Economics Institute and Cognizant Impact report, which estimates that generative AI could increase U.S. productivity by 1.7% to 3.5% over the next decade, contributing to an annual GDP growth of $477 billion to $1 trillion. These revisions help clarify the distinct advantages of intelligent technologies over traditional digital intelligence factors. **The specific changes are highlighted in red font on the page twenty-five to twenty-six of the “Revised Manuscript with Track Changes” document.**

We appreciate your suggestion to provide more concrete examples of how SMEs leverage digital intelligence to overcome financing constraints. In response, we have expanded our discussion with specific cases from both China and the United States. In China, according to a 2023 report by the China Banking and Insurance Regulatory Commission (CBIRC), SMEs face a financing gap exceeding 13 trillion RMB, which traditional banking institutions struggle to address. In response, digital lending platforms such as Ant Group and JD Digits have pioneered AI-driven credit assessment models. These platforms use real-time business data, rather than conventional collateral-based evaluations, to provide faster and more flexible financing solutions. This enables SMEs to access much-needed capital, ensuring continued investment in R&D and innovation. Similarly, in the United States, the Federal Reserve Bank of New York reports an estimated $100 billion credit gap for SMEs, primarily due to traditional banks’ reluctance to provide small business loans. Technology-driven lenders such as Kabbage and OnDeck address this issue by leveraging big data analytics and digital platforms to assess creditworthiness in real time. These platforms enable SMEs to secure financing more efficiently, allowing them to maintain a steady trajectory of innovation and technological development despite conventional financial constraints. However, after careful consideration, we decided to focus on the Chinese example in the main text, for the following reasons:

Relevance to the Study’s Core Context: Since the study primarily focuses on the Chinese market and its unique challenges, we felt that the case of Ant Group and JD Digits provided a more relevant and specific illustration of how digital intelligence can alleviate financing constraints within China’s regulatory and financial environment. Adding the U.S. example, while valuable, could shift the focus away from the primary regional context of the study.

Maintaining Conciseness and Focus: In the interest of maintaining the paper’s conciseness and clarity, we opted to limit the number of examples included in the main text. Our goal was to ensure that the core argument is clear and well-supported without introducing unnecessary complexity or length. We believe the Chinese example sufficiently supports the thesis and provides the required depth of analysis.

Avoiding Redundancy: The key points raised by both the Chinese and U.S. examples were similar, especially regarding the role of digital platforms in facilitating SME financing. Including both examples could create redundancy, which might detract from the paper’s overall focus and impact. Therefore, we chose to retain the example most directly relevant to the study.

These additions provide a more concrete foundation for our argument on how digital intelligence facilitates SME financing, reinforcing the practical significance of our findings. **The specific changes are highlighted in red font on the page twenty-eight of the “Revised Manuscript with Track Changes” document.**

Thank you for raising this important question regarding why non-state-owned enterprises (NSOEs) in China derive greater benefits from digital intelligence adoption compared to state-owned enterprises (SOEs). We have expanded our discussion to further clarify this distinction. NSOEs operate in a highly competitive and dynamic market environment, requiring continuous technological adaptation to maintain their market position. This competitive pressure drives NSOEs to proactively integrate AI, big data, and blockchain technologies to enhance operational efficiency and innovation output. In contrast, SOEs often prioritize economic stability and policy alignment over technological agility. Institutional inertia and bureaucratic decision-making processes can slow down the adoption of emerging digital technologies, limiting their capacity to fully leverage digital intelligence for innovation. We have also cited empirical findings that highlight these differences, reinforcing our argument that NSOEs, driven by market competition rather than policy mandates, exhibit a greater urgency and flexibility in adopting digital intelligence technologies to sustain their innovation performance. **The specific changes are highlighted in red font on the page thirty-two to thirty-three of the “Revised Manuscript with Track Changes” document.**

We sincerely hope that our revisions have effectively addressed your concerns and further strengthened the clarity and rigor of our study. Thank you again for your valuable feedback and constructive suggestions.

4. **Reviewer #2 Comment on Implication and Contribution:**

The practical implications of your study for policymakers and SMEs are valuable. It would be beneficial to connect your findings to existing theories or frameworks on digital transformation and innovation management, especially in the context of emerging economies like China. Are there any new theoretical insights that your study contributes to the literature on digital intelligence and SME innovation?

Discussing how your findings could influence future business strategies for SMEs would add value. Specifically, how can SMEs in China leverage digital intelligence for long-term growth?

**Response:**

We would like to express our sincere gratitude for your constructive comments and insightful feedback. Your suggestions have significantly contributed to enhancing both the theoretical depth and practical relevance of our study.

In response to your suggestion, we have strengthened the theoretical foundation by explicitly linking our findings to Schumpeterian innovation theory. This connection underscores how digital intelligence not only enhances efficiency but also fosters breakthrough innovations in SRDI enterprises. Additionally, our study extends the resource-based view (RBV) by incorporating financing constraints as a moderating factor, which highlights how financial limitations impede firms from fully leveraging digital intelligence for innovation. This extension provides novel insights into digital transformation strategies for SMEs, especially in resource-constrained environments. Furthermore, by integrating an institutional perspective into our heterogeneity analysis, we demonstrate how regional, ownership, and industry-specific factors influence the relationship between digital intelligence and innovation. These contributions enrich the theoretical framework of digital transformation, offering a more nuanced understanding of its impact in the context of emerging economies. **The specific changes are highlighted in red font on the page five of the “Revised Manuscript with Track Changes” document.**

We have also expanded the discussion on how SMEs, particularly in China, can leverage digital intelligence for long-term growth. Specifically, we propose that SMEs accelerate the adoption of cloud computing and AI technologies, which can enhance decision-making, optimize operations, and drive innovation, all while minimizing the need for significant capital investments. Furthermore, we discuss how the use of blockchain and big data analytics can improve financing access for SMEs by enhancing transparency, reducing information asymmetry, and improving creditworthiness. These aspects are particularly pertinent in China’s financial landscape, where access to capital remains a persistent challenge for SMEs. Additionally, we emphasize the potential of digital technologies in facilitating international expansion. By utilizing cross-border e-commerce and digital marketing platforms, SMEs can overcome geographic constraints and strengthen their competitive positioning in global markets. These revisions reinforce the connection between our findings and existing theoretical frameworks, while also offering actionable insights for SMEs navigating digital transformation in emerging markets. **The specific changes are highlighted in red font on the page thirty-eight to thirty-nine of the “Revised Manuscript with Track Changes” document.**

We are grateful for your valuable feedback, which has significantly improved the clarity and depth of our study, and we look forward to any further suggestions you may have.

5. **Reviewer #2 Comment on Limitation and Future Research:**

While your study offers valuable insights, it would be beneficial to address the limitations of your research more explicitly. For example, are there external factors, such as policy changes or economic conditions, that could have impacted the SMEs during your study period?

I suggest that future research explore the long-term effects of digital intelligence on SME innovation, and examine how digital transformation interacts with other factors, such as government policy and industry-specific trends.

**Response:**

We sincerely appreciate your insightful feedback, which has significantly contributed to refining our analysis and enhancing the clarity of our discussion. We acknowledge that our original manuscript did not sufficiently elaborate on the study’s limitations, and we have now incorporated a more explicit discussion in the revised version.

During the study period, SRDI enterprises encountered several external challenges that likely influenced their innovation activities. For instance, China’s supply-side structural reforms led to rising raw material costs in key manufacturing sectors, intensifying financial pressures on SMEs. The COVID-19 pandemic further exacerbated these difficulties by disrupting supply chains, particularly in the semiconductor industry—delaying product development for technology-driven firms. Additionally, while the 2022 VAT rebate policy was introduced to alleviate financial burdens, its uneven implementation restricted access for certain non-state-owned enterprises, potentially limiting their ability to leverage digital intelligence for innovation.

A concrete example can be observed in Shandong Province, where the industrial transformation initiative launched in 2018 aimed to modernize traditional industries by promoting advanced manufacturing and new energy sectors. Although designed to drive industrial upgrading, this shift inadvertently led to a significant outflow of enterprises, with more than half of the province’s industrial cities experiencing business relocations to other regions, resulting in an estimated GDP decline of nearly 2 trillion RMB. Similar green development policies in other regions imposed additional constraints on manufacturing enterprises, further impacting their capacity to adopt digital intelligence technologies. However, after careful consideration, we chose not to include this example in the main text for the following reasons:

**Maintaining Focus on Core Research Theme**: The case of Shandong, although insightful, addresses the unintended consequences of green development policies, which we believe might divert the reader's attention from the central focus of how SMEs use digital intelligence to overcome financing constraints. Including this example could potentially shift the manuscript’s emphasis.

**Broader Context**: The challenges observed in Shandong are part of a broader phenomenon seen in multiple regions, and while relevant, they are somewhat tangential to the primary focus of our paper. We felt that the other examples already provided a clear and representative overview of the broader challenges SMEs face.

**Conciseness and Clarity**: Given the manuscript’s focus and length constraints, we aimed to present the most directly relevant cases to ensure clarity and conciseness. While the Shandong example is important, including it might add unnecessary complexity to the argument. We hope this explanation clarifies our reasoning.

At the same time, we have carefully considered your suggestions regarding future research directions. In this regard, future studies could explore the long-term effects of digital intelligence on SME innovation by examining the interaction between digital transformation and government policies, particularly the SRDI enterprises recognition policy in China. This policy, which aims to support high-tech, specialized SMEs, could serve as a critical factor in understanding how SMEs adopt digital technologies and innovate within the framework of government incentives and industry regulations. Furthermore, future research could investigate how digital transformation interacts with industry-specific trends, such as the shift towards green manufacturing or the adoption of emerging technologies in sectors like semiconductors. Such an approach would provide valuable insights into how SMEs can leverage digital intelligence to foster innovation across diverse industries and policy environments. However, due to space limitations, a more detailed discussion of these future research directions has not been included in the manuscript. **The specific changes are highlighted in red font on the page thirty-nine to forty of the “Revised Manuscript with Track Changes” document.**

We hope these revisions provide a more comprehensive discussion of the study’s limitations. Once again, we sincerely appreciate your constructive feedback.

6. **Reviewer #2 Comment on References:**

The references section could be enhanced by incorporating more recent studies on digital intelligence in SMEs, particularly those focusing on the Chinese context. Additionally, including references to works that explore government policies on digital transformation in China would further contextualize your findings.

**Response:**

Thank you for your insightful comments and suggestions regarding the references section of our manuscript. We appreciate your recommendation to incorporate more recent studies on digital intelligence in SMEs, particularly those focusing on the Chinese context, as well as works that explore government policies on digital transformation in China. Based on your feedback, we have revised the references section to better reflect the context of digital transformation in China.

In response to your suggestions, we have added three recent and relevant studies to our reference list to enhance the depth of our research. Firstly, we have included the work by Ma et al. (2024), which explores the role of resource orchestration in enabling SMEs to leverage digital opportunities. This study provides valuable insights into how traditional resources alone are insufficient for digital transformation, and highlights the necessity of digital platform capabilities for SMEs to effectively utilize digital opportunities. This is particularly relevant to our study, as it guides SMEs facing the challenge of “having resources but not knowing how to use them”. **The specific changes are highlighted in red font on the page six of the “Revised Manuscript with Track Changes” document.**

Secondly, we have referenced the article by Yao et al. (2024), which discusses how artificial intelligence can improve firm productivity by shifting the demand for labor from low-skilled to high-skilled workers. This study provides a crucial link between labor structure adjustment and firm productivity, directly aligning with the theme of technological transformation in our work. **The specific changes are highlighted in red font on the page seven of the “Revised Manuscript with Track Changes” document.**

Finally, we have incorporated the study by Liu et al. (2024), which investigates the impact of digital government initiatives on the quality upgrading of China’s export products. This article helps contextualize our findings within the broader framework of government policies on digital transformation in China, which is particularly relevant given the importance of government-driven digitalization in driving economic growth and trade development. **The specific changes are highlighted in red font on the page six of the “Revised Manuscript with Track Changes” document.**

In fact, there has been a growing body of research in China focused on digital intelligence in SMEs. We have carefully selected a few of the most representative studies to illustrate the current developments in digitalization in China. We believe that these additions significantly strengthen the theoretical grounding and practical implications of our research for the following reasons:

**Relevance to Current Literature**: The selected studies provide insights into the ongoing trends in digital transformation within Chinese SMEs, helping to position our research in the context of current developments. We believe these references demonstrate how our work aligns with the current discourse on digital intelligence in China.

**Enhancing Theoretical Depth**: By incorporating these studies, we aim to ground our research in recent and relevant literature. These references reinforce the theoretical foundation of our study, ensuring that it is supported by up-to-date academic perspectives on digital transformation in SMEs.

**Strengthening Practical Implications**: The selected studies also enhance the practical significance of our findings by providing real-world examples of how digital intelligence is being leveraged by SMEs in China. This helps to contextualize our research and highlight its relevance to SMEs seeking to overcome digital challenges.

Thank you once again for your constructive feedback. We look forward to your further comments and suggestions.

We sincerely appreciate the time and effort that the reviewers, Dr. Muhammad Imran and Dr. Irma Nur Afiah, have dedicated to evaluating our manuscript. Their insightful comments have significantly contributed to improving the quality and clarity of our work. We have carefully addressed all the concerns raised and incorporated the necessary revisions accordingly.

Throughout the revision process, we have made every effort to integrate the reviewers’ valuable suggestions while maintaining the methodological rigor of our study. Our primary goal has been to enhance the clarity and coherence of the manuscript, achieving a balanced improvement in academic value and policy relevance. To achieve this, we have refined the presentation of our methodology and findings, improving readability while preserving the depth and integrity of the study. We have also carefully structured the content to reduce any potential reading burden while maintaining the necessary details to support our conclusions.

We hope that the revised manuscript now meets the journal’s standards and aligns with the reviewers' expectations. Should there be any further suggestions or modifications required, we would be more than happy to address them.

**Additionally, regarding Figures 1 and 2, we have included them in the compressed file under Supporting Information. This ensures accessibility while keeping the main manuscript concise. We appreciate the reviewer’s attention to detail and hope this format aligns with the journal’s submission requirements.**

**Furthermore, we have supplemented the funding disclosure on page 44 with the following statement:**

**"This work was partially supported by the Shandong Social Science Foundation (No. 22CJJJ32)."**

Thank you once again for your time and consideration. We look forward to your feedback.

Best regards,

All authors
