# Supplementary material for: The impact of digital intelligence technologies on innovation performance: Evidence from specialized, refined, differential and innovative enterprises
Source: PLoS One. 2026 Feb 10;21(2):e0339567. doi: 10.1371/journal.pone.0339567 (PMC12890174; doi:10.1371/journal.pone.0339567)
Supplement: Peer review history

# Peer review: Decision Letter 0

**DOI:** 10.1371/journal.pone.0339567.r001

**Authors:** Yasir Ahmad

29 Jan 2025

Dear Dr. Zhang,

Please submit your revised manuscript by Mar 09 2025 11:59PM. If you will need more time than this to complete your revisions, please reply to this message or contact the journal office at plosone@plos.org . When you're ready to submit your revision, log on to https://www.editorialmanager.com/pone/ and select the 'Submissions Needing Revision' folder to locate your manuscript file.

- A rebuttal letter that responds to each point raised by the academic editor and reviewer(s). You should upload this letter as a separate file labeled 'Response to Reviewers'.
- A marked-up copy of your manuscript that highlights changes made to the original version. You should upload this as a separate file labeled 'Revised Manuscript with Track Changes'.
- An unmarked version of your revised paper without tracked changes. You should upload this as a separate file labeled 'Manuscript'.

If you would like to make changes to your financial disclosure, please include your updated statement in your cover letter. Guidelines for resubmitting your figure files are available below the reviewer comments at the end of this letter.

If applicable, we recommend that you deposit your laboratory protocols in protocols.io to enhance the reproducibility of your results. Protocols.io assigns your protocol its own identifier (DOI) so that it can be cited independently in the future. For instructions see: https://journals.plos.org/plosone/s/submission-guidelines#loc-laboratory-protocols . Additionally, PLOS ONE offers an option for publishing peer-reviewed Lab Protocol articles, which describe protocols hosted on protocols.io. Read more information on sharing protocols at https://plos.org/protocols?utm_medium=editorial-email&utm_source=authorletters&utm_campaign=protocols .

We look forward to receiving your revised manuscript.

Kind regards,

Yasir Ahmad

Academic Editor

PLOS ONE

When submitting your revision, we need you to address these additional requirements.

1. Please ensure that your manuscript meets PLOS ONE's style requirements, including those for file naming. The PLOS ONE style templates can be found at https://journals.plos.org/plosone/s/file?id=wjVg/PLOSOne_formatting_sample_main_body.pdf and https://journals.plos.org/plosone/s/file?id=ba62/PLOSOne_formatting_sample_title_authors_affiliations.pdf.

2. Please match your authorship list in your manuscript file and in the system.

3. In the online submission form, you indicated that [The patent data used in this study are publicly available and can be retrieved from the China National Intellectual Property Administration (CNIPA) database. The remaining datasets, including financial and other related variables, are available from the CSMAR (China Stock Market & Accounting Research) database. Access to the CSMAR database requires a valid subscription or institutional access, and data can be obtained by researchers with the appropriate credentials.

Additionally, the data generated through calculations and analysis in this study, such as the results from Data Envelopment Analysis (DEA) and Principal Component Analysis (PCA), are available from the corresponding author upon reasonable request.

If applicable, access to the data underlying the results of this study will be available after acceptance. Researchers can contact the corresponding author for further details and to request the data].

All PLOS journals now require all data underlying the findings described in their manuscript to be freely available to other researchers, either 1. In a public repository, 2. Within the manuscript itself, or 3. Uploaded as supplementary information.

This policy applies to all data except where public deposition would breach compliance with the protocol approved by your research ethics board. If your data cannot be made publicly available for ethical or legal reasons (e.g., public availability would compromise patient privacy), please explain your reasons on resubmission and your exemption request will be escalated for approval.

4. Please upload a copy of Figure 1 and 2, to which you refer in your text on page 20. If the figure is no longer to be included as part of the submission please remove all reference to it within the text.

[Note: HTML markup is below. Please do not edit.]

Reviewers' comments:

Reviewer's Responses to Questions

**Comments to the Author**

1. Is the manuscript technically sound, and do the data support the conclusions?

Reviewer #1: Yes

Reviewer #2: Yes

**********

2. Has the statistical analysis been performed appropriately and rigorously?

Reviewer #1: Yes

Reviewer #2: Yes

**********

3. Have the authors made all data underlying the findings in their manuscript fully available?

The PLOS Data policy

Reviewer #1: Yes

Reviewer #2: Yes

**********

4. Is the manuscript presented in an intelligible fashion and written in standard English?

Reviewer #1: No

Reviewer #2: Yes

**********

Reviewer #1: General comments

The study is limited to SMEs in China, which restricts its applicability to other countries or contexts. While the findings are robust, additional cross-regional comparisons or global contextualization could broaden the scope.

While quantitative methods are rigorously applied, the study lacks qualitative insights (e.g., interviews or case studies) to deepen the understanding of digital intelligence's real-world implications for SMEs.

Innovation performance is largely measured through patents and financial data, which may not fully capture softer innovation outputs, such as organizational learning or process improvements.

While the heterogeneity analysis identifies the stronger effects of digital intelligence on non-state-owned enterprises, this observation is not sufficiently explored in the discussion section.

Section wise comments

1. Abstract

• The abstract clearly presents the study’s objectives, methods, and key findings. However, it could briefly mention the practical implications to engage a broader audience.

2. Introduction

• The introduction effectively outlines the problem and significance of the study. To enhance clarity, a more detailed explanation of the novelty of the "Specialized, Fined, Peculiar, and Innovative" framework would be beneficial.

3. Literature Review

• The review provides a comprehensive summary of related studies but could include a deeper critique of existing gaps to highlight the study’s contribution.

4. Methodology

• The methodology is robust, but additional justification for selecting certain control variables (e.g., asset turnover ratio) would strengthen this section.

5. Results and Discussion

• The results are well-presented and supported by appropriate statistical analysis. However, the discussion could better contextualize the findings in relation to broader global trends in digital intelligence adoption.

6. Conclusion

• The conclusion summarizes the findings well but should emphasize future research directions, such as longitudinal studies or comparisons across different industries.

Reviewer #2: Dear Authors,

I have carefully reviewed your article titled The Integration of Digital and Intelligent Technologies for the Innovative Growth of Specialized, Fine, Peculiar, and Innovative SMEs in China. The study addresses a timely and important topic, exploring the impact of digital intelligence on the innovation performance of SMEs in China. Below are my detailed comments and suggestions to improve the clarity, depth, and contribution of your research.

1. Introduction

The introduction provides a good overview of the topic, but it could benefit from more detailed context regarding the importance of SMEs in China’s economy. Elaborating on the specific challenges faced by SMEs in this sector, particularly in terms of innovation and financing, would help set the stage for your study.

Consider explaining more about the term "Specialized, Fine, Peculiar, and Innovative SMEs" and why this specific category was chosen for the research.

2. Methodology

The description of the empirical investigation is informative, but additional details regarding the data collection process (e.g., the criteria for selecting SMEs, sample size, and potential biases in the dataset) would strengthen the methodology section. Additionally, it would be helpful to discuss any challenges or limitations in the data, particularly concerning missing data or data quality.

3. Key Findings and Analysis

Your findings that digital intelligence, particularly intelligent technologies, improve the innovation performance of SMEs are in line with contemporary research and provide valuable insights. I would encourage you to explore in more depth why intelligent technologies had a stronger impact compared to other aspects of digital intelligence (such as datafication or digitization).

The positive impact of digital intelligence on alleviating financing constraints is significant. Could you provide more specific examples of how these SMEs utilized their digital capabilities to overcome these constraints? Furthermore, why do you think non-state-owned enterprises in particular showed more pronounced benefits from digital intelligence adoption? A deeper exploration of these differences could enrich the analysis.

4. Implication and Contribution

The practical implications of your study for policymakers and SMEs are valuable. It would be beneficial to connect your findings to existing theories or frameworks on digital transformation and innovation management, especially in the context of emerging economies like China. Are there any new theoretical insights that your study contributes to the literature on digital intelligence and SME innovation?

Discussing how your findings could influence future business strategies for SMEs would add value. Specifically, how can SMEs in China leverage digital intelligence for long-term growth?

5. Limitation and Future Research

While your study offers valuable insights, it would be beneficial to address the limitations of your research more explicitly. For example, are there external factors, such as policy changes or economic conditions, that could have impacted the SMEs during your study period?

I suggest that future research explore the long-term effects of digital intelligence on SME innovation, and examine how digital transformation interacts with other factors, such as government policy and industry-specific trends.

6. References

The references section could be enhanced by incorporating more recent studies on digital intelligence in SMEs, particularly those focusing on the Chinese context. Additionally, including references to works that explore government policies on digital transformation in China would further contextualize your findings.

n summary, your study provides valuable insights into the role of digital intelligence in improving the innovation performance of SMEs in China. By expanding on some of the points mentioned above, such as the methodology, contextual background, and implications for practice, the overall impact of your paper can be strengthened.

Thank you for your valuable contribution to this field, and I look forward to reading the revised version of your manuscript.

**********

what does this mean? ). If published, this will include your full peer review and any attached files.

If you choose “no”, your identity will remain anonymous but your review may still be made public.

**Do you want your identity to be public for this peer review?** For information about this choice, including consent withdrawal, please see our Privacy Policy

Reviewer #1: **Yes:** Dr Muhammad Imran

Reviewer #2: **Yes:** Irma Nur Afiah

**********

[NOTE: If reviewer comments were submitted as an attachment file, they will be attached to this email and accessible via the submission site. Please log into your account, locate the manuscript record, and check for the action link "View Attachments". If this link does not appear, there are no attachment files.]

While revising your submission, please upload your figure files to the Preflight Analysis and Conversion Engine (PACE) digital diagnostic tool, https://pacev2.apexcovantage.com/ . PACE helps ensure that figures meet PLOS requirements. To use PACE, you must first register as a user. Registration is free. Then, login and navigate to the UPLOAD tab, where you will find detailed instructions on how to use the tool. If you encounter any issues or have any questions when using PACE, please email PLOS at figures@plos.org . Please note that Supporting Information files do not need this step.

# Author comment: Author response to Decision Letter 1

**DOI:** 10.1371/journal.pone.0339567.r002

13 Mar 2025

Dear Editor Ahmad,

We sincerely appreciate your review of our manuscript titled “The Impact of Digital Intelligence on Innovation Performance: Evidence from Specialized, Refined, Differential, and Innovative (SRDI) enterprises” (PONE-D-24-53233) and your valuable feedback. We are grateful for the thoughtful comments provided by the reviewer, and we have made comprehensive revisions in response to all the feedback.

Below are our detailed responses to each comment.

Dear Dr. Muhammad Imran,

We would like to begin by sincerely thanking you for your valuable and insightful comments on our manuscript. Your feedback has been instrumental in improving the quality of our paper, and we have made several revisions and additions in response to your suggestions. We believe these changes have enhanced the scientific rigor and applicability of our study. Below, we outline the specific improvements made based on your comments.

Reviewer #1 General comments:

The study is limited to SMEs in China, which restricts its applicability to other countries or contexts. While the findings are robust, additional cross-regional comparisons or global contextualization could broaden the scope.

While quantitative methods are rigorously applied, the study lacks qualitative insights (e.g., interviews or case studies) to deepen the understanding of digital intelligence's real-world implications for SMEs.

Innovation performance is largely measured through patents and financial data, which may not fully capture softer innovation outputs, such as organizational learning or process improvements.

While the heterogeneity analysis identifies the stronger effects of digital intelligence on non-state-owned enterprises, this observation is not sufficiently explored in the discussion section.

Response:

First, we would like to thank Dr. Muhammad Imran for his valuable insights. In response to the reviewer’s comments, we have made several revisions and additions to enhance the scientific rigor and applicability of the paper. The specific improvements are as follows:

First, although the study focuses on China’s SRDI enterprises, the concept of SRDI enterprises is derived from Germany’s Hidden Champions and bears many similarities with the concept of niche enterprises in the United States and Japan. Therefore, we believe that although the paper is primarily focused on China, it still has international relevance. Furthermore, China, like other countries (e.g., the United States, Japan, and Germany), places a strong emphasis on digital technologies and the development of innovative SMEs, which makes the conclusions of this study potentially beneficial to SMEs in different national and policy contexts. Second, regarding the measurement of innovation performance, the paper primarily uses patents and financial data as key indicators. We acknowledge that this approach does not fully capture softer innovation outcomes (e.g., organizational learning and process improvements). We have clearly stated this limitation in the manuscript and pointed out that future research could consider incorporating additional measures of softer innovation to more comprehensively assess innovation performance. Finally, in the heterogeneity analysis section, we have expanded our discussion on the impact of digital intelligence on non-state-owned enterprises and provided a more in-depth analysis, incorporating practical examples to further illustrate the practical implications of our findings.

1.Reviewer #1 Comment on Abstract:

The abstract clearly presents the study’s objectives, methods, and key findings. However, it could briefly mention the practical implications to engage a broader audience.

Response:

We sincerely appreciate the reviewer’s insightful suggestion to incorporate practical implications in the abstract. In response, we carefully considered how best to highlight the practical relevance of our study for a wider audience. While we initially explored various ways to integrate practical implications throughout the abstract, we found that due to the technical nature of the research and its focus on the theoretical mechanisms of digital intelligence and financing barriers, a concise statement would be the most effective way to maintain clarity and focus. Therefore, we have added the following statement to the revised abstract:

“These findings provide valuable insights for policymakers and enterprise decision-makers on leveraging digital intelligence to overcome financing barriers and foster innovation in small and SMEs”.

We believe this addition helps bridge the gap between theoretical findings and practical applications while maintaining the abstract’s primary focus. We hope this approach addresses the reviewer’s concerns while preserving the integrity of our research.

Additionally, in light of the evolving understanding of the concept of Specialized, Refined, Differential, and Innovative (SRDI) enterprises among Chinese scholars, we have updated the terminology to align with the current academic discourse. Furthermore, we have refined the abstract’s wording to enhance its clarity and coherence. The specific changes are highlighted in red font on the page second of the “Revised Manuscript with Track Changes” document.

We hope this revision effectively addresses the reviewer’s concern and enhances the broader applicability of our study.

2.Reviewer #1 Comment on Introduction:

The introduction effectively outlines the problem and significance of the study. To enhance clarity, a more detailed explanation of the novelty of the "Specialized, Fined, Peculiar, and Innovative" framework would be beneficial.

Response:

We would like to express our sincere gratitude to Reviewer for the valuable feedback and constructive suggestions. In response to the reviewer’s comment on enhancing the explanation of the novelty of the SRDI enterprises framework in the introduction, we have expanded the discussion on the specific attributes of SRDI enterprises based on relevant literature and official documents.We have provided a more detailed explanation of the four key components of SRDI enterprises as follows:

Specialized: Focusing on core business areas to enhance capabilities in specialized production, services, and collaboration. These enterprises provide components, parts, supporting products, and services to large enterprises, major projects, and industrial chains.

Refined: Emphasizing refined production, management, and services. These enterprises gain an advantage in niche markets by offering high-quality products and services that are highly regarded for their cost-effectiveness and superior quality.

Differential: Leveraging unique resources to promote traditional craftsmanship and regional culture. By adopting distinctive techniques, technologies, formulas, or raw materials, they produce products with local or enterprise-specific characteristics.

Innovative: Engaging in technological, management, and business model innovations to cultivate new growth areas and create new competitive advantages.

We would like to clarify that our initial intention was to provide a comprehensive explanation of SRDI enterprises for both you and the readers. However, we also realized that offering a detailed description of SRDI enterprises resulted in an excessive manuscript length. Therefore, to enhance the focus and coherence of our manuscript, we have streamlined the discussion by retaining only the key characteristics of SRDI enterprises that are directly relevant to our research. This revision not only improves readability but also ensures that our theoretical framework remains concise and well-aligned with the study’s objectives. The specific changes are highlighted in red font on the page three of the “Revised Manuscript with Track Changes” document.

This enhanced explanation aims to clarify the unique characteristics of SRDI enterprises and provide readers with a deeper understanding of their essential features. We hope these revisions help strengthen the manuscript's clarity and readability.

3.Reviewer #1 Comment on Literature Review:

The review provides a comprehensive summary of related studies but could include a deeper critique of existing gaps to highlight the study’s contribution.

Response:

We sincerely appreciate the reviewer’s insightful suggestion to enhance the critique of existing literature. In response to this, we have expanded the discussion on the gaps in the current literature, particularly in the context of China’s innovation landscape. Specifically, we highlight the persistent issues at both the micro and macro levels, such as strategic innovation driven by quantity rather than quality, and the prevalent phenomenon of “quantity-driven, quality-deficient” innovation. We also note that existing studies on patents tend to emphasize quantity over quality, which may introduce biases in the interpretation of research findings.

To provide further context, it is important to note that, since the post-economic-crisis era, China has created what is often referred to as the “China Miracle” in innovation, primarily driven by patent growth. According to the 2023 World Intellectual Property Indicators report by the World Intellectual Property Organization (WIPO), China has been the world leader in patent filings for five consecutive years, with 1.64 million invention patents filed in 2023 alone. Among these, corporate patent filings accounted for 1.13 million, ranking China 11th globally in terms of enterprise innovation. However, this rapid patent growth has also exposed significant issues, including low rates of commercialization and industrialization of research outcomes, as well as an increasing share of non-invention patents. These factors indicate that, although the quantity of innovation output has grown explosively, the quality of innovation may not have kept pace.

While we did not include these specific details in the manuscript due to space limitations, they were instrumental in shaping our critique of existing research and informing the revisions made to our paper. We hope that these additions further emphasize the contribution of our study by addressing these critical gaps in the literature. The specific changes are highlighted in red font on the page ten of the “Revised Manuscript with Track Changes” document.

We hope these revisions effectively highlight the contribution of our study by addressing these critical gaps in the literature.

4.Reviewer #1 Comment on Methodology:

The methodology is robust, but additional justification for selecting certain control variables (e.g., asset turnover ratio) would strengthen this section.

Response:

We thank the reviewer for the valuable suggestion to provide additional justification for the selection of certain control variables. In response to this, we have strengthened the explanation for the inclusion of these variables in our analysis. Specifically, we now provide a more detailed rationale for each control variable used in the study:

Ownership Concentration (Top1): measured by the largest shareholder’s share ratio, reflects the influence of ownership structure on innovation performance. Greater concentration can centralize decision-making, shaping strategic direction, innovation priorities, and resource allocation.

Board Size (Board): indicated by the number of sitting directors, is considered as it may reflect the firm’s governance quality. A larger board may provide diverse perspectives, potentially fostering a more innovation-driven environment. However, excessively large boards could introduce coordination challenges that might hinder timely and effective decision-making.

Market Competition (HHI): measured by the Herfindahl-Hirschman Index of the industry, controls for competitive pressures on innovation. Higher market concentration (higher HHI) may reduce competition, weakening innovation incentives, while lower concentration intensifies competition, fostering innovation.

Asset Turnover Ratio (ATO): calculated as the ratio of net operating revenue to average total assets, reflects how effectively a firm utilizes its assets to generate revenue. A higher ATO suggests better asset use, enabling greater resource allocation for innovation. Firms with higher turnover may also be more agile in deploying resources for technological advancements.

Operating Cycle (Cycle): which refers to the time taken from acquiring inventory to selling it and recovering cash, is included to control for the firm’s operational efficiency. Firms with shorter cycles manage working capital better, enabling more financial resources for innovation.

Operating Cash Flow (OCF): represented by the net cash flow generated from operating activities, controls for the financial health of the firm. Strong OCF reflects the firm’s ability to generate internal funds, reducing reliance on external financing and supporting stable innovation investments.

Return on Assets (ROA): measured by the ratio of earnings before interest and taxes to average total assets, is included to account for the firm’s overall financial performance and efficiency. A higher ROA indicates better profitability and asset management, enhancing the firm’s ability to fund and sustain innovation.

After careful revision, we realized that providing detailed explanations of the control variables within the main text might make the paper overly lengthy and distract from the primary discussion. To maintain clarity and conciseness, we have streamlined this section while ensuring that all necessary details remain accessible to readers. Although we have omitted the detailed descriptions from the main text, the rationale and selection criteria for the control variables are still rigorously justified in the methodology section. We believe this approach enhances the manuscript’s readability while maintaining its academic rigor. The specific changes are highlighted in red font on the page nineteen the “Revised Manuscript with Track Changes” document.

By providing clear justifications for each of the control variables, we hope to strengthen the robustness of the variable selection and enhance the overall credibility of our methodology.

5.Reviewer #1 Comment on Results and Discussion:

The results are well-presented and supported by appropriate statistical analysis. However, the discussion could better contextualize the findings in relation to broader global trends in digital intelligence adoption.

Response:

We thank the reviewer for the helpful suggestion to better contextualize our findings within broader global trends in digital intelligence adoption. We acknowledge that the original manuscript primarily presented basic regression results without delving into the implications and connections to real-world trends. To address this, we have expanded the discussion section to include a deeper exploration of the global digital transformation trend, particularly focusing on the adoption of digital intelligence technologies and their role in driving innovation. Specifically, we have added the following content:

The ongoing global digital transformation trend increasingly recognizes the application of digital intelligence technologies as a key driver of innovation. In particular, SMEs in leading economies such as the United States, Germany, and Japan have been rapidly adopting digital technologies to enhance their innovation capabilities. National initiatives such as Germany’s “Industry 4.0” and Japan’s “Society 5.0” underscore the integration of smart technologies across both manufacturing and service sectors. Similarly, in China, the adoption of digital intelligence—encompassing artificial intelligence and data-driven technologies—has played a pivotal role in accelerating innovation within specialized enterprises. A prime example is the recently launched R1 model by DeepSeek, which has surpassed OpenAI in several tests and challenged Nvidia’s “computing power myth”. The R1 model demonstrates that, through algorithmic optimization, 60% of global AI computing power can be supported by mid-range chips, thus eliminating the need for costly high-performance chips. This breakthrough highlights AI’s transformative potential in reducing dependence on expensive infrastructure, while also underscoring China’s growing influence in the

Attachment Submitted filename: Response to Reviewers.docx

# Peer review: Decision Letter 1

**DOI:** 10.1371/journal.pone.0339567.r003

**Authors:** Yasir Ahmad

15 Jul 2025

Dear Dr. Zhang,

Thank you for submitting your manuscript to PLOS ONE. After careful consideration, we feel that it has merit but does not fully meet PLOS ONE’s publication criteria as it currently stands. Therefore, we invite you to submit a revised version of the manuscript that addresses the points raised during the review process. Elaborate comments are provided by one of the reviewers to improve the work.

Please submit your revised manuscript by Aug 29 2025 11:59PM. If you will need more time than this to complete your revisions, please reply to this message or contact the journal office at plosone@plos.org . When you're ready to submit your revision, log on to https://www.editorialmanager.com/pone/ and select the 'Submissions Needing Revision' folder to locate your manuscript file.

- A rebuttal letter that responds to each point raised by the academic editor and reviewer(s). You should upload this letter as a separate file labeled 'Response to Reviewers'.
- A marked-up copy of your manuscript that highlights changes made to the original version. You should upload this as a separate file labeled 'Revised Manuscript with Track Changes'.
- An unmarked version of your revised paper without tracked changes. You should upload this as a separate file labeled 'Manuscript'.

If applicable, we recommend that you deposit your laboratory protocols in protocols.io to enhance the reproducibility of your results. Protocols.io assigns your protocol its own identifier (DOI) so that it can be cited independently in the future. For instructions see: https://journals.plos.org/plosone/s/submission-guidelines#loc-laboratory-protocols . Additionally, PLOS ONE offers an option for publishing peer-reviewed Lab Protocol articles, which describe protocols hosted on protocols.io. Read more information on sharing protocols at https://plos.org/protocols?utm_medium=editorial-email&utm_source=authorletters&utm_campaign=protocols .

We look forward to receiving your revised manuscript.

Kind regards,

Yasir Ahmad

Academic Editor

PLOS ONE

Journal Requirements:

If the reviewer comments include a recommendation to cite specific previously published works, please review and evaluate these publications to determine whether they are relevant and should be cited. There is no requirement to cite these works unless the editor has indicated otherwise.

Please review your reference list to ensure that it is complete and correct. If you have cited papers that have been retracted, please include the rationale for doing so in the manuscript text, or remove these references and replace them with relevant current references. Any changes to the reference list should be mentioned in the rebuttal letter that accompanies your revised manuscript. If you need to cite a retracted article, indicate the article’s retracted status in the References list and also include a citation and full reference for the retraction notice.

[Note: HTML markup is below. Please do not edit.]

Reviewers' comments:

Reviewer's Responses to Questions

**Comments to the Author**

Reviewer #2: (No Response)

Reviewer #3: All comments have been addressed

**********

2. Is the manuscript technically sound, and do the data support the conclusions?

Reviewer #2: Yes

Reviewer #3: Yes

**********

3. Has the statistical analysis been performed appropriately and rigorously?

Reviewer #2: Yes

Reviewer #3: Yes

**********

4. Have the authors made all data underlying the findings in their manuscript fully available?

The PLOS Data policy

Reviewer #2: Yes

Reviewer #3: Yes

**********

5. Is the manuscript presented in an intelligible fashion and written in standard English?

Reviewer #2: Yes

Reviewer #3: No

**********

Reviewer #2: (No Response)

Reviewer #3: Abstract: Fix minor typos (e.g., duplicate phrase: “intelligent technologies intelligent technology”). Add one line on practical implications to connect with broader readers.

Introduction: Briefly mention how your findings might be useful for SMEs outside China, especially in other emerging economies.

Methodology:

Include a simple explanation or visual example of how you measured digital intelligence using keyword frequency in reports.

Clarify how the “knowledge breadth” metric was constructed from patent data for readers unfamiliar with IPC codes.

Results & Analysis:

Add a few practical examples or short takeaways that SMEs could apply based on your findings.

When describing effects (e.g., digital intelligence improving innovation), consider explaining how big the effect is (if possible).

Discussion:

Try to reduce repetition in sections discussing China’s innovation ecosystem and tech trends. Streamline for clarity.

Emphasize the unique value of intelligent technologies compared to datafication and digitization more concisely.

Language & Style:

The paper is generally clear, but a final language edit is needed to fix awkward phrasing, long sentences, and grammar issues.

Conclusion:

Briefly outline future research directions (e.g., international comparisons, long-term digital effects).

Add one sentence showing how SMEs can use digital tools for long-term growth, based on your findings.

**********

what does this mean? ). If published, this will include your full peer review and any attached files.

If you choose “no”, your identity will remain anonymous but your review may still be made public.

**Do you want your identity to be public for this peer review?** For information about this choice, including consent withdrawal, please see our Privacy Policy

Reviewer #2: No

Reviewer #3: No

**********

[NOTE: If reviewer comments were submitted as an attachment file, they will be attached to this email and accessible via the submission site. Please log into your account, locate the manuscript record, and check for the action link "View Attachments". If this link does not appear, there are no attachment files.]

While revising your submission, please upload your figure files to the Preflight Analysis and Conversion Engine (PACE) digital diagnostic tool, https://pacev2.apexcovantage.com/ . PACE helps ensure that figures meet PLOS requirements. To use PACE, you must first register as a user. Registration is free. Then, login and navigate to the UPLOAD tab, where you will find detailed instructions on how to use the tool. If you encounter any issues or have any questions when using PACE, please email PLOS at figures@plos.org

# Author comment: Author response to Decision Letter 2

**DOI:** 10.1371/journal.pone.0339567.r004

24 Aug 2025

Dear Editor Ahmad,

We sincerely appreciate your review of our manuscript titled “The Impact of Digital Intelligence on Innovation Performance: Evidence from Specialized, Refined, Differential, and Innovative (SRDI) enterprises” (PONE-D-24-53233R1) and your valuable feedback. We are grateful for the thoughtful comments provided by the reviewer, and we have made comprehensive revisions in response to all the feedback.

Below are our detailed responses to each comment.

Dear Reviewer #2 and Reviewer #3

We would like to begin by sincerely thanking you for your valuable and insightful comments on our manuscript. Your feedback has been instrumental in improving the quality of our paper, and we have made several revisions and additions in response to your suggestions. We believe these changes have enhanced the scientific rigor and applicability of our study. Below, we outline the specific improvements made based on your comments.

1.Reviewer #3

The manuscript is not written in standard English. The language is not clear and intelligible.

Response:

We sincerely thank the reviewer for this valuable comment. In order to address the language concern, we have engaged a professional English-language editing service. The entire manuscript has been carefully polished to ensure that the writing is clear, correct, and unambiguous. We have also uploaded the official editing certificate as a supplementary file (Certificate), which confirms that the manuscript has undergone professional language editing. We hope this resolves the reviewer’s concern.

2.Reviewer #3 Comment on Abstract:

Fix minor typos (e.g., duplicate phrase: “intelligent technologies intelligent technology”). Add one line on practical implications to connect with broader readers.

Response:

We sincerely appreciate the reviewer’s careful reading of our manuscript and the constructive feedback provided. Thank you for pointing out the minor typographical errors in the Abstract and for suggesting that we add a line on the practical implications of our research to make our findings more accessible to a broader readership. We value these suggestions and have addressed them as follows:

We have carefully reviewed and corrected any typographical errors in the Abstract, including removing duplicate phrases. In addition, we have added a sentence summarizing the practical implications of our findings to broaden the relevance and impact of our research.

The revised Abstract now reads as follows:

“These findings provides valuable guidance for policymakers seeking to develop targeted and differentiated strategies to enhance the innovation capacity of SRDI enterprises. In particular, the results underscore the role of digital intelligence in easing financing constraints. By accounting for firm-level heterogeneity, this study offers a robust theoretical and empirical foundation for policy design and delivers actionable recommendations for enterprises aiming to optimize resource allocation and strengthen innovation capabilities through digital transformation.” The specific changes are highlighted in red font on the page 2 of the “Revised Manuscript with Track Changes” document.

We sincerely appreciate the reviewer’s careful and constructive feedback. We hope that the changes we have made are satisfactory. If there are any further questions or suggestions, we would be grateful to receive them and will revise the manuscript accordingly.

3.Reviewer #3 Comment on Introduction:

Briefly mention how your findings might be useful for SMEs outside China, especially in other emerging economies.

Response:

Thank you for this valuable suggestion. In the revised manuscript, we clarify that our findings are particularly relevant for SMEs in other emerging economies that share similar institutional environments, financial constraints, and industrial structures with China. Our results demonstrate that digital intelligence significantly enhances innovation performance and alleviates financing constraints for SMEs—a mechanism likely to be applicable in countries such as India, Vietnam, Indonesia, and Brazil, where digital transformation in the SME sector is well underway.

Furthermore, our study provides actionable guidance for policymakers and business leaders in economies where the adoption of digital intelligence technologies is still at an early or exploratory stage. For example, in Egypt and Turkey, many SMEs remain cautious about investing in cloud-based services due to concerns over cybersecurity, cost, and a lack of specialized IT staff. In the Philippines and South Africa, small businesses often operate in fragmented markets with limited access to formal finance, making them hesitant to transition from manual to digital operations. Nevertheless, pilot projects—such as Egyptian SMEs collaborating with fintech startups to implement mobile-based accounting systems and Turkish business associations rolling out cloud adoption programs—demonstrate that the practical benefits of digital intelligence are already emerging.

Importantly, the widespread availability of cloud-based platforms enables SMEs in emerging markets to access advanced technological tools without significant up-front investment or specialized technical teams. These solutions lower the barriers to digital adoption and shorten the learning curve, making digital transformation possible even for firms with limited resources.

Although SMEs across countries face different institutional and regulatory environments, the underlying logic of digital intelligence empowerment is consistent: by reducing the costs and complexity of technology acquisition, digital platforms unlock innovation potential and promote sustainable growth in diverse contexts.

Due to space limitations, this discussion has been summarized in a concise statement in the revised Introduction. The revised Abstract now reads as follows:

“Comparable trends are evident in countries like India, Brazil, and Vietnam, where cloud-based platforms and digital financial tools are increasingly used to support SME innovation. In contrast, countries such as Egypt and Turkey have adopted a more cautious approach, with many firms still assessing the opportunities and risks of digital transformation. Although institutional and regulatory conditions vary across countries, the fundamental mechanism by which digital intelligence empowers firms remains consistent. By reducing the costs and barriers associated with technology adoption, digital intelligence enables enterprises to unlock their innovation potential across a range of economic and institutional settings.” The specific changes are highlighted in red font on page 5 to 6 of the “Revised Manuscript with Track Changes” document.

We would be happy to provide additional examples or further details if required. Once again, we appreciate the reviewer’s constructive feedback and remain open to further suggestions.

4.Reviewer #3 Comment on Methodology:

Include a simple explanation or visual example of how you measured digital intelligence using keyword frequency in reports.

Clarify how the “knowledge breadth” metric was constructed from patent data for readers unfamiliar with IPC codes.

Response:

Thank you for your valuable comments. We have addressed each suggestion separately as follows:

Measurement of digital intelligence using keyword frequency:

In response to the reviewer’s suggestion, we have supplemented the Methods section with a concise explanation and a visual example of the keyword frequency method. Specifically, digital intelligence was measured by counting the occurrences of five core digital technology keywords—cloud computing, big data, blockchain, artificial intelligence, and digital technology applications—in the annual reports of all sampled firms. For example, if the report of Firm A contained “cloud computing” 10 times, “big data” 6 times, and “artificial intelligence” 4 times, these values were recorded as the raw digital intelligence scores for that firm, as shown in columns 3 to 7 of Table 1.

The construction of the digital intelligence index involved the following main steps:

(1) standardization of the raw keyword frequencies; (2) correlation testing using the Kaiser-Meyer-Olkin (KMO) and Bartlett’s tests to confirm suitability for factor analysis (with a KMO value greater than 0.723); (3) factor analysis and calculation of eigenvalues and eigenvectors; (4) principal component selection based on eigenvalues and unit eigenvectors, as detailed in the main text; (5) construction of the composite index using the weighted principal components. The principal component loadings (contribution rates), denoted as c1, c2, c3, and c4, were then used to calculate the composite digital intelligence index as follows:

Ctotal=�c1×variance contribution of PC1+c2×variance contribution of PC2+c3×variance contribution of PC3+c4×variance contribution of PC4�/cumulative variance contribution of PC4

For clarity, we have also included a summary workflow diagram as follows:

Annual Report Text → Keyword Extraction and Counting → Standardization → Factor Analysis → Index Construction.

We hope that this explanation provides a clear understanding of our procedures. Should you have any further questions or require additional clarification, please let us know. We would be very happy to provide further details.

Table 1 Digital Intelligence Index Data

code year Cloud Big data Blockchain AI digital technology c1 c2 c3 c4 DiginteIndex lnDiginteIndex

Firm A 2014 0 0 0 0 16 -0.07 -0.62 0.47 0.51 0.01 0.01

Firm A 2015 0 0 1 0 5 -0.29 -0.12 0.10 0.20 -0.11 -0.12

Firm A 2016 0 0 0 0 16 -0.07 -0.62 0.47 0.51 0.01 0.01

Firm A 2017 1 0 0 0 6 -0.27 -0.18 0.18 0.14 -0.11 -0.12

Firm A 2018 0 0 0 1 1 -0.27 0.40 0.27 0.17 0.02 0.02

Firm A 2019 0 0 0 0 2 -0.41 0.01 0.04 0.09 -0.17 -0.19

Firm A 2020 0 0 0 0 3 -0.39 -0.03 0.07 0.12 -0.16 -0.17

Firm A 2021 0 0 0 0 2 -0.41 0.01 0.04 0.09 -0.17 -0.19

Firm A 2022 0 1 0 0 2 -0.35 0.01 -0.03 0.11 -0.15 -0.17

Firm A 2023 0 2 0 0 2 -0.30 0.02 -0.09 0.12 -0.14 -0.15

Table 2 Results of the multi-grid principal component analysis

Principal Component Initial Eigenvalue

Eigenvalue Variance Contribution (%) Cumulative Variance Contribution (%)

1 2.171 43.42 43.42

2 0.891 17.81 61.23

3 0.789 15.78 77.01

4 0.685 13.7 90.71

5 0.464 9.29 100

Construction of the “knowledge breadth” metric from patent data:

Thank you for your helpful suggestion. In the revised Methods section, we have provided a clear and detailed explanation of how the “knowledge breadth” metric was constructed from patent data, especially for readers unfamiliar with IPC codes.

Specifically, knowledge breadth reflects the diversity of technological fields represented in a firm’s patents. Each patent can be assigned one or more International Patent Classification (IPC) codes, which can be grouped into broader “main groups.” To calculate the knowledge breadth for each patent, we first determined the main groups covered by its IPC codes and the proportion of codes in each main group.

For example, as shown in Table 3, Patent 1 of Firm A has two IPC classification codes: H05B37/02 and H05B37/02. Both belong to the same main group “B37,” so the proportion is 2/2 = 1. According to the formula by Zhang and Zheng, the knowledge breadth for this patent is calculated as:

patent_knowledge = 1 – (2/2)² = 0.

Similarly, Patent 2 of Firm A has three IPC codes—G06F3/045, G06F3/044, and G06F3/045—all belonging to the main group “F3,” giving a proportion of 3/3 = 1 and a knowledge breadth of:

patent_knowledge = 1 – (3/3)² = 0.

Patent 3 of Firm A includes three IPC codes: H02H7/26, H02H1/00, and H02H7/26, covering two main groups (“H7” and “H1”) with proportions of 2/3 and 1/3, respectively. The knowledge breadth is calculated as:

patent_knowledge = 1 – [(2/3)² + (1/3)²] = 0.4444.

This approach follows the method of Zhang and Zheng (full citation in the main text) and can also be calculated directly in Stata using the code provided below:

* Import patent data

* Split IPC codes into separate variables

split ipc_codes, parse(" ") gen(ipc_code_)

* 1. Extract the main group part (before "/") for each IPC classification code

gen main_group_1 = substr(ipc_code_1, 1, strpos(ipc_code_1, "/") - 1)

gen main_group_n = substr(ipc_code_n, 1, strpos(ipc_code_n, "/") - 1)

* Generate HHI value for knowledge breadth

* Create a new variable `result` to store the calculation result for each row

gen result = 0

* Iterate over each row of data

forval row = 1/`=_N' {

* Retrieve the quantity for the current row

local quantity = quantity[`row']

* Initialize result to 0

local row_result = 0

* Create a local macro to store unique values for the current row

local unique_values ""

* Iterate through each column (main_group_1 to main_group_n)

foreach col of varlist main_group_1-main_group_n {

* Retrieve the value in the current column

local value = `col'[`row']

* Determine whether the current value already exists in unique_values

if "`value'" != "" & !strpos("`unique_values'", "`value'") {

* If the value is not in unique_values, proceed with counting

* Count the occurrences of the current value in this row

local count = 0

foreach col2 of varlist main_group_1-main_group_n {

if `col2'[`row'] == "`value'" {

local count = `count' + 1

}

}

* Calculate the square of the frequency of this value and add it to row_result

local result_part = (`count' / `quantity')^2

local row_result = `row_result' + `result_part'

* Update unique_values to avoid double-counting

local unique_values "`unique_values' `value'"

}

}

* Store the calculation result for each row in the result variable

replace result = `row_result' in `row'

}

We hope that this clarification improves the accessibility and transparency of our methodology. If you have any further questions or require additional information, please let us know. We would be pleased to provide further details.

Table 3 Example of Knowledge Breadth Calculation

code IPC Code IPC Code1 IPC Code2 IPC Code3 patent_knowledge

Firm A H05B37/02 H05B37/02 H05B37/02 H05B37/02 0

Firm A G06F3/045;G06F3/044 G06F3/045 G06F3/045 G06F3/044 G06F3/045 0

Firm A H02H7/26;H02H1/00 H02H7/26 H02H7/26 H02H1/00 H02H7/26 0.4444

5.Reviewer #3 Comment on Results & Analysis:

Add a few practical examples or short takeaways that SMEs could apply based on your findings.

When describing effects (e.g., digital intelligence improving innovation), consider explaining how big the effect is (if possible).

Response:

Add a few practical examples or short takeaways that SMEs could apply based on your findings.

We thank the reviewer sincerely for this valuable suggestion. We agree that providing practical examples and actionable takeaways would greatly enhance the practical relevance of our study. In response, we have added specific summary statements after each main empirical finding in the revised manuscript to guide SMEs on how to apply our research in practice. The main additions are as follows:

Baseline Regression results:

“In summary, the results highlight that in resource-constrained environments, SRDI enterprises should strategically prioritize the adoption of digital intelligence tools, especially cloud computing, artificial intelligence, and digital technology applications, as these exert the strongest positive effects on both innovation efficiency and innovation quality.” The specific changes are highlighted in red font on page 25 of the “Revised Manuscript with Track Changes” document.

Mediating Effect Analysis results:

“Such experiences suggest that the adoption of digital finance and analytics platforms could also help SMEs in other countries address similar funding barriers, thereby fostering sustainable innovation.” The specific changes are highlighted in red font on page 28 of the “Revised Manuscript with Track Changes” document.

Regional heterogeneity (East-West) results:

“This suggests that, while regional disparities exist

Attachment Submitted filename: Response_to_Reviewers_auresp_2.docx

# Peer review: Decision Letter 2

**DOI:** 10.1371/journal.pone.0339567.r005

**Authors:** Annesha Sil

25 Sep 2025

PONE-D-24-53233R2

The Impact of Digital Intelligence Technologies on Innovation Performance: Evidence from Specialized, Refined, Differential and Innovative enterprises

PLOS ONE

Dear Dr. Zhang,

Thank you for submitting your manuscript to PLOS ONE. After careful consideration, we have decided that your manuscript does not meet our criteria for publication and must therefore be rejected.

PLOS One has been specifically designed for the publication of the results of original research contributing to the base of academic knowledge. Further, for manuscripts to be considered for publication in PLOS One, experiments and analyses must be conducted rigorously, with appropriate controls; methods must be described in sufficient detail for others to replicate the analyses; and conclusions must be supported by the results presented (http://journals.plos.org/plosone/s/criteria-for-publication). After careful consideration, we feel that the current manuscript does not meet these requirements.

Specifically, we noted that despite two rounds of revision, the exact sources of data for each variable have not been clarified. Further the keywords used for the construction of the digitization index has not been provided. Further, we have major concerns about the contribution of this study as there appear to be several other published works investigating very similar research questions using similar methodologies have not been adequately cited or discussed.

I am sorry that we cannot be more positive on this occasion, but hope that you appreciate the reasons for this decision.

Kind regards,

Annesha Sil, Ph.D.

Staff Editor

PLOS One

Additional Editor Comments (if provided):

Reviewer #3:

[Note: HTML markup is below. Please do not edit.]

Reviewers' comments:

Reviewer's Responses to Questions

**Comments to the Author**

Reviewer #3: All comments have been addressed

**********

2. Is the manuscript technically sound, and do the data support the conclusions?

Reviewer #3: Yes

**********

3. Has the statistical analysis been performed appropriately and rigorously?

Reviewer #3: Yes

**********

4. Have the authors made all data underlying the findings in their manuscript fully available?

The PLOS Data policy

Reviewer #3: Yes

**********

5. Is the manuscript presented in an intelligible fashion and written in standard English?

Reviewer #3: Yes

**********

Reviewer #3: I would like to commend the authors for their careful and thorough revisions. The methodology is now clearly explained with illustrative examples, the inclusion of effect sizes strengthens the results, and the discussion has been streamlined to highlight the distinct value of intelligent technologies. The professional language editing has greatly improved readability, though a few sentences in the introduction and discussion could still be shortened for clarity. Overall, the paper is now clear, rigorous, and well-prepared for publication.

**********

what does this mean? ). If published, this will include your full peer review and any attached files.

If you choose “no”, your identity will remain anonymous but your review may still be made public.

**Do you want your identity to be public for this peer review?** For information about this choice, including consent withdrawal, please see our Privacy Policy

Reviewer #3: No

**********

[NOTE: If reviewer comments were submitted as an attachment file, they will be attached to this email and accessible via the submission site. Please log into your account, locate the manuscript record, and check for the action link "View Attachments". If this link does not appear, there are no attachment files.]

- - - - -

For journal use only: PONEDEC3

# Author comment: Author response to Decision Letter 3

**DOI:** 10.1371/journal.pone.0339567.r006

24 Nov 2025

Dear Dr. Annesha Sil and Reviewer #3,

We sincerely appreciate your time and effort in handling our manuscript entitled “The Impact of Digital Intelligence Technologies on Innovation Performance: Evidence from Specialized, Refined, Differential, and Innovative Enterprises” (Manuscript ID: PONE-D-24-53233R2). We are grateful for your thoughtful evaluation and for the detailed feedback provided in your letter. We have carefully reviewed each point raised and have revised the manuscript accordingly to ensure full methodological transparency and to clarify the study’s theoretical contribution.

Below, we provide detailed responses to every comment and explain the corresponding revisions made in the revised version and supplementary materials.

1.Dr. Annesha Sil Comment on data sources:

Despite two rounds of revision, the exact sources of data for each variable have not been clarified.

The keywords used for the construction of the digitization index have not been provided.

Response:

We sincerely appreciate this valuable comment. In the original manuscript, the data sources for all variables were presented in Section 4 (Method and Data, pp. 14–15). However, we recognize that our explanation may not have been sufficiently detailed for readers without a background in economics or management science. To improve clarity and ensure full transparency for an interdisciplinary readership, we have now expanded and refined our descriptions of each data source and variable mapping in both the main text and supplementary materials. Specifically:

(1)Financial variables are now explicitly stated as being obtained from CSMAR and the iFinD Financial Data Terminal, while the SRDI firm lists (batches 1–6) are collected from official MIIT announcements. Patent data used to construct innovation-quality indicators are sourced from the CNIPA Patent Search and Analysis Database, with IPC information consistent with the WIPO IPC scheme. The three-stage DEA model uses input, output and environmental variables derived from these sources. To ensure full transparency, we have added a complete variable–source mapping table in Appendix A4 in S1 Appendix.

(2)The complete list of SRDI enterprises used in this study is now provided in S2 Appendix, corresponding to MIIT batches 1–6.

(3)The computation steps for IPC-based knowledge breadth—including formulas, worked examples, and Stata code—are reported in Appendix A3 in S1 Appendix.

(4)We have added the complete construction workflow and diagnostic procedures for the Digital Intelligence Index in Appendix A2 in S1 Appendix.

(5)the complete keyword dictionary used for identifying cloud computing, big data, artificial intelligence, blockchain, and digital applications is now included in Appendix A1 in S1 Appendix.

(6)The full three-stage DEA computation—implemented using DEAP 2.1 (DEA stages) and FRONTIER 4.1 (stochastic-frontier adjustment)—is documented in Appendix A5 in S1 Appendix.

All of the above data descriptions and supporting materials are fully documented in S1 Appendix. These additions substantially improve the transparency, reproducibility, and completeness of the manuscript. We are grateful for the reviewer’s suggestion, which has helped strengthen the clarity and methodological rigor of the study.

Table 1. Keyword Dictionary for Digital Intelligence Index

Artificial Intelligence Technology

Artificial Intelligence

Machine Learning

Deep Learning

Natural Language Processing

Image Understanding

Semantic Search

Voice Recognition

Facial Recognition

Biometric Identification Technology

Intelligent Data Analysis

Brain-like Computing

Cognitive Computing

Intelligent Robots

Autonomous Driving

Big Data Technology

Big Data

Data Mining

Text Mining

Data Visualization

Stream Computing

Graph Computing

In-Memory Computing

Heterogeneous Data

Distributed Computing

Billion-level Concurrency

EB-level Storage

Investment Decision Support System

Business Intelligence

Cloud Computing Technology

Cloud Computing

Green Computing

Converged Architecture

Blockchain Technology

Blockchain

Differential Privacy Technology

Multi-Party Secure Computation

Smart Financial Contracts

Digital Currency

Digital Technology Application

Mobile Internet

Internet of Things (IoT)

Cyber-Physical Systems

Industrial Internet

Mobile Payment

Third-Party Payment

NFC Payment

Internet Finance

Digital Finance

Fintech

Financial Technology

Quantitative Finance

Open Banking

E-Commerce

B2B (Business-to-Business)

B2C (Business-to-Consumer)

C2B (Consumer-to-Business)

C2C (Consumer-to-Consumer)

O2O (Online-to-Offline)

Internet Healthcare

Smart Home

Smart Wearables

Intelligent Customer Service

Intelligent Marketing

Digital Marketing

Unmanned Retail

Smart Agriculture

Intelligent Transportation

Intelligent Healthcare

Intelligent Cultural Tourism

Intelligent Environmental Protection

Smart Grid

Intelligent Investment Advisory

Intelligent Energy

Identity Verification

Credit Investigation

2.Dr. Annesha Sil Comment on contribution:

Major concerns remain about the contribution of this study, as there appear to be several other published works investigating very similar research questions using similar methodologies that have not been adequately cited or discussed.

Response:

We sincerely thank the editor for this valuable and constructive comment. We fully understand the importance of situating our contribution within the broader research context. In the earlier version, while we had cited general studies on digitalization and innovation, we acknowledge that we did not explicitly contrast our framework with the most recent and thematically similar empirical works. This omission may have made our theoretical and methodological contribution appear less distinctive than it actually is.

To address this issue comprehensively, we have revised the “Main Contributions” section of the manuscript to clarify how this study extends existing research:

(1)We explicitly highlight that we disaggregate digital intelligence into five distinct technologies (cloud computing, artificial intelligence, blockchain, big data, and digital applications), whereas prior studies commonly rely on a single aggregated digitalisation measure. More importantly, this disaggregation reveals substantial heterogeneity in their effects: while all five technologies enhance innovation efficiency, only cloud computing, artificial intelligence, and digital applications significantly improve innovation quality, and blockchain can even exert a suppressive effect on innovation quality in certain contexts (e.g., state-owned and non-manufacturing firms). This finding underscores that not all digital intelligence technologies are uniformly beneficial, providing a more nuanced understanding than aggregate-level analyses. These results may also offer useful insights for resource-constrained SMEs in other emerging economies, suggesting that firms should adopt digital intelligence technologies selectively according to their own developmental stage and goals. For instance, all five technologies can support innovation efficiency, but prioritizing cloud computing, artificial intelligence, and digital applications appears particularly effective for enhancing innovation quality, whereas blockchain requires cautious application given its limited or context-dependent benefits.

(2) We emphasise our focus on SRDI enterprises, a policy-designated group that has rarely been examined in the digital-transformation literature despite their central role in China’s innovation strategy.

(3) We incorporate financing constraints and institutional characteristics into the analysis, thereby providing new evidence on the mechanisms linking digital intelligence to innovation performance.

(4) We have added targeted discussions and citations to published studies that use comparable empirical approaches or analyse digitalisation and innovation in emerging economies, including research on cloud-based management systems and digital finance in India, digital strategies in Brazilian family-owned firms, and the interaction between digitalisation and knowledge management in Vietnam. These additions clarify how our work builds upon, yet remains distinct from, existing contributions.

These revisions strengthen the clarity, positioning and novelty of the study, and we sincerely appreciate the reviewer’s guidance in improving this aspect. The specific changes are highlighted in red on pages 5–6 of the “Revised Manuscript with Tracked Changes” document.

3.Dr. Reviewer #3Comment :

I would like to commend the authors for their careful and thorough revisions. The methodology is now clearly explained with illustrative examples, the inclusion of effect sizes strengthens the results, and the discussion has been streamlined to highlight the distinct value of intelligent technologies. The professional language editing has greatly improved readability, though a few sentences in the introduction and discussion could still be shortened for clarity. Overall, the paper is now clear, rigorous, and well-prepared for publication.

Response:

We sincerely thank Reviewer #3 for the positive and encouraging evaluation. We greatly appreciate your recognition of the improvements made to the manuscript and your helpful suggestion to further enhance clarity.

In response, we have shortened and refined several long sentences in the Introduction section (pp. 2–3) to improve readability and conciseness while maintaining the intended meaning. Specifically, we revised the following sentences:

Original:

“Over the past four decades, China’s economy has experienced rapid transformation, driven largely by reform and opening-up policies that introduced market-oriented mechanisms and expanded integration with the global economy.”

Revised:

“Over the past four decades, China’s economy has transformed through market reforms and global integration.”

Original:

“Differentiation leverages unique resources, traditional craftsmanship, and regional cultural elements to create distinctive products with competitive advantages.”

Revised:

“Differentiation builds on unique resources and regional culture to foster competitive advantages.”

Original:

“They form the backbone of the country’s mid-to high-end manufacturing supply chain, play a central role in high-quality development, and serve as an essential pillar in the new development paradigm, as well as a significant contributor to building an innovative nation.”

Revised:

“They underpin China’s advanced manufacturing and support innovation-driven growth.”

These modifications reduce redundancy and improve fluency while preserving the theoretical accuracy and contextual meaning. We believe this targeted refinement further enhances the clarity, conciseness, and professional readability of the manuscript.The specific changes are highlighted in red font on the page 2 to 3 of the “Revised Manuscript with Tracked Changes” document.

4.Reviewer #3 ((PONE-D-24-53233R1): Comment on Introduction:

Briefly mention how your findings might be useful for SMEs outside China, especially in other emerging economies.

Response:

Thank you for this helpful suggestion. Although we had previously added a brief discussion in response to this comment, the contribution section has been substantially rewritten in the current revision to make the positioning and novelty of the study clearer. Accordingly, we have updated the relevant text to the following:

“These findings on the heterogeneous effects of the five digital intelligence technologies may also inform technology adoption decisions for SMEs in other emerging economies. Specifically, while all five technologies can enhance innovation efficiency and cloud computing, artificial intelligence, and digital applications are particularly effective for improving innovation quality among resource-constrained firms, blockchain may exert suppressive effects on innovation quality in certain contexts. This underscores that not all digital intelligence technologies are uniformly beneficial and highlights the need for cautious, context-specific adoption.”

We hope that this revised formulation more concisely and accurately responds to Reviewer #3’s original comment while fully satisfying PLOS ONE’s publishing criteria. The changes are highlighted in the tracked-changes version of the manuscript.The specific changes are highlighted in red font on the page 6 of the “Revised Manuscript with Tracked Changes” document.

We sincerely thank the Academic Editor, the Staff Editor, and the reviewers for their time, effort, and constructive feedback throughout the review process. The comments have been invaluable in helping us improve the clarity, methodological transparency, and theoretical contribution of this work.

In the revised version, we have comprehensively addressed all the concerns by clarifying variable-level data sources(S1 Appendix), providing a full keyword dictionary for the digital intelligence index (Fig. 1), refining the introduction and discussion for conciseness, and expanding the contribution section with recent literature to strengthen theoretical grounding.

We greatly value PLOS ONE’s mission to promote cross-disciplinary dialogue and open scientific exchange, and we sincerely hope that this clarification will allow a fair reassessment of the manuscript within its disciplinary context.

Thank you once again for reconsidering our work. We look forward to your decision.

Sincerely,

The Authors

Attachment Submitted filename: Point-by-Point Response to Previous Concerns.docx

# Editor report: Decision Letter 3

**DOI:** 10.1371/journal.pone.0339567.r007

**Authors:** Zeyu Xing

9 Dec 2025

The Impact of Digital Intelligence Technologies on Innovation Performance: Evidence from Specialized, Refined, Differential and Innovative enterprises

PONE-D-24-53233R3

Dear Dr. Fa Zhang,

We’re pleased to inform you that your manuscript has been judged scientifically suitable for publication and will be formally accepted for publication once it meets all outstanding technical requirements.

Within one week, you’ll receive an e-mail detailing the required amendments. When these have been addressed, you’ll receive a formal acceptance letter and your manuscript will be scheduled for publication.

An invoice will be generated when your article is formally accepted. Please note, if your institution has a publishing partnership with PLOS and your article meets the relevant criteria, all or part of your publication costs will be covered. Please make sure your user information is up-to-date by logging into Editorial Manager at Editorial Manager®  and clicking the ‘Update My Information' link at the top of the page. For questions related to billing, please contact billing support .

If your institution or institutions have a press office, please notify them about your upcoming paper to help maximize its impact. If they’ll be preparing press materials, please inform our press team as soon as possible -- no later than 48 hours after receiving the formal acceptance. Your manuscript will remain under strict press embargo until 2 pm Eastern Time on the date of publication. For more information, please contact onepress@plos.org.

Kind regards,

Zeyu Xing

Academic Editor

PLOS ONE

Additional Editor Comments (optional):

Reviewers' comments:

# Editor report: Acceptance letter

**DOI:** 10.1371/journal.pone.0339567.r008

**Authors:** Zeyu Xing

PONE-D-24-53233R3

PLOS One

Dear Dr. Zhang,

I'm pleased to inform you that your manuscript has been deemed suitable for publication in PLOS One. Congratulations! Your manuscript is now being handed over to our production team.

At this stage, our production department will prepare your paper for publication. This includes ensuring the following:

* All references, tables, and figures are properly cited

* All relevant supporting information is included in the manuscript submission,

* There are no issues that prevent the paper from being properly typeset

You will receive further instructions from the production team, including instructions on how to review your proof when it is ready. Please keep in mind that we are working through a large volume of accepted articles, so please give us a few days to review your paper and let you know the next and final steps.

Lastly, if your institution or institutions have a press office, please let them know about your upcoming paper now to help maximize its impact. If they'll be preparing press materials, please inform our press team within the next 48 hours. Your manuscript will remain under strict press embargo until 2 pm Eastern Time on the date of publication. For more information, please contact onepress@plos.org.

You will receive an invoice from PLOS for your publication fee after your manuscript has reached the completed accept phase. If you receive an email requesting payment before acceptance or for any other service, this may be a phishing scheme. Learn how to identify phishing emails and protect your accounts at https://explore.plos.org/phishing.

If we can help with anything else, please email us at customercare@plos.org.

Thank you for submitting your work to PLOS ONE and supporting open access.

Kind regards,

PLOS ONE Editorial Office Staff

on behalf of

Dr. Zeyu Xing

Academic Editor

PLOS One
